# Chemoselective C‑Terminal Activation Platform for Direct Conversion of Native Linear Peptides into Thiazoline/Thiazole Macrocycles

**DOI:** 10.1021/acs.orglett.6c01073

**Published:** 2026-04-16

**Authors:** Bao Quang Gia Le, Minyoung Kwon, Monika Raj

**Affiliations:** Department of Chemistry, 1371 Emory University, Atlanta, Georgia 30322, United States

## Abstract

We report a sequence-independent, chemoselective C-terminal activation platform to synthesize thiazoline/thiazole macrocycles from native linear peptides. Selective C-terminal primary amide-to-nitrile conversion proceeds in solution on fully unprotected peptides with broad functional-group compatibility. This approach eliminates the need for presynthesized α-amino nitrile building blocks and minimizes epimerization risk. Our method enables rapid macrocyclization, supporting the direct total syntheses of Mollamide F, Sanguinamide A, and Haligramide A from linear precursors.

## Graphical abstract

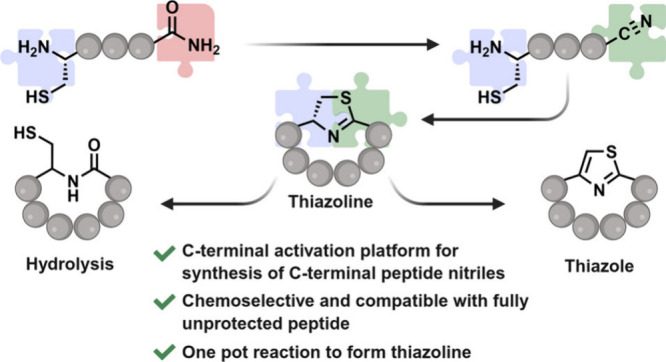

## Introduction

Macrocyclic peptides are an important modality for accessing targets that are challenging for small molecules while retaining greater structural tunability than biologics.^1–5^ In many bioactive marine macrocycles, such as Mollamide F, Sanguinamide A, and Haligramide A, thiazoline and thiazole heterocycles are embedded directly within the peptide backbone.^6,7^ Unlike external staples or side chain cyclizations, these backbone-embedded heterocycles drastically reduce conformational flexibility and mask polar amide bonds, thereby unlocking unique physical properties such as enhanced membrane permeability and proteolytic stability.^8–12^

Despite their value, accessing native thiazoline/thiazole backbone topologies remains challenging. Classical approaches rely on preinstalled heterocyclic building blocks during peptide assembly or on postsynthetic cyclodehydration conditions that can be harsh, low-yielding, and substrate-dependent.^13–16^ More recently, biocompatible N-terminal cysteine condensation with C-terminal nitriles has enabled thiazoline formation (and, upon oxidation, thiazoles), but these strategies typically require specialized synthesis of chiral α-amino nitrile precursors prior to peptide assembly (Figure 1A).^17,18^ Current methods are limited to the synthesis of hydrophobic amino acid nitriles without any reactive group on the side chain.^18^ This added synthesis slows library generation and may introduce epimerization risk, particularly when expanding to amino acids containing reactive side chains.^18^

**Figure 1:**
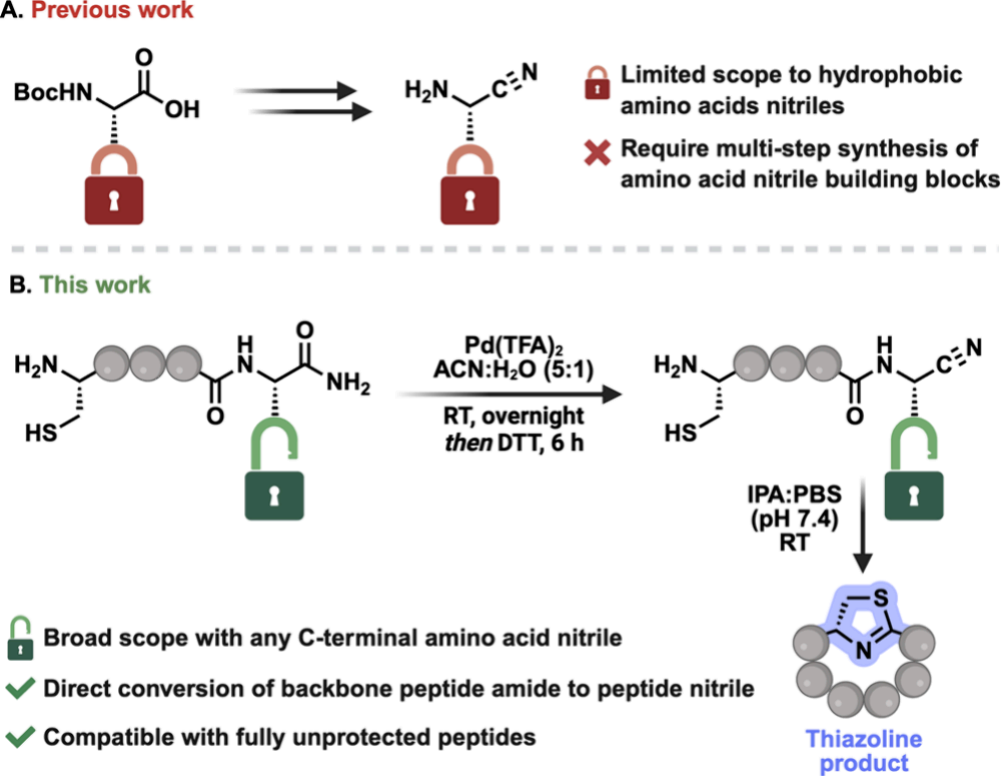
(A) Previous work requires multistep synthesis of individual amino acid nitriles and limited to C-terminal hydrophobic amino acids. (B) This work: Chemoselective C-terminal activation of C-terminal amide peptide to C-terminal nitrile peptide independent of the amino acid and their one pot cyclization to thiazoline cyclic peptide via N-terminal Cys-nitrile cyclization.

Here we report a late-stage, sequence-tolerant C-terminal activation strategy that converts fully assembled, unprotected peptides into thiazoline/thiazole macrocycles (Figure 1B). Using palladium­(II) trifluoroacetate (Pd­(TFA)_2_), we selectively dehydrate the C-terminal primary amide of native peptides to the corresponding nitrile at room temperature with high chemoselectivity, leaving common side-chain functional groups intact. Because the transformation is performed on completed peptides, stereochemical integrity is preserved without reliance on α-amino nitrile building blocks. We demonstrate rapid access to thiazoline/thiazole-containing macrocycles across 5–9 amino-acid ring sizes with 95–99% conversion and apply the platform to the total syntheses of Mollamide F, Haligramide A, and Sanguinamide A directly from their linear amide precursors. Consequently, this study establishes a universal platform for the construction of thiazoline and thiazole macrocycles, offering unrestricted access to bioactive peptide architectures.

## Results and Discussion

### Development of a Chemoselective C-Terminal Activation Platform

To establish a sequence-tolerant route to thiazoline/thiazole macrocycles, we first optimized the C-terminal activation sequence using a dipeptide model. Traditional methods for dehydrating primary amides to nitriles are often complicated by the presence of other nucleophilic or protic functional groups, presenting a significant challenge for complex biomolecules.^19,20^ To overcome this limitation, we drew inspiration from recent advances in palladium-catalyzed amide dehydrations, which are renowned for their exceptional chemoselectivity and ability to proceed under mild conditions. Thus, using Pd­(TFA)_2_ in acetonitrile enabled us to perform direct dehydration of the C-terminal primary amide to the corresponding nitrile on a dipeptide at room temperature as characterized by NMR and HRMS (Figure S1). This late-stage activation avoids external nitrile reagents and eliminates the need for presynthesized α-amino nitrile building blocks.^18^ The resulting nitrile dipeptide was screened for optimal condensation conditions with l-cysteine methyl ester hydrochloride by varying temperature, solvents, bases and amounts of Cys-methyl ester (Table S1). We found that a 1:1 mixture of IPA:PBS (pH 7.4) with N,N-diisopropylethylamine (DIPEA) facilitated the formation of thiazoline dipeptide in 95% conversion (Table S1). While DIPEA was necessary to neutralize the hydrochloride salt during this intermolecular step, we later observed that subsequent intramolecular cyclizations proceeded efficiently without additional base. Notably, during purification, we found that thiazolines are acid-labile and can hydrolyze under standard reverse-phase conditions containing acidic additives (e.g., 0.1% formic acid). Using acid-free purification protocols, thiazoline dipeptide was successfully isolated and characterized by NMR and HRMS (Figure S2).

### Downstream Reactivity: Hydrolysis and Thiazole Formation

With thiazoline dipeptide in hand, we examined its conversion to additional motifs. Exposure of thiazoline dipeptide to formic acid provided rapid hydrolysis to the corresponding native backbone peptide (Cys at the cyclization site) in 96% conversion as characterized by NMR and HRMS (Figure S3). To optimize thiazole formation, we screened a variety of reaction conditions, including different oxidants and oxidant loadings (Table S2). Literature-reported oxidation conditions employing MnO_2_ at 80 °C were ineffective for the purified dipeptide.^21^ Notably, inclusion of K_2_CO_3_ substantially enhanced the reaction rate, and systematic evaluation showed that K_2_CO_3_ alone or in combination with MnO_2_ at 80 °C was sufficient to furnish the thiazole dipeptide in >99% conversion, as confirmed by NMR and HRMS (Table S2, Figure S4).^22^

### Chemoselectivity and Cyclization Selectivity

After optimizing the dehydration protocol, we evaluated functional-group compatibility using peptide YWRMCKEHS, which contains a broad set of potentially reactive side chains. Treatment with Pd­(TFA)_2_ in acetonitrile at room temperature produced no detectable side-chain modifications by LC-MS, consistent with preferential reactivity toward peptide primary amide functionalities under these conditions (Figure S5).

Because Pd­(TFA)_2_ can also dehydrate asparagine/glutamine side-chain amides to the corresponding nitriles, we next asked whether an aliphatic side-chain nitrile could compete with the intended C-terminal nitrile in N-terminal cysteine cyclization. Two peptides with closely related sequences were prepared: peptide CWPAYA bearing a C-terminal primary amide, and peptide CWPAYQ bearing a C-terminal carboxylic acid and a glutamine residue. Exposure of each peptide to Pd­(TFA)_2_ in acetonitrile at room temperature afforded the corresponding nitrile products: a C-terminal backbone nitrile (CWPAYA-Nitrile) and a side-chain nitrile (CWPAYQ-Nitrile-COOH) (Figures S6 and S7).

Notably, during initial cyclization attempts, we observed poor thiazoline formation, consistent with palladium coordination to the peptide (including the N-terminal cysteine) that inhibits productive condensation. Addition of dithiothreitol (DTT) effectively scavenged palladium, forming an insoluble Pd-thiol complex that was removed by filtration to afford nitrile peptides suitable for cyclization (Figure 2). Upon solvent removal, both nitrile peptides were subjected to cyclization in IPA:PBS (pH 7.4). After stirring at room temperature, only the peptide bearing the C-terminal nitrile cyclized to give CWPAYA-thiazoline (Figure S6). In contrast, the peptide bearing a side-chain nitrile formed a cysteine disulfide dimer with no detectable thiazoline (Figure S7). These results demonstrate that thiazoline formation occurs selectively between N-terminal cysteine and the C-terminal nitrile, rather than aliphatic side-chain nitriles, enabling construction of backbone-embedded thiazoline macrocycles with native topology with high selectivity even in the presence of glutamine and asparagine.

**Figure 2:**
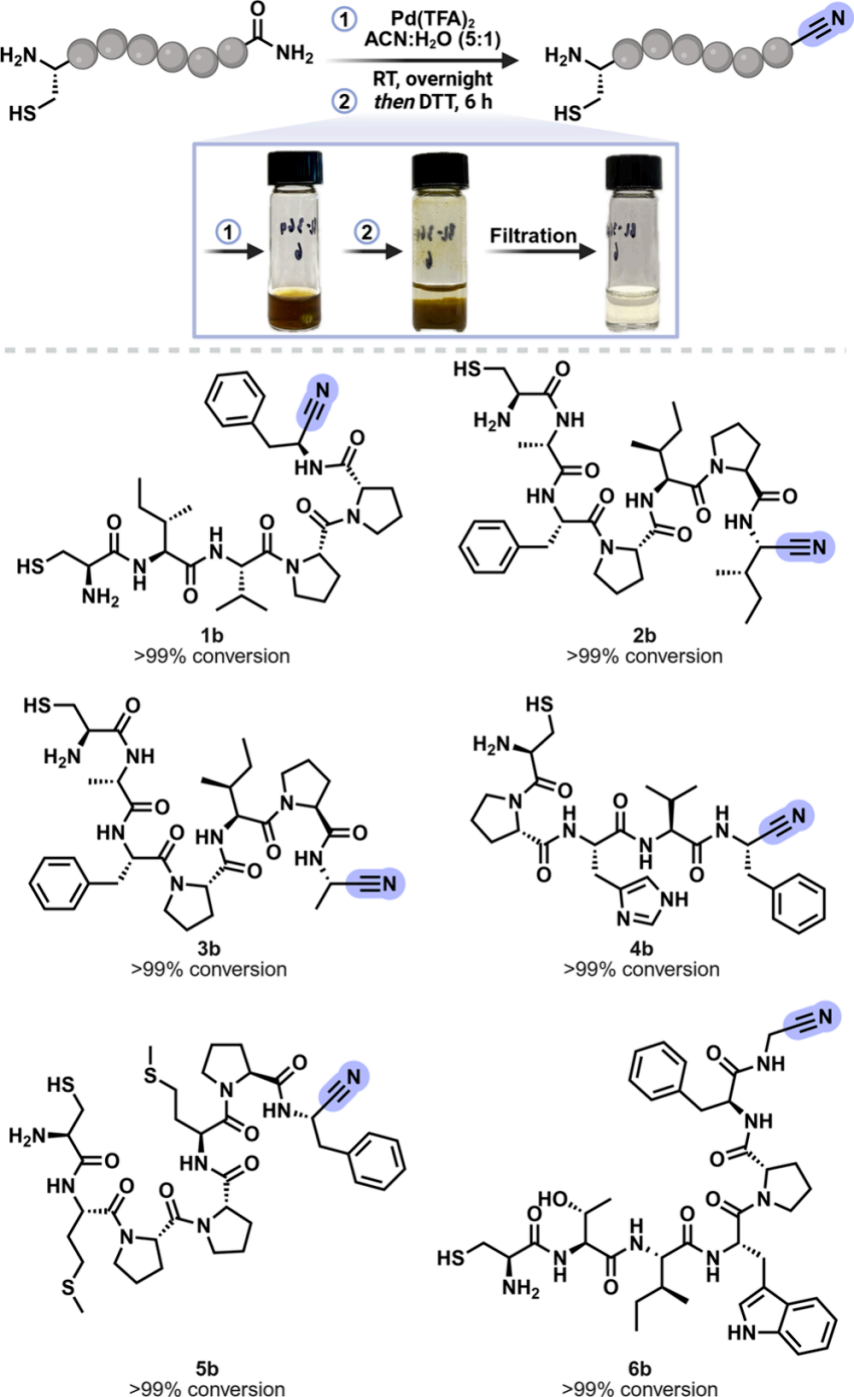
Peptide scope of converting backbone amide to backbone nitrile independent of amino acid at the C-terminus and the use of DTT for removing palladium from the reaction mixture.

Furthermore, the side-chain nitrile is reversible, treatment of peptide, WGNFL with Pd­(TFA)_2_ in acetonitrile converted the asparagine residue to its nitrile form, and subsequent exposure of the purified WGN-Nitrile-FL-COOH to Pd­(TFA)_2_ with acetamide in THF:H_2_O or IPA:H_2_O (1:1) at room temperature restored the asparagine native amide structure (Figure S8).

### Substrate Scope and Diversification to Varying Cyclic Peptides

We next applied the protocol to six peptides varying in sequence, C-terminal residue, and ring size, each bearing an N-terminal cysteine and prepared by SPPS on Rink resin (Figure 2). Pd­(TFA)_2_-mediated dehydration afforded the corresponding nitrile peptides **1b**–**6b** in high conversion (Figures S9–S14). Furthermore, to assess the robustness of the Pd­(TFA)_2_-mediated dehydration method with respect to the C-terminal amino acid, we also prepared C-terminal nitrile peptides containing diverse and potentially reactive C-terminal residues, including Cys, Arg, Trp, His, Ser, Tyr, Lys, Glu, and Met, achieving conversions of 47–99% (Table S3). Following palladium scavenging and solvent evaporation, the crude peptide nitriles (**1b**–**6b**) were directly subjected to cyclization in IPA:PBS (pH 7.4), providing thiazoline macrocycles **1c**–**6c** in similarly high conversion. This series included Mollamide F (**1c**), an anti-HIV macrocycle isolated from *Didemnum molle*, and **6c**, a structural analog of Phakellistatin 13 in which a native 5-membered proline ring is replaced with a thiazoline core (Figure 3).

**Figure 3:**
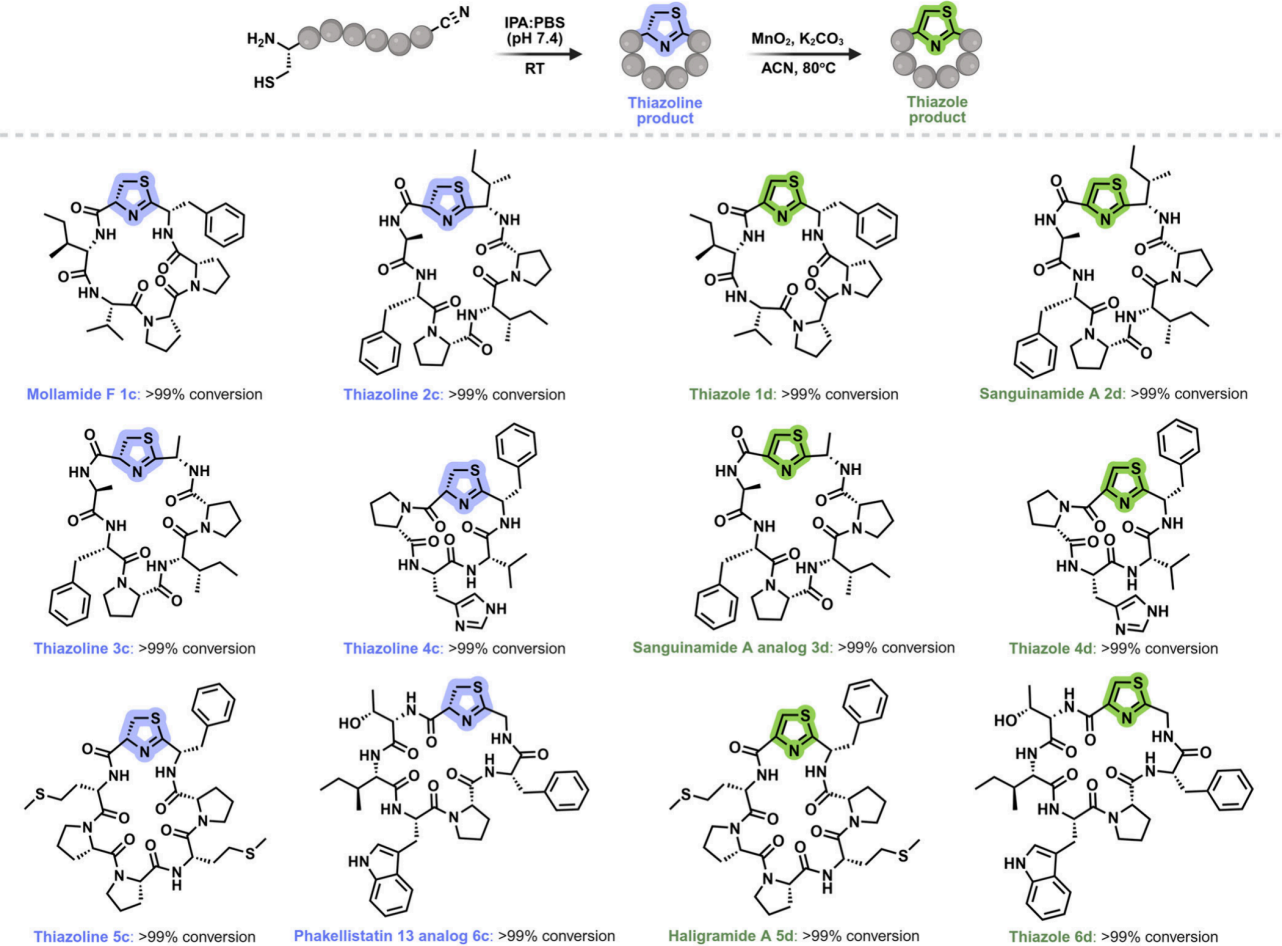
Peptide scope for the cyclization of backbone nitriles to thiazoline peptides and subsequent modification to thiazole peptides. Conversions to thiazoline and thiazole cyclic peptides are independent of peptide sequence and ring size.

The cyclization kinetics were notably dependent on the C-terminal residue. While most analogs (**1b**, **3b**–**6b**) cyclized at room temperature, the sterically hindered isoleucine derivative **2b** required heating (70 °C) for full conversion (Figure S10). In contrast, replacing the isoleucine with the less sterically hinder alanine (**3b**) restored room-temperature reactivity (Figure S11), highlighting the impact of steric congestion on the cyclization rate. To further demonstrate the robustness of cyclization with respect to C-terminal amino acids, we prepared additional thiazoline peptides bearing bulky, charged, and potentially reactive C-terminal residues, including Trp, Glu, Arg, and Met, all of which cyclized in 95–99% conversion (Table S4).

Next, we applied the optimized reaction conditions to convert thiazoline cyclic peptides to the corresponding thiazole macrocycles. Treatment with MnO_2_/ K_2_CO_3_ delivered thiazoles **1d**–**3d** and **5d** in quantitative conversion, including Sanguinamide A (**2d**) and Haligramide A (**5d**) (Figure 3, Figures S15–S20). Notably, peptide **5c** bears two Met residues that could be susceptible to oxidation, yet thiazoline **5c** was cleanly converted to Haligramide A (**5d**) in quantitative yield without any Met oxidation.

In contrast, oxidation of thiazolines **4c** and **6c** initially produced no detectable thiazole products. We hypothesized that the His residue in (**4c**) and the Trp residue in (**6c**) interact with MnO_2_, resulting in product loss during filtration. In support of this hypothesis, use of K_2_CO_3_ alone, without MnO_2_, enabled formation of thiazoles **4d** and **6d** in high conversion (Figure 3). Finally, the thazoline cyclic peptides were converted to the native cyclic peptides by treatment with formic acid, regenerating macrocycles **1e**–**6e** bearing a native amide backbone and Cys at the cyclization site (Figure 4, Figures S21–S26).

**Figure 4:**
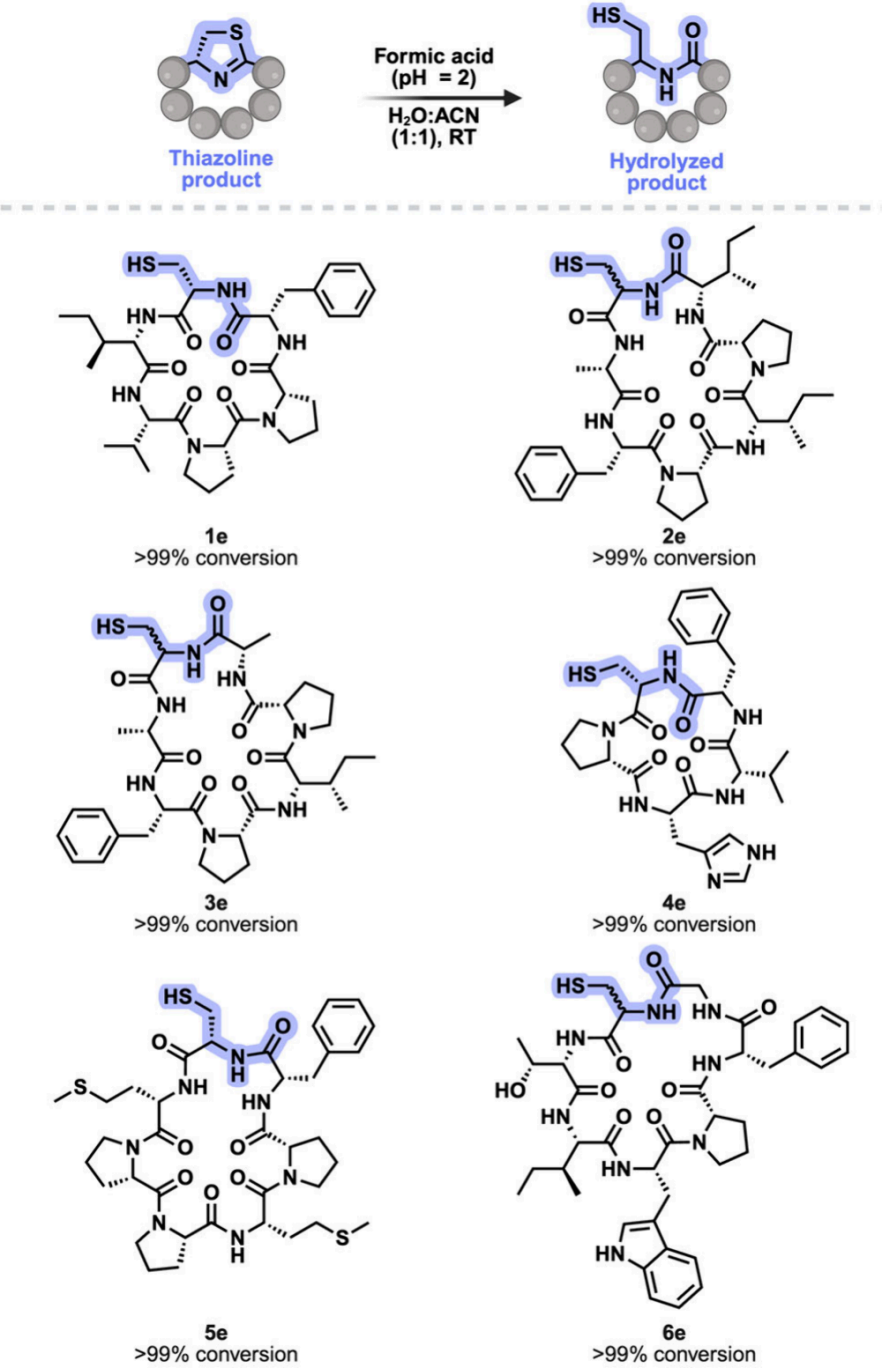
Peptide scope for the synthesis of native cyclic peptides from hydrolysis of thiazoline peptides. Full conversion to native cyclic peptides was observed independent of peptide sequence and ring size.

## Conclusion

We report a chemoselective C-terminal activation platform that converts native, fully unprotected linear peptides into thiazoline/thiazole macrocycles through late-stage C-terminal amide-to-nitrile dehydration using Pd­(TFA)_2_ under mild, solution-phase conditions. Palladium scavenging with DTT enables efficient N-terminal Cys cyclization, and chemoselectivity studies show that thiazoline formation occurs exclusively with a C-terminal backbone nitrile, while Asn/Gln-derived side-chain nitriles do not compete. The method is broadly compatible across sequences and ring sizes, delivers near-quantitative nitrile formation and high-conversion to thiazoline macrocycles, and supports downstream diversification to thiazole macrocycles or hydrolyzed native-backbone macrocycles. This streamlined approach eliminates specialized α-amino nitrile building blocks and provides rapid access to backbone-embedded heterocycles, enabling direct total syntheses of bioactive cyclic peptides, Mollamide F, Sanguinamide A, and Haligramide A directly from their linear precursors.

## Supplementary Material



## Data Availability

The data underlying this study are available in the published article and its Supporting Information.

## Notes

General experimental procedures and characterization details, including HPLC, HRMS, and ^1^H and ^13^C NMR spectra of all reported compounds (PDF)
