# Supplementary material for: Chemoselective C‑Terminal Activation Platform for Direct Conversion of Native Linear Peptides into Thiazoline/Thiazole Macrocycles
Source: Org Lett. 2026 Apr 16;28(17):5466–70. doi: 10.1021/acs.orglett.6c01073 (PMC13140135; doi:10.1021/acs.orglett.6c01073)
Supplement: Supplementary file 1 [file ol6c01073_si_001.pdf]

Supporting Information

Chemoselective C-terminal Activation Platform for Direct Conversion of Native Linear Peptides into Thiazoline/Thiazole Macrocycles

Bao Quang Gia Le, Minyoung Kwon, Monika Raj*

Department of Chemistry, Emory University, Atlanta, Georgia 30322, United States

Table of Contents

I. General. 3

II. Materials. 3

III. Purification. 3

IV. Instrumentation and sample analysis. 3

V. Fmoc-Solid Phase Peptide Synthesis. 4

VI. General Procedure A. Pd(II)-Mediated Conversion of C-Terminal Amide Peptides to Nitriles and Subsequent Cyclization to Thiazolines. 4

VII. General Procedure B. Oxidation of Thiazoline Peptides to Thiazoles. 5

VIII. General Procedure C. Hydrolysis of Thiazoline Peptides. 5

IX. Supplementary Figure 1. Converting C-terminal amide to C-terminal nitrile on dipeptide. 5

X. Supplementary Table 1. Optimization of Thiazoline Formation from C-Terminal Nitrile Dipeptides. 8

XI. Supplementary Figure 2. Formation of thiazoline dipeptide. 11

XII. Supplementary Figure 3. Hydrolysis of thiazoline dipeptide. 14

XIII. Supplementary Table 2. Optimization of Thiazole Formation from Thiazoline Dipeptides. 16

XIV. Supplementary Figure 4. Oxidation of thiazoline dipeptide to thiazole dipeptide. 18

XV. Supplementary Figure 5. Selectivity study of peptide YWRRSCKEHS. 20

XVI. Supplementary Figure 6. Selectivity study converting peptide CWPAYA-Amide to peptide CWPAYA-Nitrile and peptide CWPAYA-Thiazoline. 21

XVII. Supplementary Figure 7. Selectivity study on the conversion of peptide CWPAYQ-COOH to peptide CWPAYQ-Nitrile-COOH and the attempted formation of CWPAYQ-Thiazoline-COOH. 24

XVIII. Supplementary Figure 8. Study on the conversion of peptide WGNFL-COOH to peptide WGN-Nitrile-FL-COOH and the reformation of WGNFL-COOH. 27

XIX. Supplementary Table 3. Scope of C-terminal nitrile formation across diverse C-terminal amino acids with unprotected reactive side chains. 30

XX. Supplementary Figure 9. Converting peptide 1a to nitrile peptide 1b and thiazoline peptide 1c (Mollamide F). 44

XXI. Supplementary Figure 10. Converting peptide 2a to nitrile peptide 2b and thiazoline peptide 2c. 46

XXII. Supplementary Figure 11. Converting peptide 3a to nitrile peptide 3b and thiazoline peptide 3c. 48

XXIII. Supplementary Figure 12. Converting peptide 4a to nitrile peptide 4b and thiazoline peptide 4c. 50

XXIV. Supplementary Figure 13. Converting peptide 5a to nitrile peptide 5b and thiazoline peptide 5c. 52

XXV. Supplementary Figure 14. Converting peptide 6a to nitrile peptide 6b and thiazoline peptide 6c (Phakellistatin 13 analog). 54

XXVI. Supplementary Table 4. Scope of thiazoline formation across diverse C-terminal amino acids with unprotected reactive side chains. 56

XXVII. Supplementary Figure 15. Converting thiazoline peptide 1c to thiazole peptide 1d. 63

XXVIII. Supplementary Figure 16. Converting thiazoline peptide 2c to thiazole peptide 2d (Sanguinamide A). 64

XXIX. Supplementary Figure 17. Converting thiazoline peptide 3c to thiazole peptide 3d (Sanguinamide A analog). 65

XXX. Supplementary Figure 18. Converting thiazoline peptide 4c to thiazole peptide 4d. 66

XXXI. Supplementary Figure 19. Converting thiazoline peptide 5c to thiazole peptide 5d (Haligramide A). 67

XXXII. Supplementary Figure 20. Converting thiazoline peptide 6c to thiazole peptide 6d. 68

XXXIII. Supplementary Figure 21. Converting thiazoline peptide 1c to hydrolyzed peptide 1e. 69

XXXIV. Supplementary Figure 22. Converting thiazoline peptide 2c to hydrolyzed peptide 2e. 70

XXXV. Supplementary Figure 23. Converting thiazoline peptide 3c to hydrolyzed peptide 3e. 72

XXXVI. Supplementary Figure 24. Converting thiazoline peptide 4c to hydrolyzed peptide 4e. 73

XXXVII. Supplementary Figure 25. Converting thiazoline peptide 5c to hydrolyzed peptide 5e. 74

XXXVIII. Supplementary Figure 26. Converting thiazoline peptide 6c to hydrolyzed peptide 6e. 75

References 76

I. General. All commercial materials (Sigma-Aldrich, Fluka and Novabiochem) were used without further purification. All solvents were reagent or HPLC (Fisher) grade. All reactions were performed under air in glass vials or round bottom flasks. Conversions refer to chromatographically pure compounds; % conversions were obtained by comparing HPLC peak areas of products and starting materials. HPLC and HRMS were used to monitor reaction progress, and products were characterized using HRMS and NMR.

II. Materials. Fmoc-amino acids, Rink amide resin, 3[bis(dimethylamino)methyliumyl]-3H-benzotriazol-1-oxide hexafluorophosphate (HBTU), and N,N-Diisopropylethylamine (DIPEA) were obtained from CreoSalus (Louisville, Kentucky). Piperidine and trifluoroacetic acid (TFA), were obtained from Alfa Aesar (Ward Hill, Massachusetts). Potassium carbonate (K_2CO_3), N,Ndimethylformamide (DMF), dichloromethane (DCM), methanol (MeOH) and acetonitrile (ACN) were obtained from VWR (100 Matsonford Road Radnor, Pennsylvania). L-Cysteine methyl ester hydrochloride, manganese(IV) oxide were obtained from Sigma Aldrich (3050 Spruce Street, St. Louis, Missouri).Tris(2-carboxyethyl)phosphine hydrochloride (TCEP) was obtained from Combi-Blocks (7949 Silverton Ave #915, San Diego, California). Dithiothreitol (DTT) and formic acid (FA) was obtained from Oakwood Chemical (730 Columbiaa Hwy N. Estill, South Carolina). Palladium(II) 2,2,2-trifluoroacetate was obtained from AmBeed (3205 N Wilke Rd, Ste3205-125)

III. Purification. HPLC: Purification of peptides was performed using preparatory high performance liquid chromatography (HPLC) on an ACCQ Prep HP 150 equipped with a 10 x 150 mm RediSep Prep C18Aq, 100A, 5 µm column. Separations involved a mobile phase of 0.1% FA in water (solvent A) and 0.1 % FA in acetonitrile (solvent B) or mobile phase of water (solvent A) and acetonitrile (solvent B). The eluent was monitored by absorbance at 220 nm.

HPLC Method A: Gradient: 0 to 80% **B** (with no FA) in 40 min; 80 to 100% **B** in 40.1 to 45 min at a flow rate of 4.7 mL min^{-1}.

HPLC Method B: Gradient: 0 to 80% **B** (0.1 % FA) in 40 min; 80 to 100% **B** in 40.1 to 45 min at a flow rate of 4.7 mL min^{-1}.

IV. Instrumentation and sample analysis. NMR. ^{1}H and ^{13}C spectra were acquired at 25 °C in DMSO-d_6, using a Bruker 400 MHz spectrometer. All ^{1}H NMR chemical shifts (δ) were referenced relative to the residual DMSO-d_6 peak at 2.50 ppm. ^{13}C NMR chemical shifts were referenced to DMSO-d_6 at 39.52 ppm. ^{13}C NMR spectra were proton decoupled. NMR spectral data are reported as chemical shift (multiplicity, coupling constants (*J*), integration). Multiplicity is reported as follows: singlet (s), doublet (d), doublet of doublets (dd), doublet of triplets (td), triplet (t), and multiplet (m). Coupling constant (*J*) in hertz (Hz).

Analytical HPLC. Analytical HPLC was performed on an Agilent 1100 series HPLC equipped with a 4.6 x 150 mm RediSep Prep C18Aq, 100A, 5 µm column. The reaction was monitored by analytical reverse phase HPLC using a gradient of water versus acetonitrile in linear gradients with a constant flow rate of 1 mL min^{-1}. Separations involved a mobile phase of 0.1% formic acid in water (solvent A) and 0.1% formic acid in acetonitrile (solvent B) or mobile phase of water (solvent A) and acetonitrile (solvent B). The eluent was monitored with a detection wavelength of 220 nm.

Analytical HPLC Method 1a: Gradient: 2 to 80% B (0.1% formic acid in ACN) in 30 min at a flow rate of 1 mL min^{-1}.

Analytical HPLC Method 1b: Gradient: 2 to 80% B (ACN with no formic acid) in 30 min at a flow rate of 1 mL min^{-1}.

Analytical HPLC Method 2a: Gradient: 2 to 60% B (0.1% formic acid in ACN) in 30 min at a flow rate of 1 mL min^{-1}.

Analytical HPLC Method 2b: Gradient: 2 to 60% B (ACN with no formic acid) in 30 min at a flow rate of 1 mL min^{-1}.

Analytical HPLC Method 3a: Gradient: 2 to 40% B (0.1% formic acid in ACN) in 30 min at a flow rate of 1 mL min^{-1}.

Analytical HPLC Method 3b: Gradient: 2 to 40% B (ACN with no formic acid) in 30 min at a flow rate of 1 mL min^{-1}.

Analytical HPLC Method 4: Gradient: 2 to 80% B (0.1% formic acid in ACN) in 10 min at a flow rate of 1 mL min^{-1}.

HRMS. High resolution MS data were acquired on a ThermoFisherLTQ Orbitrap Velos mass spectrometer with a heated electrospray source. For direct infusion experiments, the solution was infused at a rate of 300 µl min-1 and the positive ion spray voltage was set to 3.0 kV. The instrument parameters were as follows: scan range = 200-2000 m/z; capillary temp = 320 °C, RF lens = 60%. Spectra were taken at 60,000 resolutions at m/z 200 using Tune software and analyzed with Thermo's Freestyle software.

V. Fmoc-Solid Phase Peptide Synthesis. All starting peptides were manually synthesized on Rink Amide using standard protocols.[1] Resin was swelled in DCM for 1 hour. For the initial Fmoc deprotection, the swelling solution was replaced with 20% piperidine in DMF and the resin was placed on a wrist action shaker for 20 minutes at room temperature. After Fmoc deprotection, the resin was washed twice with DMF, MeOH, and DCM. Subsequent amino acid couplings were performed using 5 equivalents of Fmoc-protected amino acid, HBTU, and DIPEA in DMF, shaking for 30 minutes at room temperature. Iterative deprotection and coupling steps were performed until the full sequence was achieved. Fmoc deprotection was reduced to 15 minutes for these subsequent steps. Peptides were cleaved from the resin using 4 mL of a cocktail consisting of 98:2.5:2.5 trifluoroacetic acid : water : triethylsilane (TES) for 2 h. The resin was removed by filtration, and the resulting solution was concentrated. Peptides were precipitated and centrifugated with cold diethyl ether (3 x 10 mL) to obtain the crude product. Crude peptides were dissolved in ACN:H_2O and purified by preparatory HPLC.

VI. General Procedure A. Pd(II)-Mediated Conversion of C-Terminal Amide Peptides to Nitriles and Subsequent Cyclization to Thiazolines.

In a 1 dram vial equipped with a magnetic stirrer was added the C-terminal amide peptide (0.01 mmol, 1 eq), palladium(II) trifluoroacetate ($Pd(TFA)_2$) (10 mg, 0.03 mmol, 3 eq), ACN (1 mL), and H_2O (200 µL).

The resulting mixture was allowed to react overnight at room temperature. Subsequently, dithiothreitol (DTT) (46 mg, 0.3 mmol, 30 eq) was introduced to the reaction mixture and stirred for 6 hours. After filtration to remove the yellow precipitate, a clear, colorless solution containing the nitrile peptide was obtained. The conversion to the nitrile products was determined via analytical HPLC using 0.1% formic acid (FA) in both mobile phases A and B. Following analysis, the solvent was removed via the lyophilizer, and the resulting residue was redissolved in a 1:1 mixture of IPA (0.5 mL) and PBS buffer (0.5 mL, pH 7.4). The reaction was stirred overnight at room temperature. Conversion to the thiazoline product was determined via analytical HPLC using FA-free mobile phases A and B. The thiazoline product was subsequently purified using this same method.

VII. General Procedure B. Oxidation of Thiazoline Peptides to Thiazoles.

In a 1 dram vial equipped with a magnetic stirrer was added the purified thiazoline peptide (1 eq), MnO_2 (10 eq), K_2CO_3 (10 eq), and ACN (0.5 mL). The resulting mixture was stirring overnight at 80 °C. Upon completion, the reaction was cooled to room temperature and filtered. Analysis via analytical HPLC, using FA-free mobile phases A and B, was used to determine the conversion of the thiazole product.

VIII. General Procedure C. Hydrolysis of Thiazoline Peptides.

To a 1-dram vial equipped with a magnetic stirrer was added the purified thiazoline peptide (1 mg), ACN (0.5 mL), H_2O (0.5 mL), and FA (20 µL). The resulting mixture was stirred overnight at room temperature. Upon completion, the reaction was analyzed via analytical HPLC, using FA-free mobile phases A and B, to determine the conversion to the hydrolyzed product.

IX. Supplementary Figure 1. Converting C-terminal amide to C-terminal nitrile on dipeptide.

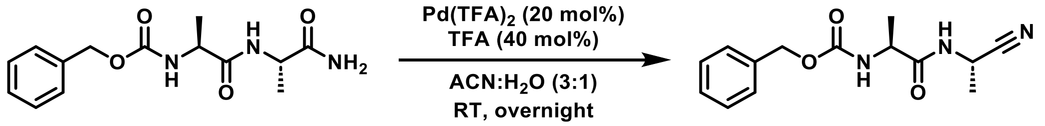

Procedure

To a 50 mL round-bottom flask equipped with magnetic stirrer were added benzyl ((S)-1-(((S)-1-amino-1-oxopropan-2-yl)amino)-1-oxopropan-2-yl)carbamate (400 mg, 1.36 mmol, 1 eq), palladium(II) trifluoroacetate (91 mg, 0.27 mmol, 0.2 eq), acetonitrile (50 mL), water (10mL), and trifluoroacetic acid (42 µL,0.54 mmol, 0.4 eq). The mixture was allowed to react overnight at room temperature. After the reaction was completed, brine (500 mL) was added. The mixture was extracted with CH_2Cl_2 (3 x 250 mL). The combined extracts were washed with brine (300 mL), dried over anhydrous Na_2SO_4, filtered, and concentrated *in vacuo*. The crude reaction mixture was purified by silica column chromatography (6% MeOH in CH_2Cl_2 eluent) to obtain the pale, yellow solid (265 mg, 71% isolation yield). **^{1}H NMR** (400 MHz, DMSO) δ 8.68 (d, J = 7.2 Hz, 1H), 7.55 (d, J = 7.5 Hz, 1H), 7.41 – 7.27 (m, 5H), 5.08 – 4.95 (m, 2H), 4.75 (p, J = 7.2 Hz, 1H), 4.02 (p, J = 7.2 Hz, 1H), 1.42 (d, J = 7.2 Hz, 3H), 1.21 (d, J = 7.2 Hz, 3H). **^{13}C NMR** (101 MHz, DMSO) δ 172.5, 155.7, 137.0, 128.3, 127.8, 120.3, 65.4, 49.8, 35.6, 18.1, 17.8. **HRMS (ESI)** m/z: $[M + H]^+$ Calcd for $C_{14}H_{18}N_3O_3$ 276.1348, Found 276.1342; $[M + Na]^+$ Calcd for $C_{14}H_{17}N_3O_3Na$ 298.1168, Found 298.1161; $[M + K]^+$ Calcd for $C_{14}H_{17}N_3O_3K$ 314.0907, Found 314.0901.

^{1}H NMR spectrum of nitrile dipeptide

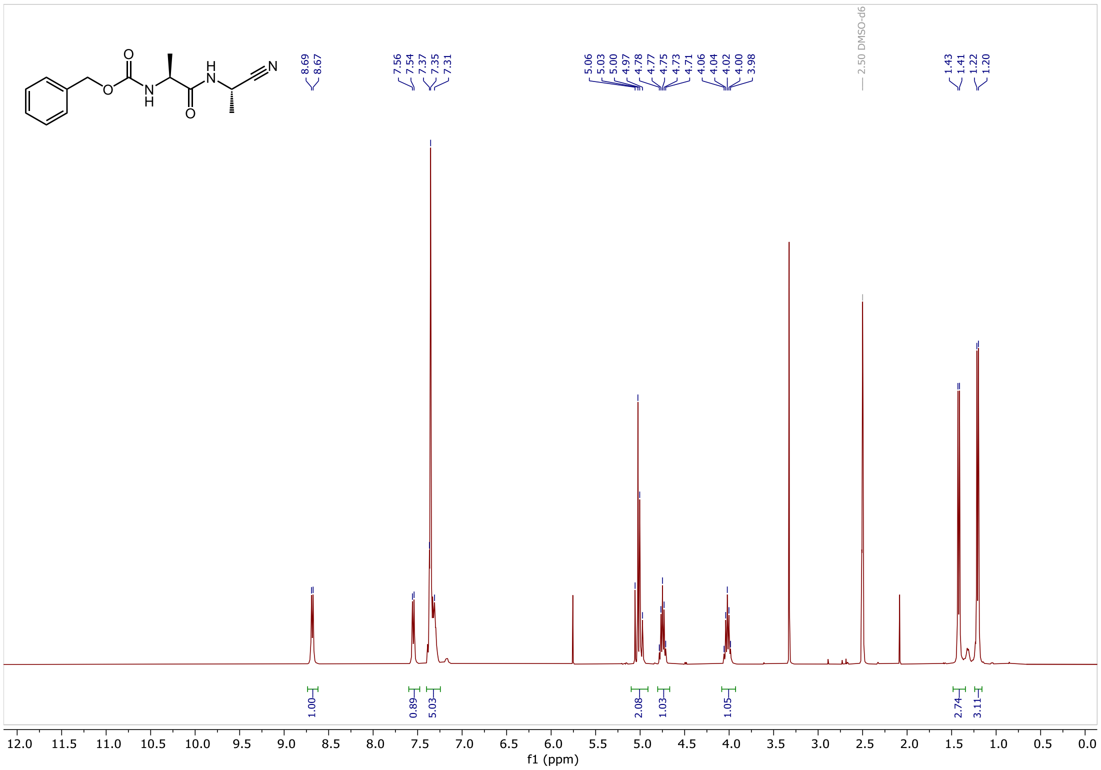

^{13}C NMR spectrum of nitrile dipeptide

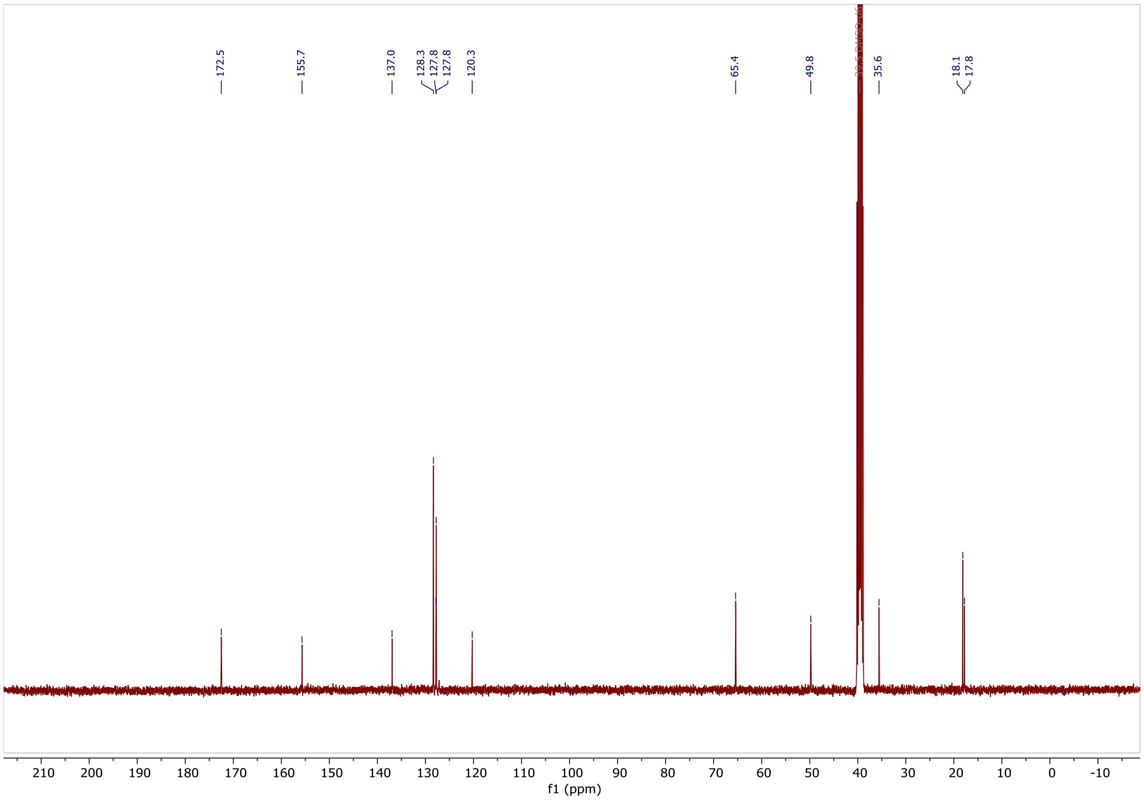

HRMS spectrum of purified nitrile dipeptide

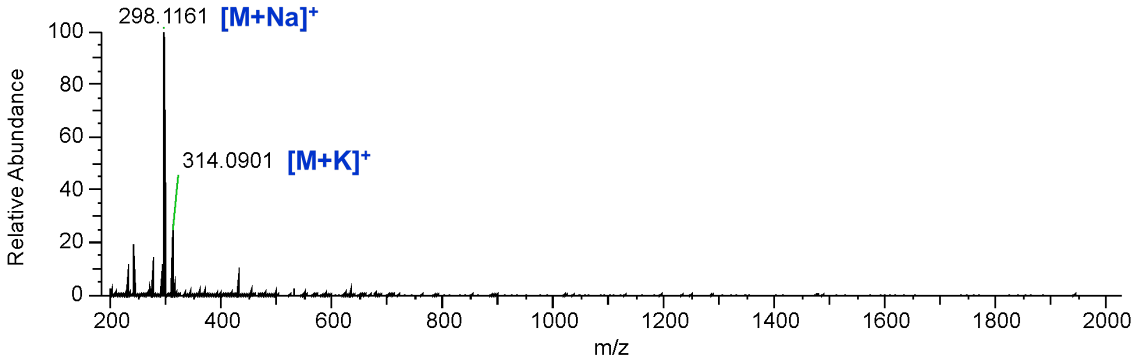

Zoom in HRMS spectrum of purified nitrile peptide

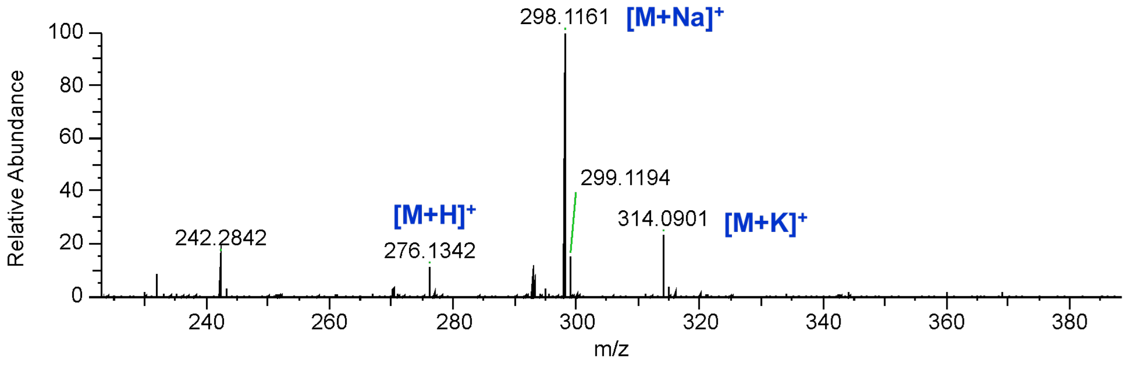

X. Supplementary Table 1. Optimization of Thiazoline Formation from C-Terminal Nitrile Dipeptides.

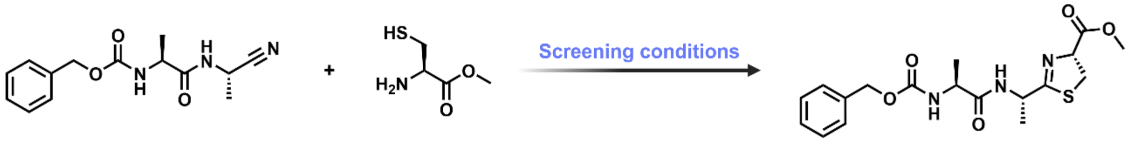

Entry	L-Cysteine methyl ester hydrochloride	K_2CO_3	DIPEA	Solvents	Temperature (°C)	Conversion (%)
**1**	2 eq	-	-	THF:DMF (10:1)	RT	0
**2**	2 eq	-	-	THF:H_2O (1:1)	RT	0
**3**	2 eq	3 eq	-	THF:DMF (10:1)	RT	0
**4**	2 eq	3 eq	-	THF:H_2O (1:1)	RT	0
**5**	2 eq	3 eq	-	ACN	RT	0
**6**	2 eq	3 eq	-	ACN	40	9
**7**	2 eq	3 eq	-	ACN	70	65
**8**	5 eq	3 eq	-	ACN	RT	13
**9**	5 eq	3 eq	-	ACN	70	56
**10**	5 eq	3 eq	-	ACN:H_2O (2:1)	70	63
**11**	5 eq	1 eq	-	ACN:H_2O (2:1)	70	53
**12**	5 eq	1 eq	-	IPA:PBS (1:1)	70	50
**13**	5 eq	1 eq	-	IPA:PBS (1:1)	RT	94
**14**	5 eq	-	2 eq	IPA:PBS (1:1)	RT	95

Procedure

The purified C-terminal nitrile dipeptide (0.018 mmol, 1 eq) was added to a 1 dram vial equipped with a magnetic stirrer for screening under the conditions listed in Table 1. The reaction was allowed to proceed overnight. The resulting crude mixture was analyzed via **Analytical HPLC Method 1b** to determine the conversion. Under these conditions, the C-terminal nitrile dipeptide and the thiazoline dipeptide exhibited retention times of 16.0 min and 17.3 min, respectively.

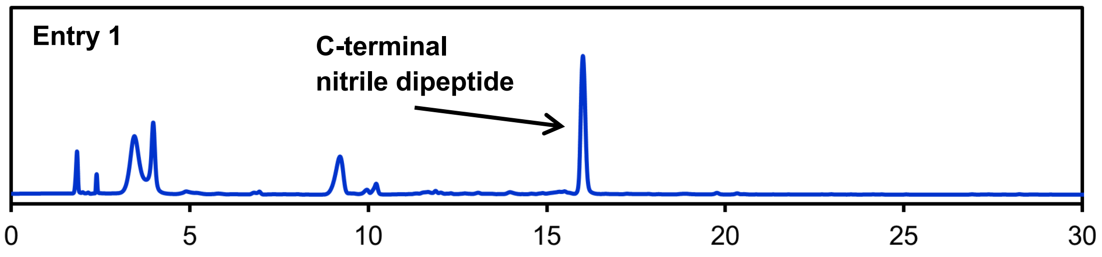

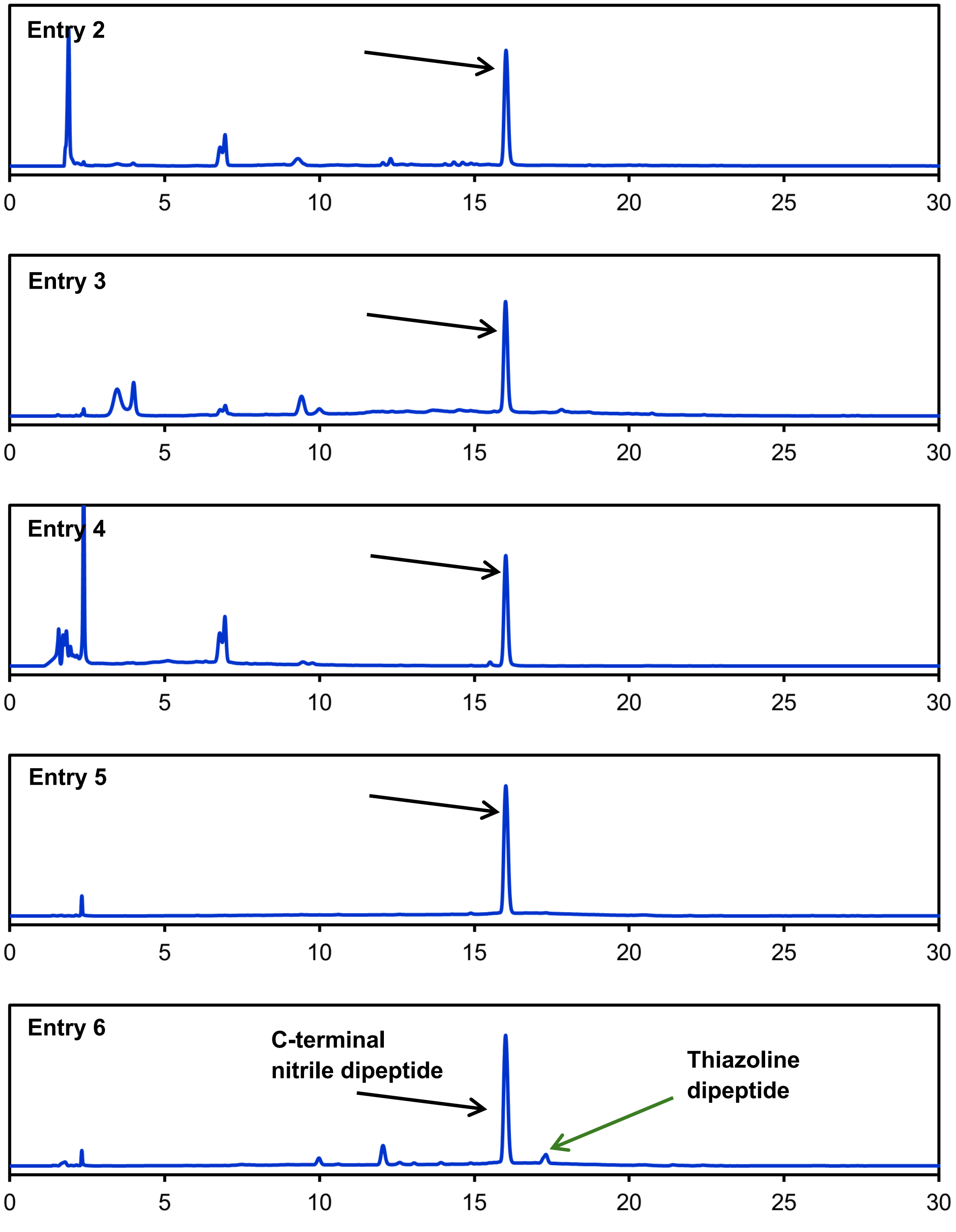
Entry 2
0 5 10 15 20 25 30
Entry 3
0 5 10 15 20 25 30
Entry 4
0 5 10 15 20 25 30
Entry 5
0 5 10 15 20 25 30
Entry 6
C-terminal
nitrile dipeptide
Thiazoline
dipeptide
0 5 10 15 20 25 30

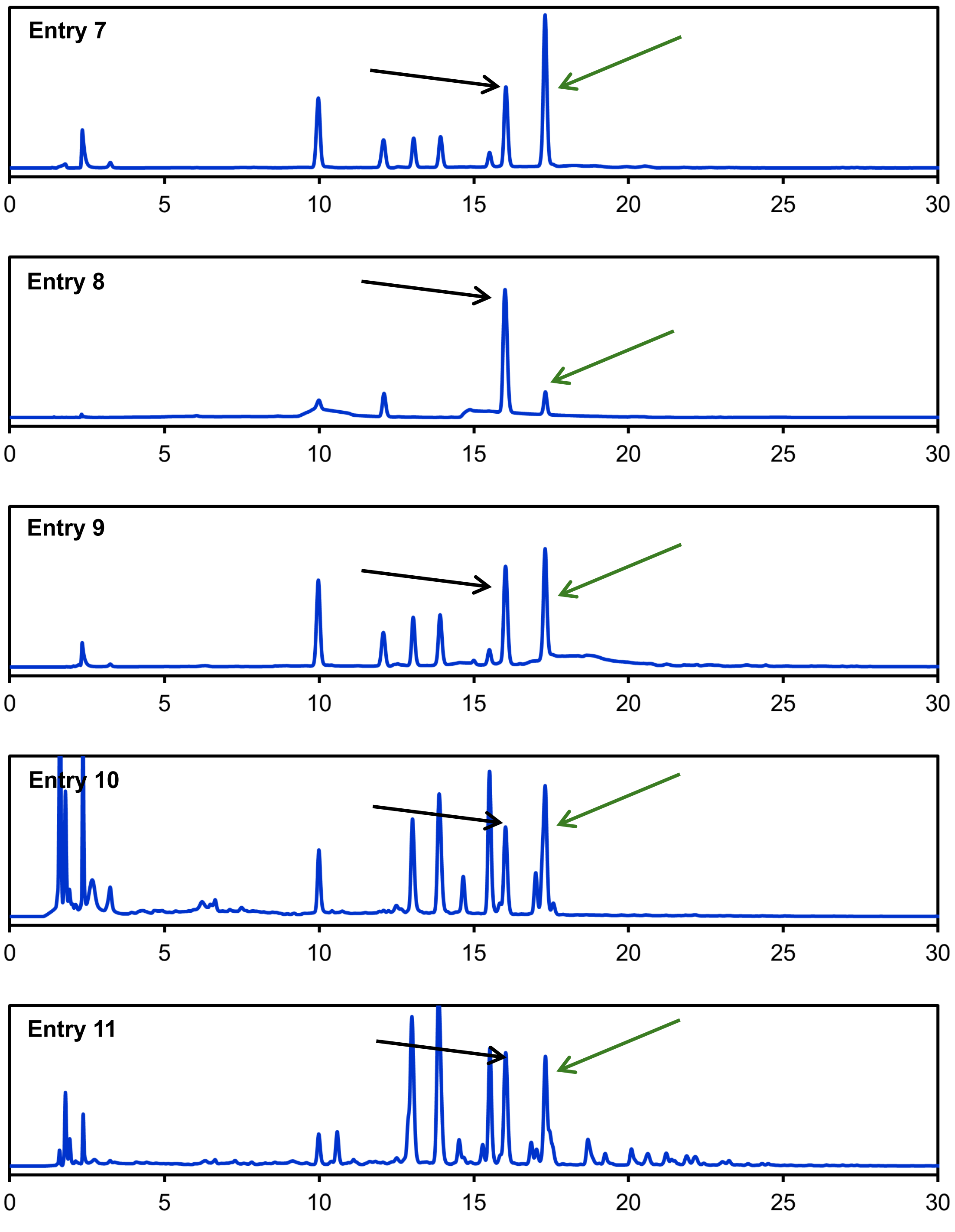

Entry 7
0
5
10
15
20
25
30
Entry 8
0
5
10
15
20
25
30
Entry 9
0
5
10
15
20
25
30
Entry 10
0
5
10
15
20
25
30
Entry 11
0
5
10
15
20
25
30

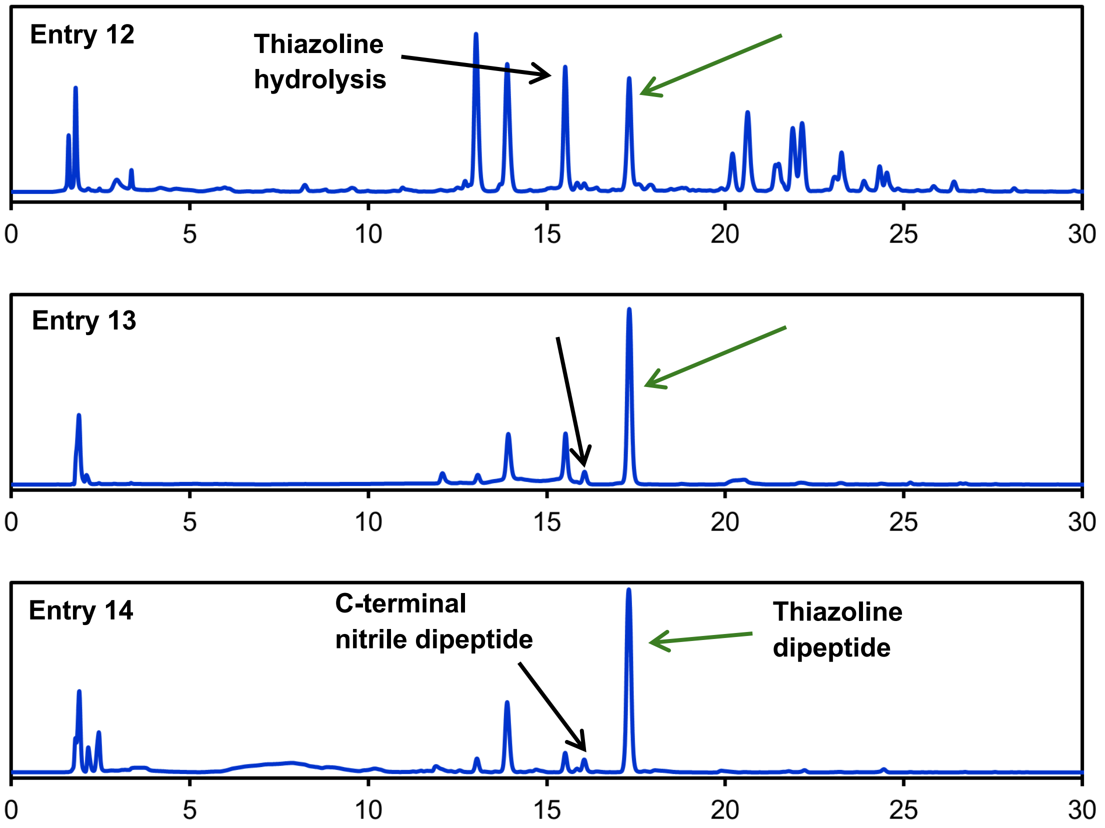

XI. Supplementary Figure 2. Formation of thiazoline dipeptide.

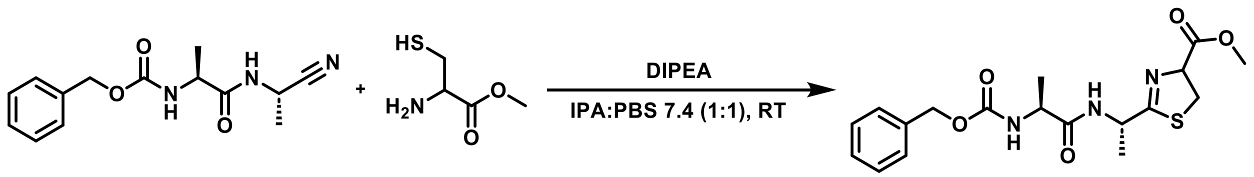

Procedure

To a 25 mL round-bottom flask equipped with magnetic stirrer were added nitrile dipeptide (50 mg, 0.18 mmol, 1 eq), L-Cysteine methyl ester hydrochloride (156 mg, 0.91 mmol, 5 eq), DIPEA (63 µL, 0.36 mmol, 2 eq), IPA (4 mL), and PBS buffer (pH = 7.4) (4 mL). The mixture was allowed to react overnight at room temperature. After the reaction was completed, the solvent was removed *in vacuo*. The crude reaction mixture was analyzed via **Analytical HPLC Method 1b** to determine the conversion of the reaction (95% conversion) and was purified by **HPLC Method A** to obtain the pale-yellow gel (62.3 mg, 87% isolation yield). **^{1}H NMR** (400 MHz, DMSO) δ 8.41 (dd, *J* = 22.4, 8.0 Hz, 1H), 7.43 (d, *J* = 7.8 Hz, 1H), 7.40 – 7.13 (m, 5H), 5.21 – 5.10 (m, 1H), 5.02 (d, *J* = 3.1 Hz, 2H), 4.66 (p, *J* = 6.8, 5.8 Hz, 1H), 4.16 – 3.97 (m, 1H), 3.59 – 3.48 (m, 1H), 3.42 (d, *J* = 11.4 Hz, 1H), 1.31 (t, *J* = 7.3 Hz, 3H), 1.22 (t, *J* = 7.9 Hz, 3H). **^{13}C NMR** (101 MHz, DMSO) δ 176.9, 172.1, 170.8, 155.6, 137.1, 128.3, 127.7, 127.7, 78.1, 65.3, 52.3, 49.8, 46.9,

34.3, 19.1, 18.1. **HRMS (ESI)** m/z: $[M + H]^+$ Calcd for $C_{18}H_{24}N_3O_5S$ 394.1437, Found 394.1427; $[M + Na]^+$ Calcd for $C_{18}H_{23}N_3O_5SNa$ 416.1256, Found 416.1246.

<u>^{1}H NMR spectrum of thiazoline dipeptide</u>

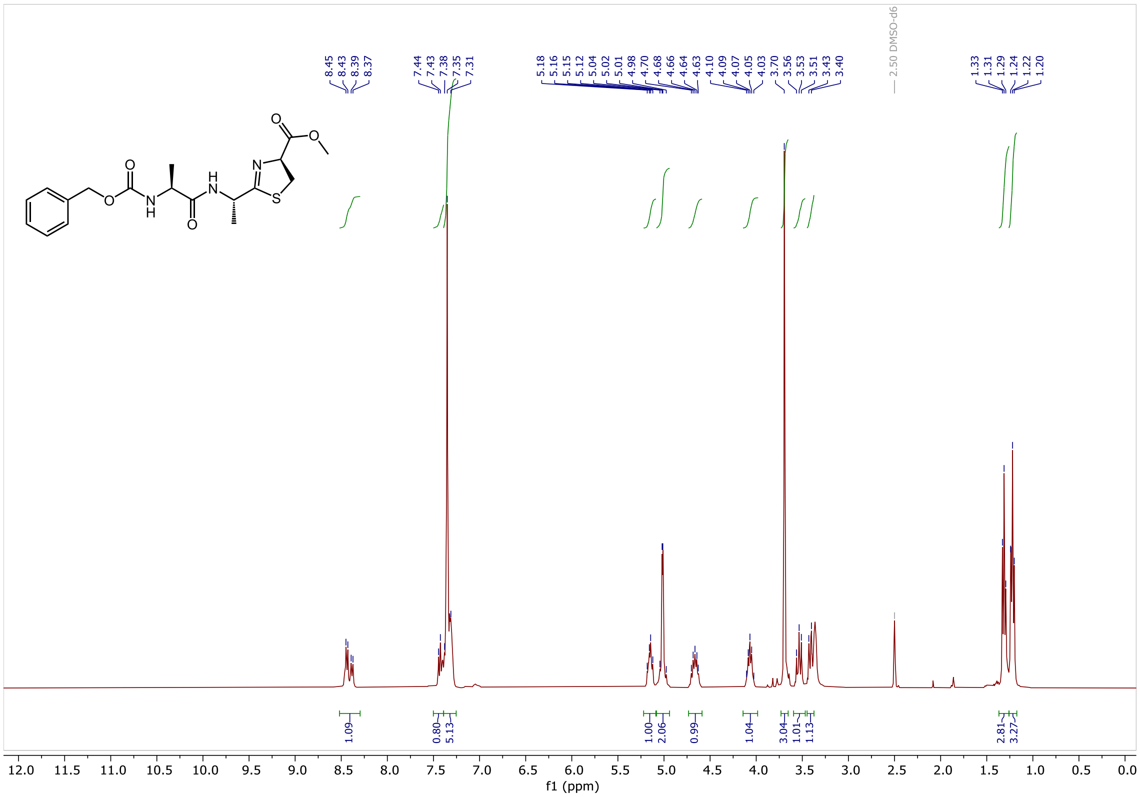

<u>^{13}C NMR spectrum of thiazoline dipeptide</u>

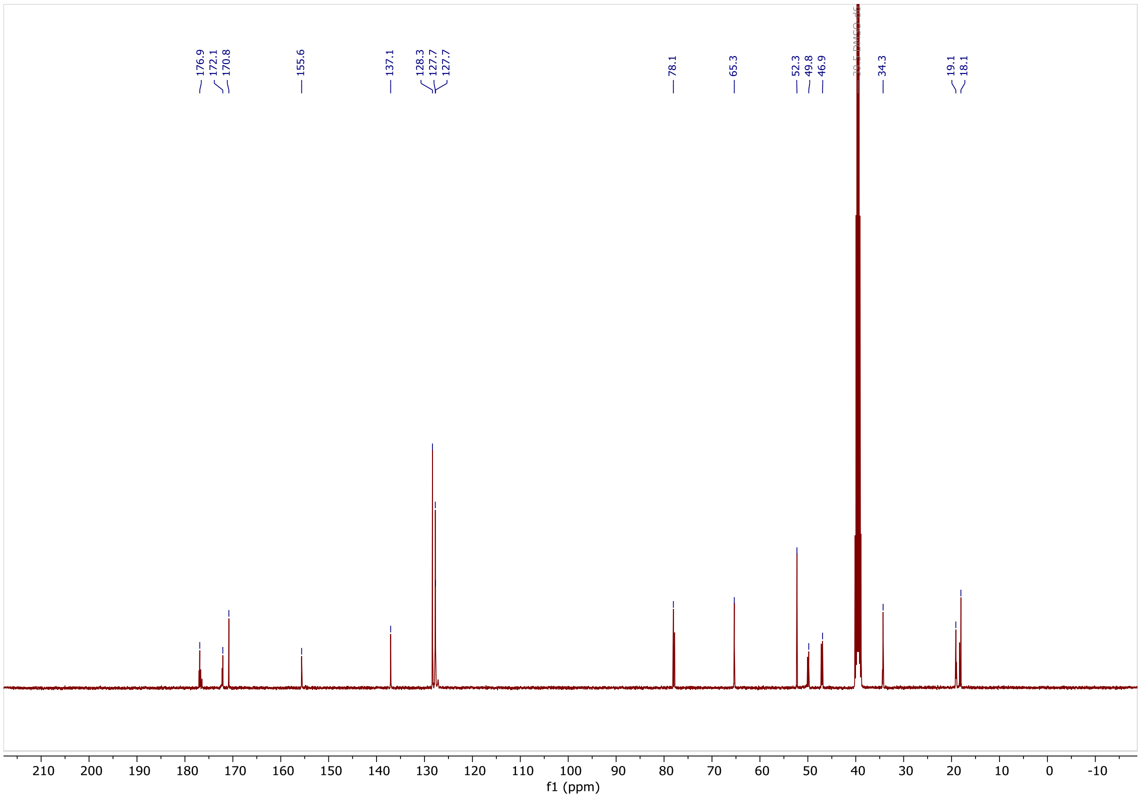

<u>*HRMS spectrum of purified thiazoline dipeptide*</u>

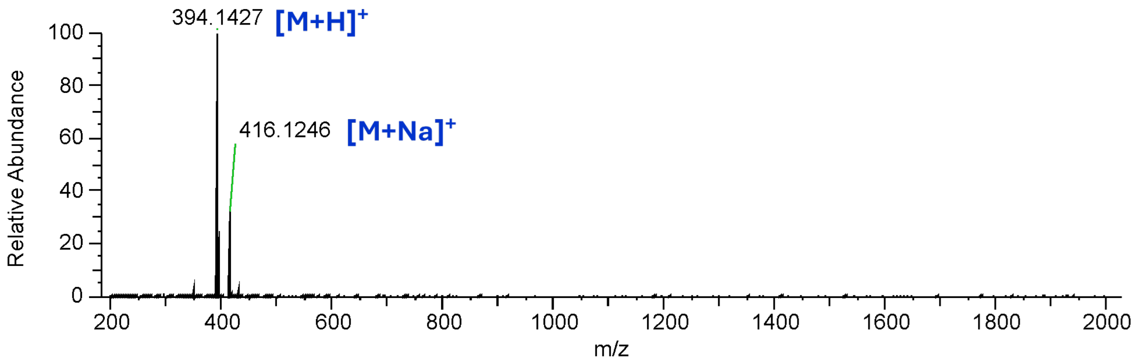

XII. Supplementary Figure 3. Hydrolysis of thiazoline dipeptide.

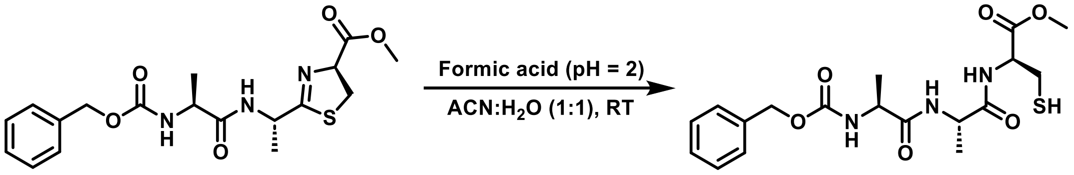

Procedure

To a 6 dram vial equipped with magnetic stirrer were added thiazoline dipeptide (10 mg, 0.025 mmol, 1 eq), ACN (1 mL), water (1 mL), and formic acid (20 μL). The mixture was allowed to react overnight at room temperature. After the reaction was completed, the crude reaction mixture was analyzed via **Analytical HPLC Method 1b** to determine the conversion of the reaction (96% conversion) and was purified by **HPLC Method B** to obtain a white powder (9.5 mg, 91% isolation yield). ^{1}**H NMR** (400 MHz, DMSO) δ 8.25 (d, J = 7.8 Hz, 1H), 8.05 (d, J = 7.4 Hz, 1H), 7.47 (d, J = 7.6 Hz, 1H), 7.40 – 7.25 (m, 5H), 5.01 (d, J = 3.4 Hz, 2H), 4.47 (td, J = 7.3, 4.9 Hz, 1H), 4.32 (p, J = 7.1 Hz, 1H), 4.06 (p, J = 7.2 Hz, 1H), 3.64 (s, 3H), 2.81 (qd, J = 13.8, 6.1 Hz, 2H), 1.23 (d, J = 7.0 Hz, 3H), 1.19 (d, J = 7.2 Hz, 3H).^{13}**C NMR** (101 MHz, DMSO) δ 172.5, 172.2, 170.5, 155.9, 137.0, 128.4, 127.8, 127.7, 65.5, 54.4, 52.2, 50.1, 48.3, 25.4, 18.2, 17.8. **HRMS (ESI)** m/z: $[M + H]^+$ Calcd for $C_{18}H_{26}N_3O_6S$ 412.1542, Found 412.1537; $[M + Na]^+$ Calcd for $C_{18}H_{25}N_3O_6SNa$ 434.1362, Found 434.1355; $[M + K]^+$ Calcd for $C_{18}H_{25}N_3O_6SK$ 450.1101, Found 450.1093.

<u>*^{1}H NMR spectrum of hydrolyzed dipeptide*</u>

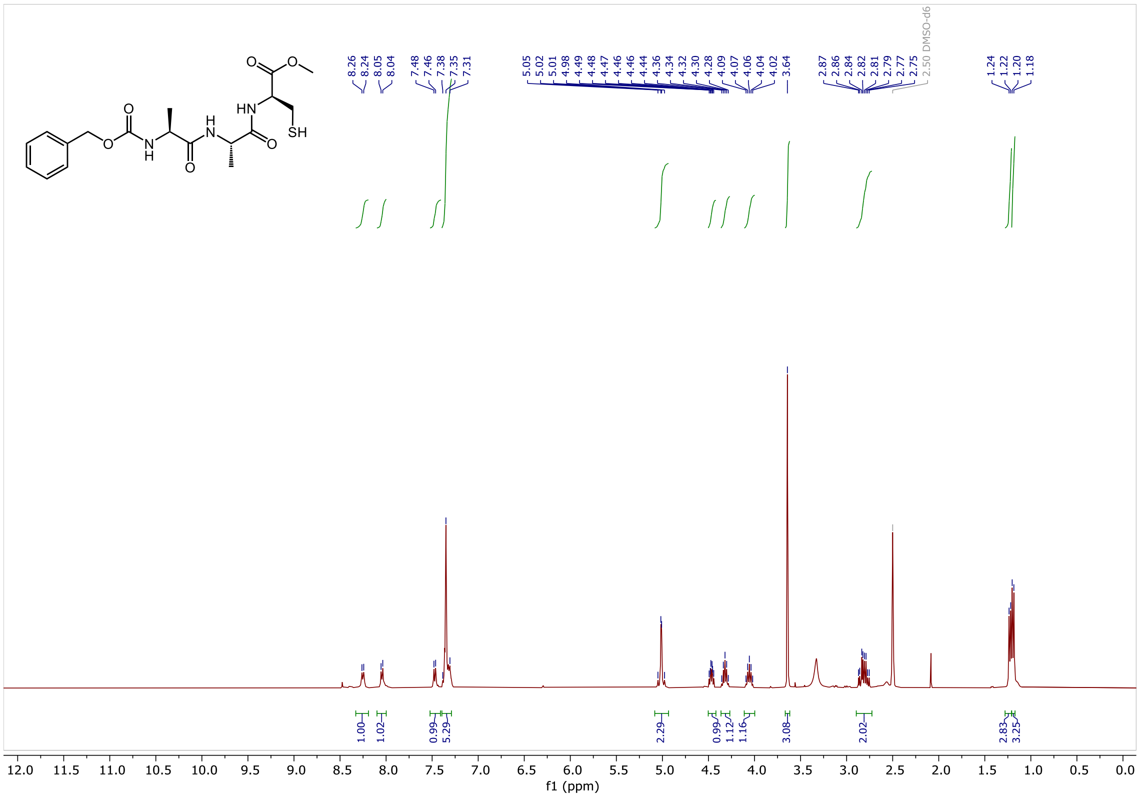

^{13}C NMR spectrum of hydrolyzed dipeptide

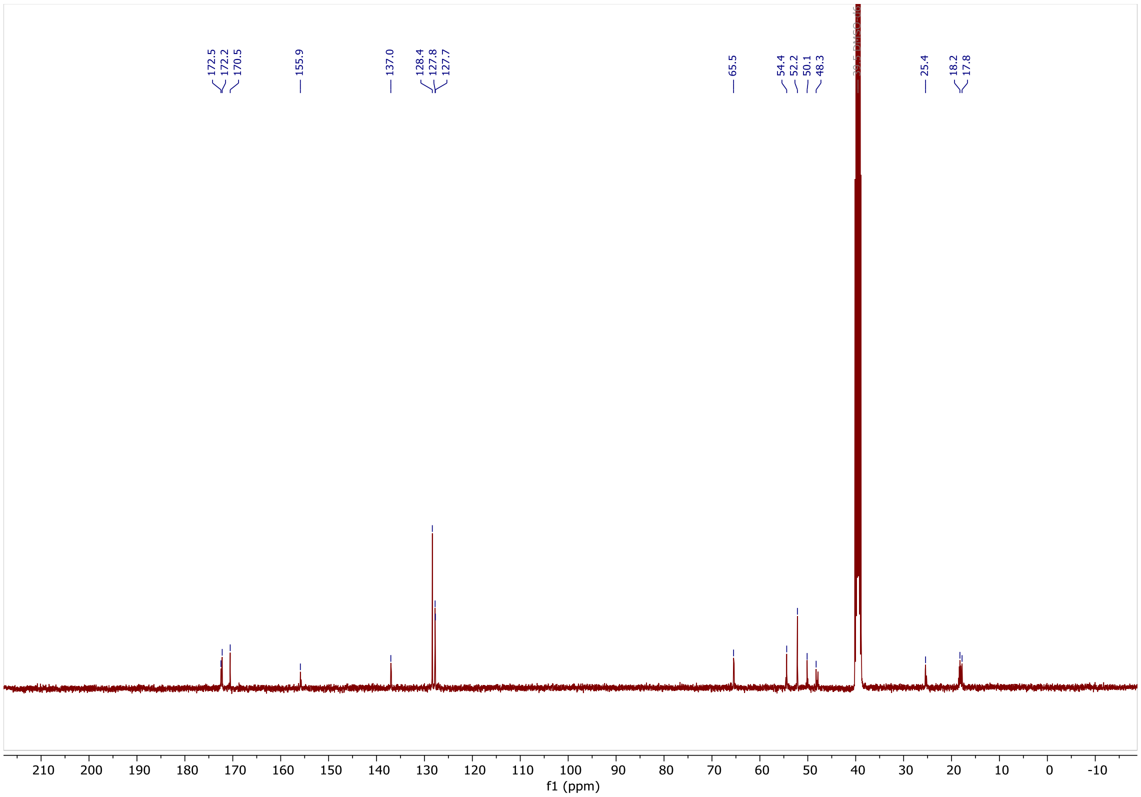

HRMS spectrum of purified hydrolyzed dipeptide

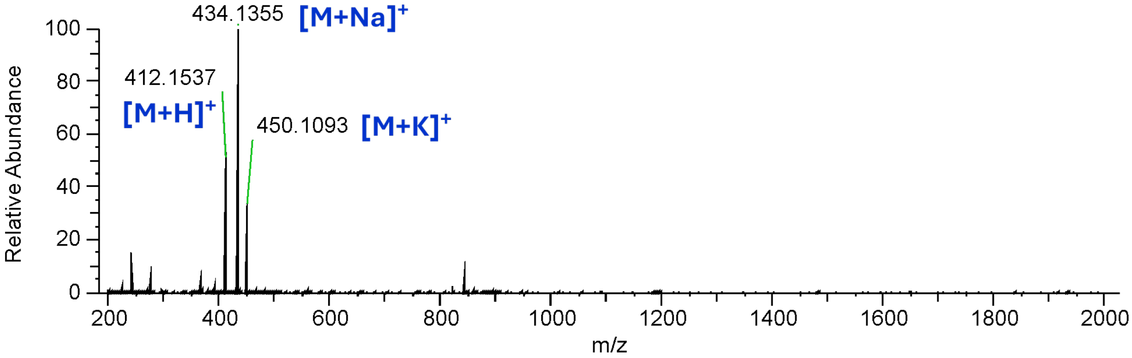

XIII. Supplementary Table 2. Optimization of Thiazole Formation from Thiazoline Dipeptides.

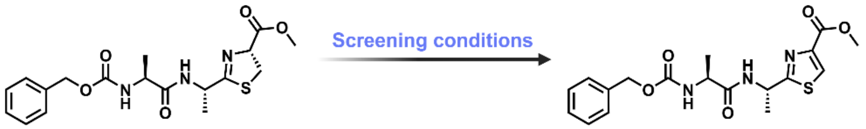

Entry	MnO_2	K_2CO_3	Solvents	Temperature (°C)	Conversion (%)
**1**	10 eq	-	ACN	80	0
**2**	10 eq	3 eq	ACN	80	>99
**3**	10 eq	6 eq	ACN	80	>99
**4**	10 eq	10 eq	ACN	80	>99
**5**	20 eq	20 eq	ACN	80	>99
**6**	-	10 eq	ACN	80	>99

Procedure

The purified thiazoline dipeptide (0.018 mmol, 1 eq) was added to a 1 dram vial equipped with a magnetic stirrer for screening under the conditions listed in Table 2. The reaction was allowed to proceed overnight. The resulting crude mixture was analyzed via **Analytical HPLC Method 1b** to determine the conversion. Under these conditions, the thiazoline dipeptide and the thiazole dipeptide exhibited retention times of 17.3 min and 17.6 min, respectively.

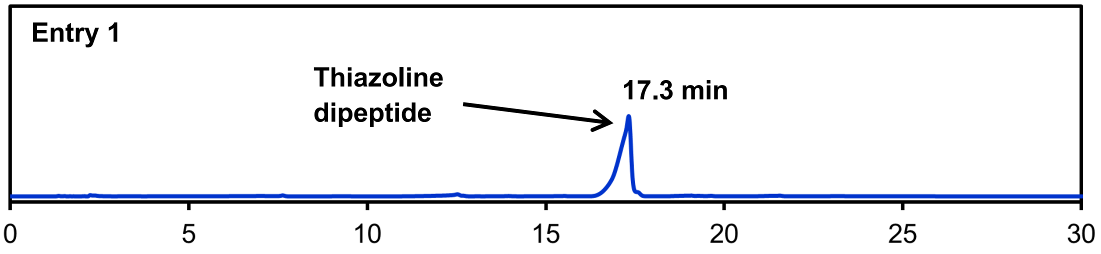

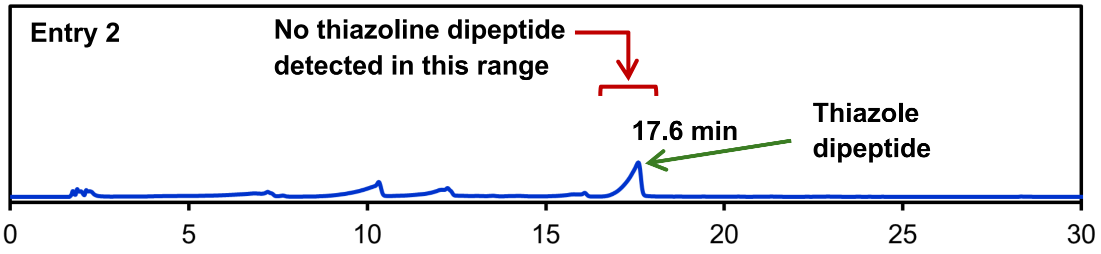

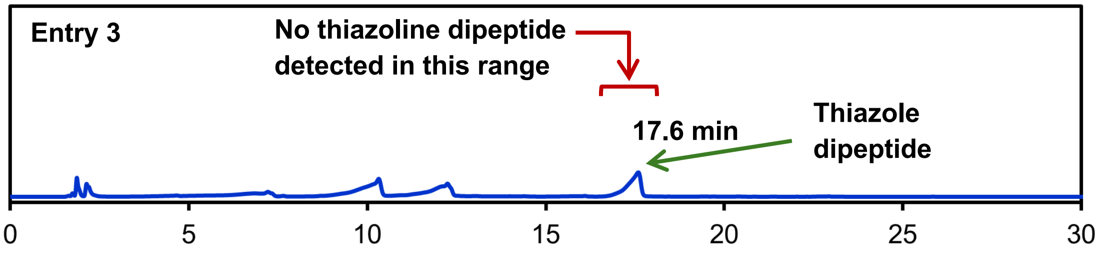

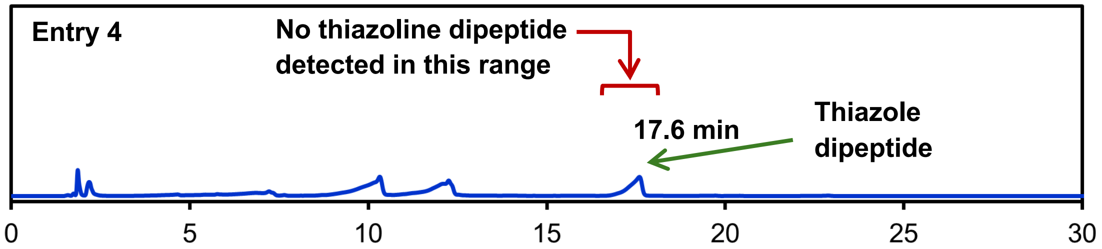
Entry 4
No thiazoline dipeptide detected in this range
17.6 min
Thiazole dipeptide
0
5
10
15
20
25
30

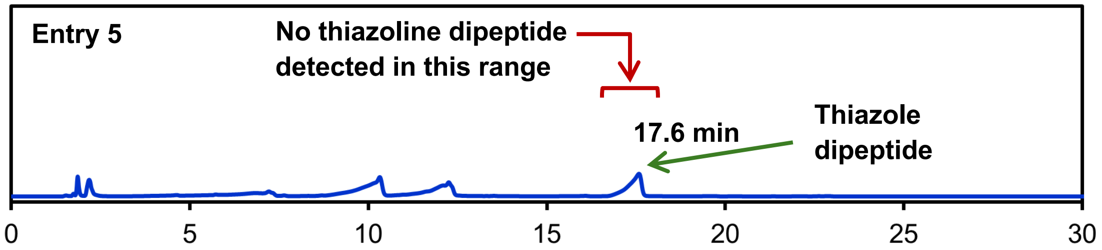
Entry 5
No thiazoline dipeptide detected in this range
17.6 min
Thiazole dipeptide
0
5
10
15
20
25
30

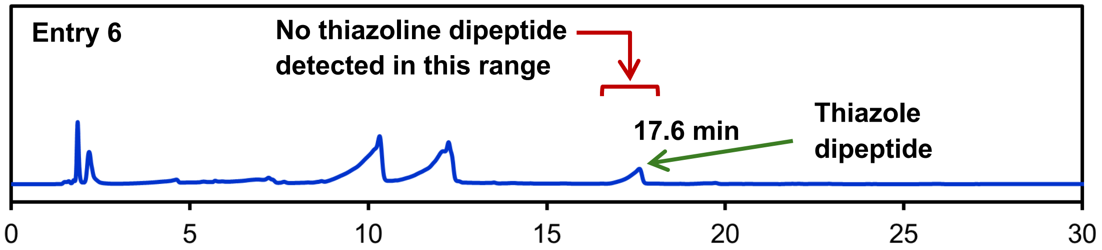
Entry 6
No thiazoline dipeptide detected in this range
17.6 min
Thiazole dipeptide
0
5
10
15
20
25
30

XIV. Supplementary Figure 4. Oxidation of thiazoline dipeptide to thiazole dipeptide.

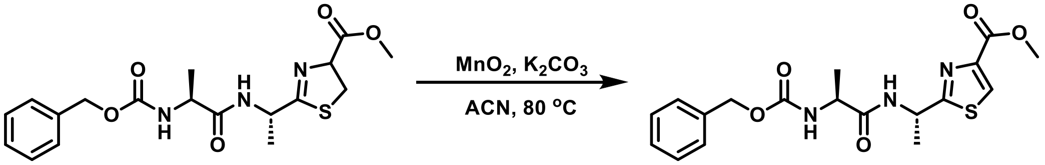

Procedure

To a 1 dram vial equipped with magnetic stirrer were added thiazoline dipeptide (20 mg, 0.05 mmol, 1 eq), ACN (5 mL), MnO_2 (43.5 mg, 0.5 mmol, 10 eq), and K_2O_3 (69.1 mg, 0.5 mmol, 10 eq). The mixture was allowed to react overnight at 80 °C in air. After the reaction was completed, the reaction was allowed to come to room temperature, filtered, and the solvent was removed *in vacuo*. The crude reaction mixture was analyzed via **Analytical HPLC Method 1b** to determine the conversion of the reaction (>99% conversion) and was purified by **HPLC Method A** to obtain a white gel (17.7 mg, 89% isolation yield). **^{1}H NMR** (400 MHz, DMSO) δ 8.82 – 8.73 (m, 1H), 8.43 (d, J = 6.5 Hz, 1H), 7.49 (dd, J = 7.6, 2.8 Hz, 1H), 7.42 – 7.22 (m, 5H), 5.22 – 5.08 (m, 1H), 5.02 (d, J = 3.6 Hz, 2H), 4.09 (dt, J = 10.6, 7.3 Hz, 1H), 3.82 (s, 3H), 1.50 (dd, J = 7.0, 4.9 Hz, 3H), 1.24 (dd, J = 7.1, 5.0 Hz, 3H). **^{13}C NMR** (101 MHz, DMSO) δ 175.5, 172.5, 161.2, 155.7, 145.4, 137.0, 129.0, 128.3, 127.8, 127.7, 65.4, 52.0, 49.9, 46.8, 20.2, 18.0. **HRMS (ESI)** m/z: $[M + H]^+$ Calcd for $C_{18}H_{22}N_3O_5S$ 392.1280, Found 392.1264; $[M + Na]^+$ Calcd for $C_{18}H_{21}N_3O_5SNa$ 414.1100, Found 414.1081; $[M + K]^+$ Calcd for $C_{18}H_{21}N_3O_5SK$ 430.0839, Found 430.0818.

<u>^{1}H NMR spectrum of thiazole dipeptide</u>

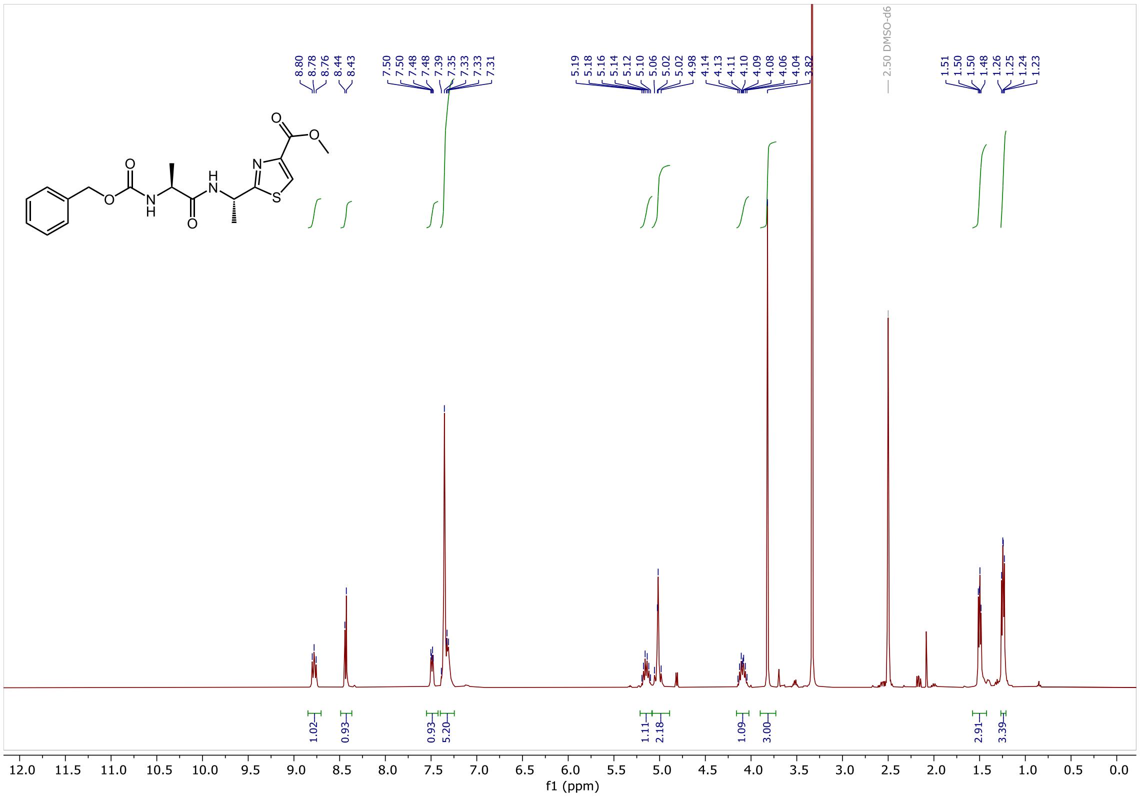

^{13}C NMR spectrum of thiazole dipeptide

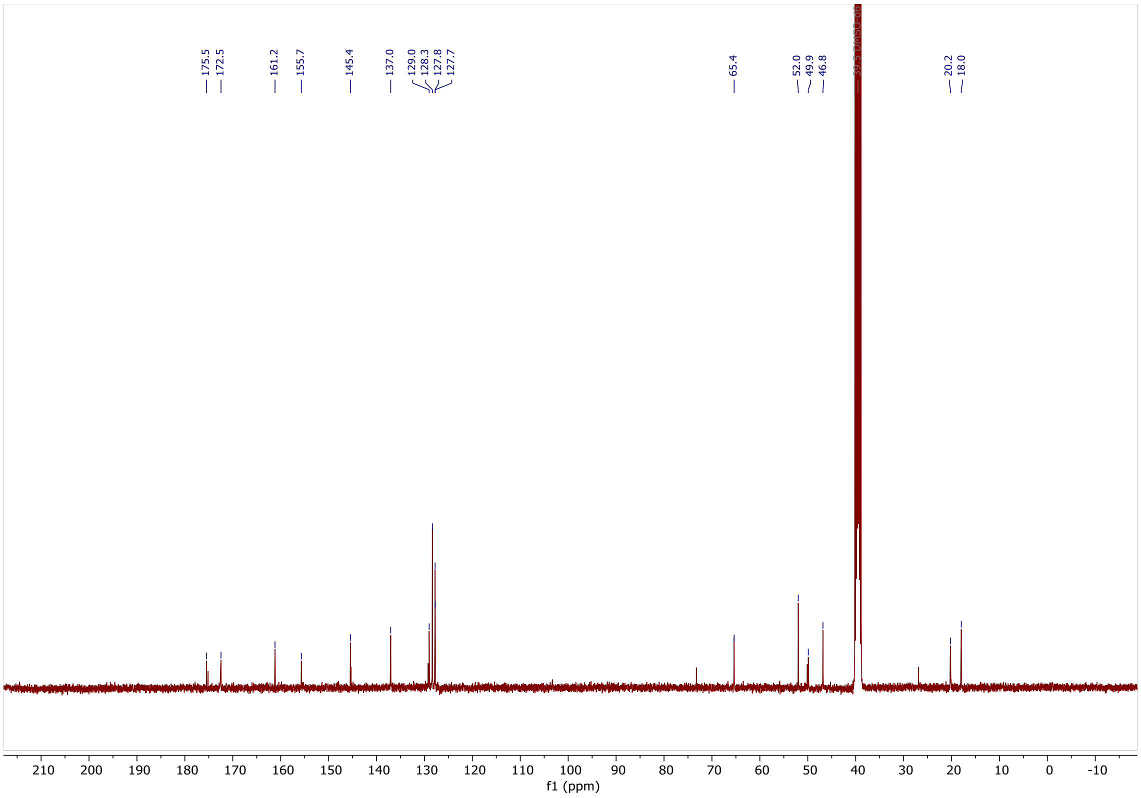

HRMS spectrum of purified thiazole dipeptide

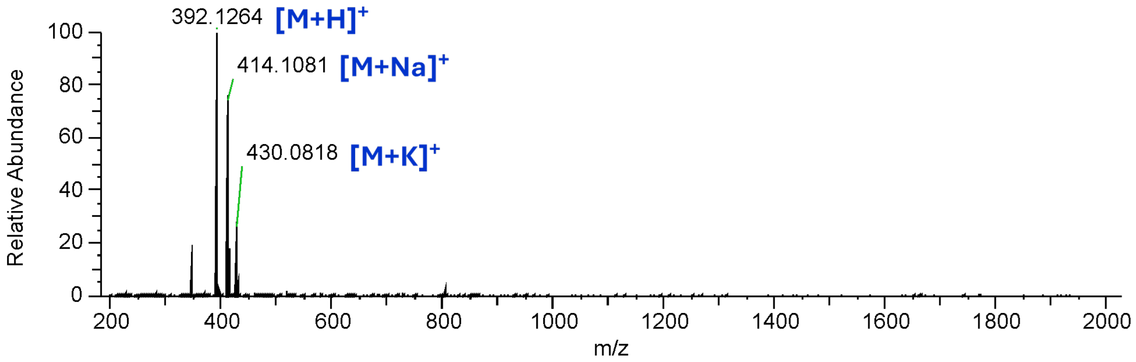

XV. Supplementary Figure 5. Selectivity study of peptide YWRRSCKEHS.

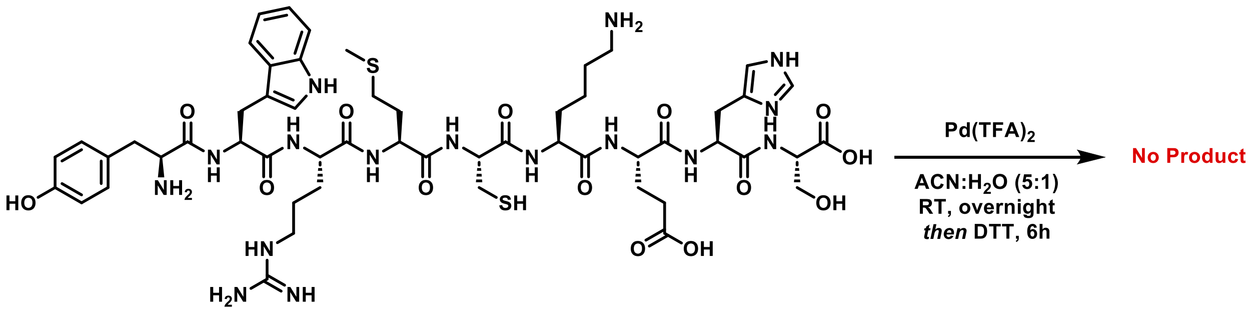

Peptide YWRMCKEHS

Procedure

Peptide YWRMCKEHS (13.8 mg, 0.01 mmol, 1 eq) was subjected to general procedure A and stopped at the end of the nitrile conversion step. Using **Analytical HPLC Method 2a**, no new product was found.

Peptide YWRMCKEHS: **HRMS (ESI)** m/z: $[M + H]^+$ Calcd 1239.5398, Found 1239.5393; $[M + 2H]^{2+}$ Calcd 620.2735, Found 620.2733; $[M + 3H]^{3+}$ Calcd 413.8514, Found 413.8514. **Analytical HPLC Method 2a**: retention time of 9.1 min.
Fraction at 9.1 min from the crude reaction mixture: HRMS (ESI) m/z: $[M + H]^+$ Found 1239.5391; $[M + 2H]^{2+}$ Found 620.2734; $[M + 3H]^{3+}$ Found 413.8514.

Analytical HPLC trace of the purified peptide YWRMCKEHS

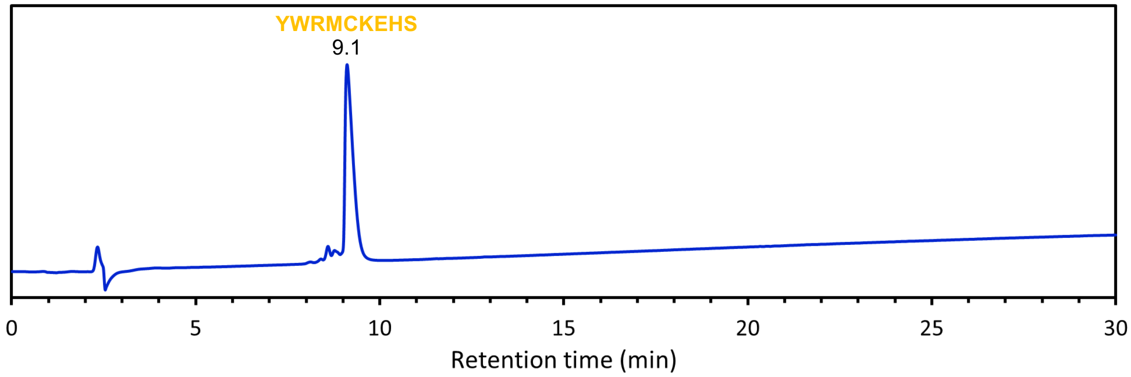

HRMS spectrum of the purified peptide YWRMCKEHS

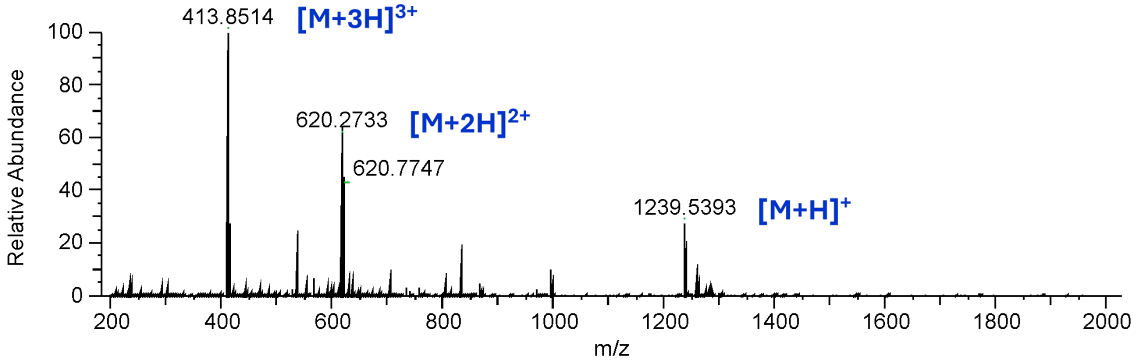

Analytical HPLC trace of the crude reaction mixture of peptide YWRMCKEHS

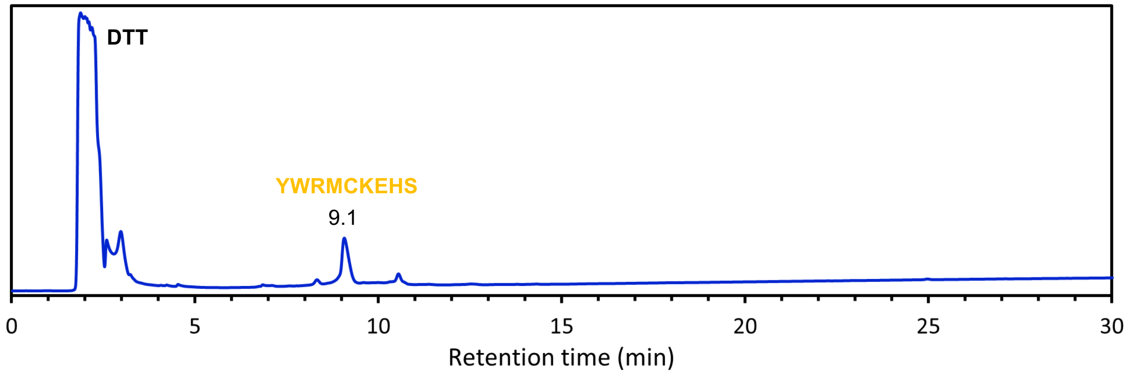

HRMS spectrum of fraction 9.1 min confirming the selectivity of peptide YWRMCKEHS

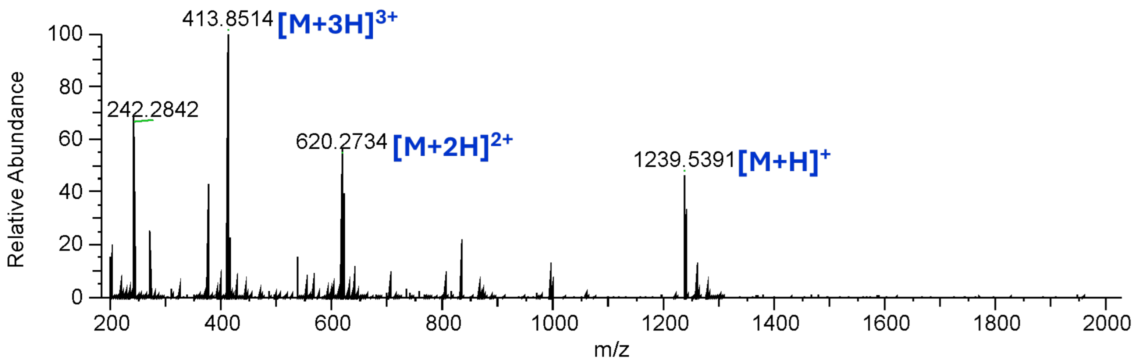

XVI. Supplementary Figure 6. Selectivity study converting peptide CWPAYA-Amide to peptide CWPAYA-Nitrile and peptide CWPAYA-Thiazoline.

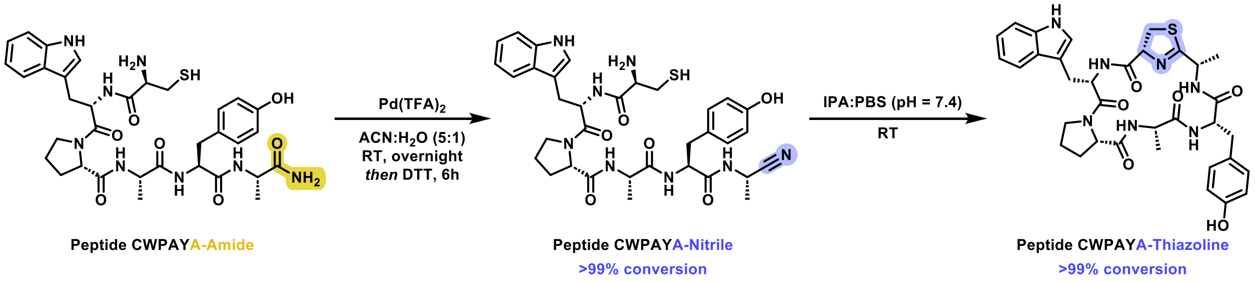

Procedure

The peptide CWPAYA-Amide (7.1 mg, 0.01 mmol, 1 eq) was subjected to general procedure A to afford the nitrile peptide CWPAYA-Nitrile (>99% conversion). The purified nitrile peptide was then subjected to cyclization according to procedure A, yielding the peptide CWPAYA-Thiazoline (>99% conversion).

Peptide CWPAYA-Amide: **HRMS (ESI)** m/z: $[M + H]^+$ Calcd 709.3132, Found 709.3130; $[M + Na]^+$ Calcd 731.2952, Found 731.2946. **Analytical HPLC Method 2a**: retention time of 12.3 min.
Peptide CWPAYA-Nitrile: **HRMS (ESI)** m/z: $[M + H]^+$ Calcd 691.3026, Found 691.3023; $[M + Na]^+$ Calcd 713.2846, Found 713.2841; $[M + K]^+$ Calcd 729.2585, Found 729.2578. **Analytical HPLC Method 2a**: retention time of 15.1 min.

Peptide CWPAYA-Thiazoline: **HRMS (ESI)** m/z: $[M + H]^+$ Calcd 674.2761, Found 674.2754; $[M + Na]^+$ Calcd 696.2581, Found 696.2571. **Analytical HPLC Method 2b**: retention time of 17.8 min.

Analytical HPLC trace of purified peptide CWPAYA-Amide

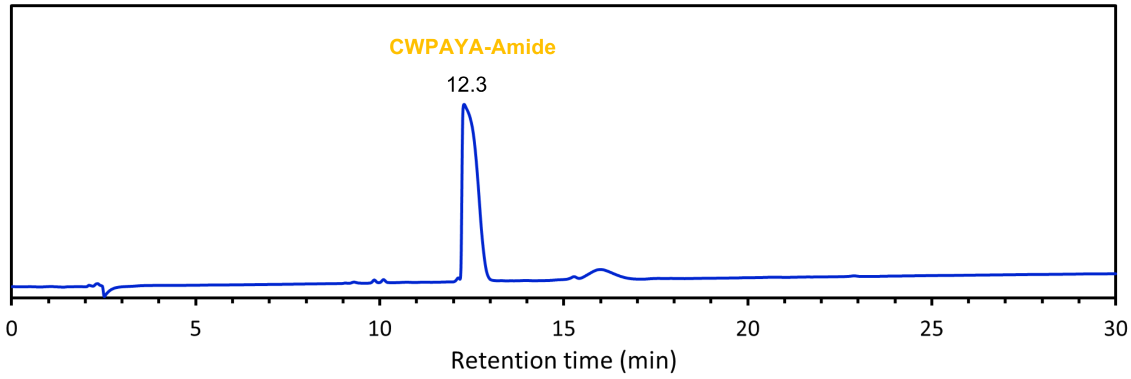

HRMS spectrum of purified peptide CWPAYA-Amide

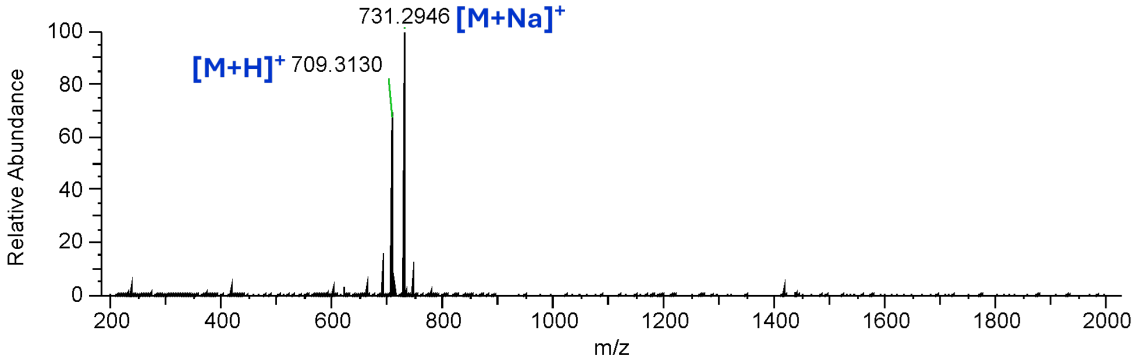

Analytical HPLC trace of the crude reaction mixture converting CWPAYA-Amide to CWPAYA-Nitrile

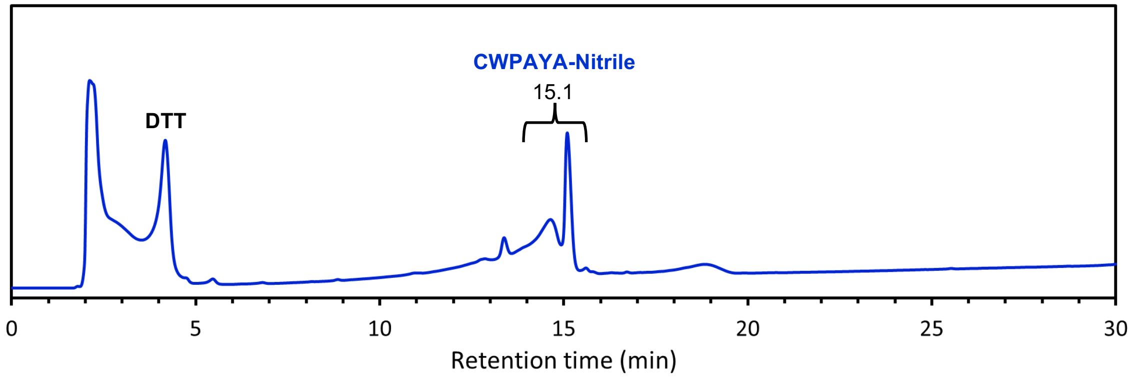

HRMS spectrum of fraction 15.1 min confirming the formation of CWPAYA-Nitrile

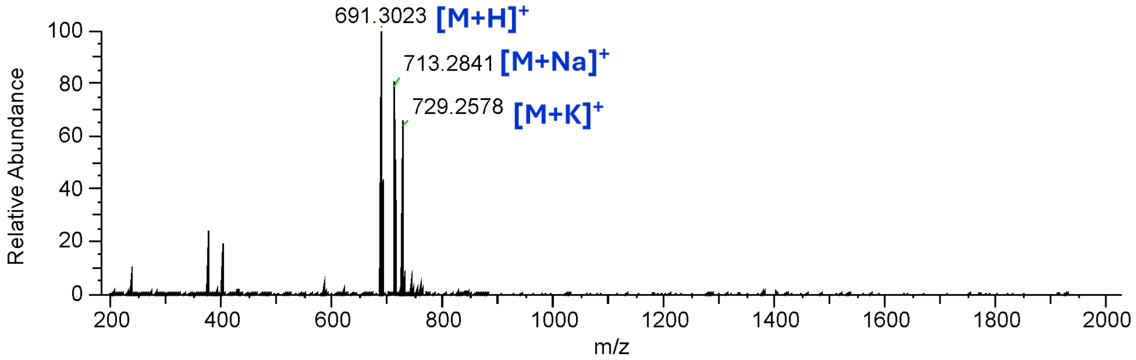

Analytical HPLC trace of the reaction mixture converting CWPAYA-Nitrile to CWPAYA-Thiazoline

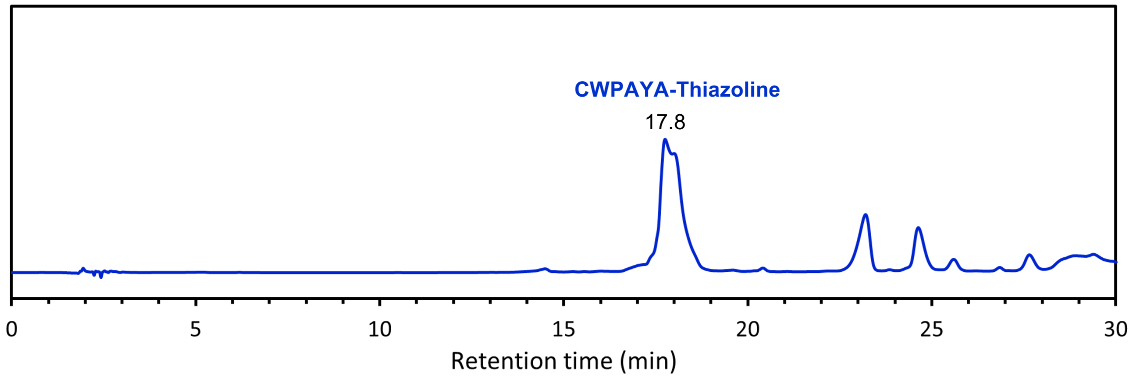

HRMS spectrum of fraction 17.8 min confirming the formation of CWPAYA-Thiazoline

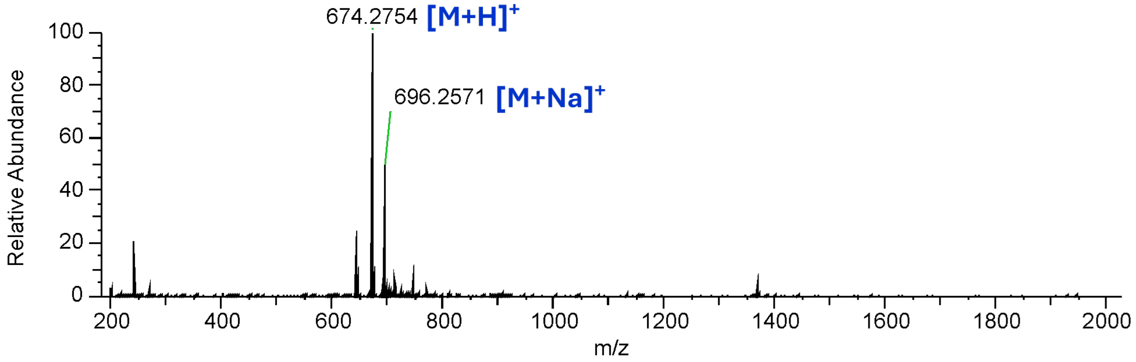

XVII. Supplementary Figure 7. Selectivity study on the conversion of peptide CWPAYQ-COOH to peptide CWPAYQ-Nitrile-COOH and the attempted formation of CWPAYQ-Thiazoline-COOH.

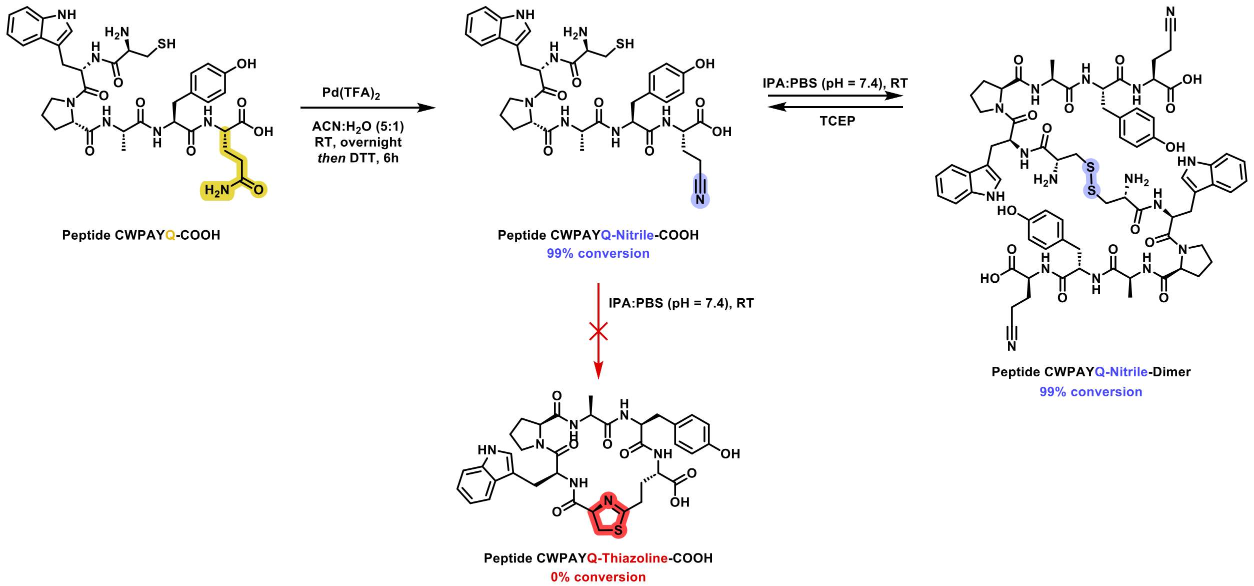

Procedure

The peptide CWPAYQ-COOH (7.6 mg, 0.01 mmol, 1 eq) was subjected to general procedure A to afford the nitrile peptide CWPAYQ-Nitrile (>99% conversion). The purified nitrile peptide was then subjected to cyclization according to procedure A, but no thiazoline peptide CWPAYQ-Thiazoline was observed. Instead, the dimer peptide CWPAYQ-Nitrile-Dimer was formed. Subsequent treatment with TCEP facilitated the conversion of the dimer back to the nitrile peptide CWPAYQ-Nitrile within 10 min.

Peptide CWPAYQ-COOH: **HRMS (ESI)** m/z: $[M + H]^+$ Calcd 767.3187, Found 767.3000; $[M + Na]^+$ Calcd 789.3006, Found 789.2811. **Analytical HPLC Method 2a**: retention time of 12.4 min.
Peptide CWPAYQ-Nitrile: HRMS (ESI) m/z: $[M + H]^+$ Calcd 749.3081, Found 749.3075; $[M + Na]^+$ Calcd 771.2901, Found 771.2892. **Analytical HPLC Method 2a**: retention time of 13.2 min and 13.5 min.
Peptide CWPAYQ-Nitrile-Dimer: HRMS (ESI) m/z: $[M + H]^+$ Calcd 1495.5927, Found 1495.5914; $[M + 2H]^{2+}$ Calcd 748.3003, Found 748.2997. **Analytical HPLC Method 2b**: retention time of 18.1 min.

Analytical HPLC trace of the purified peptide CWPAYQ-COOH

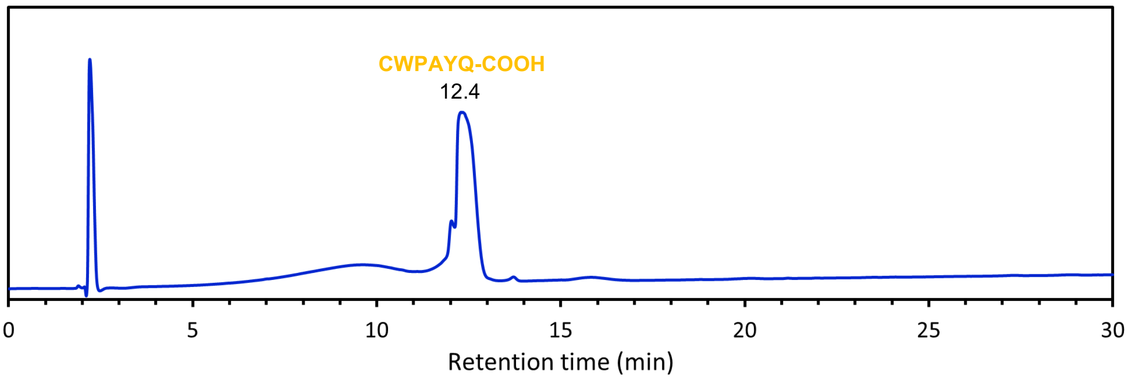

HRMS spectrum of purified peptide CWPAYQ-COOH

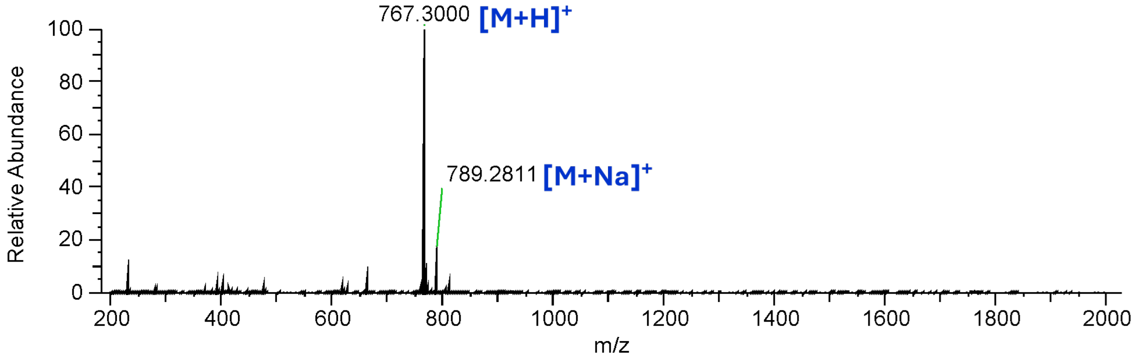

Analytical HPLC trace of the crude reaction mixture converting CWPAYQ-COOH to CWPAYQ-Nitrile

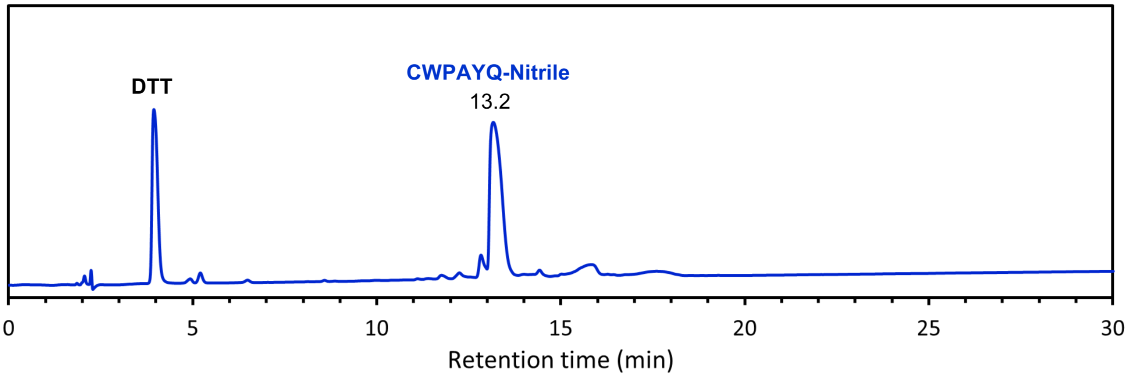

HRMS spectrum of fraction 13.2 min confirming the formation of CWPAYQ-Nitrile

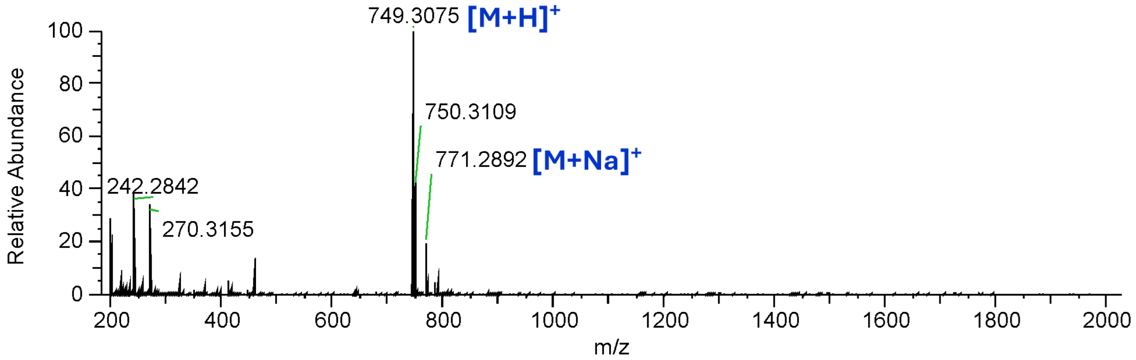

Analytical HPLC trace of the crude reaction mixture converting CWPAYQ-Nitrile to CWPAYQ-Nitrile-Dimer

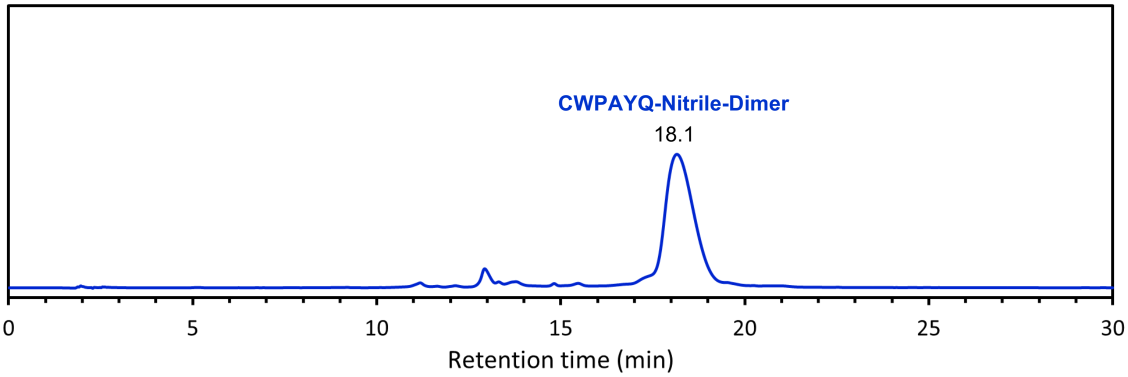

HRMS spectrum of fraction 18.1 min confirming the formation of CWPAYQ-Nitrile-Dimer

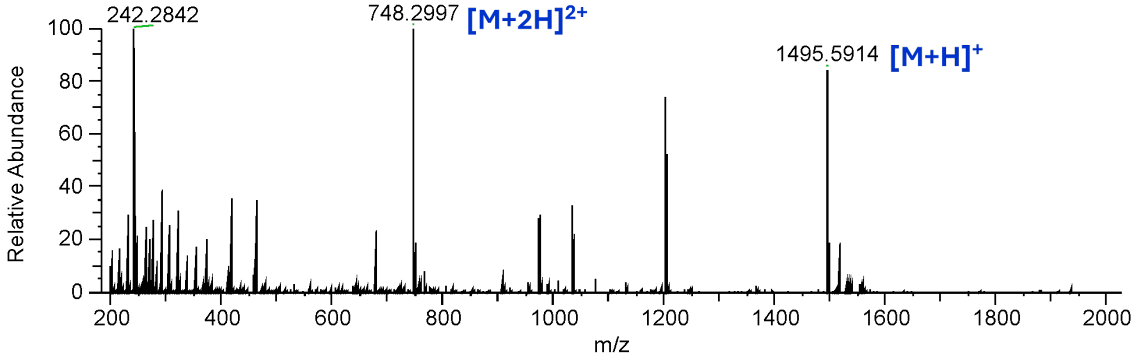

Analytical HPLC trace of the crude reaction mixture of CWPAYQ-Nitrile-Dimer after adding TCEP for 10 min

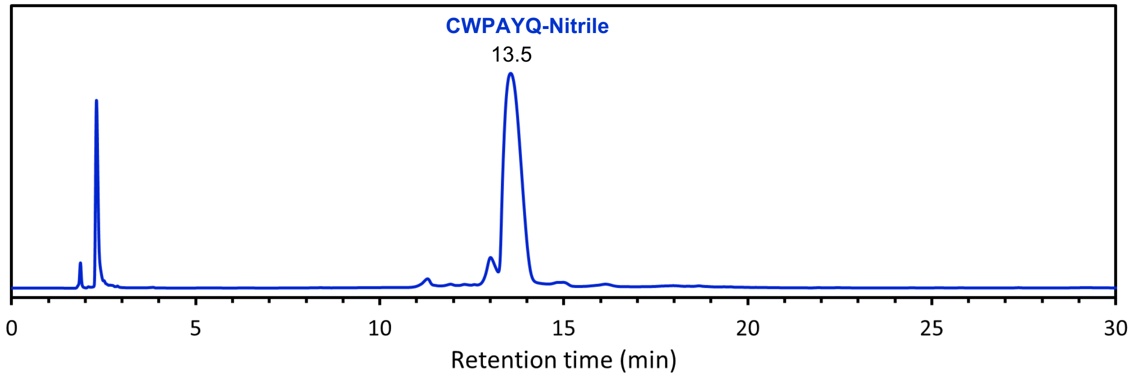

HRMS spectrum of fraction 13.5 min confirming the reformation of CWPAYQ-Nitrile

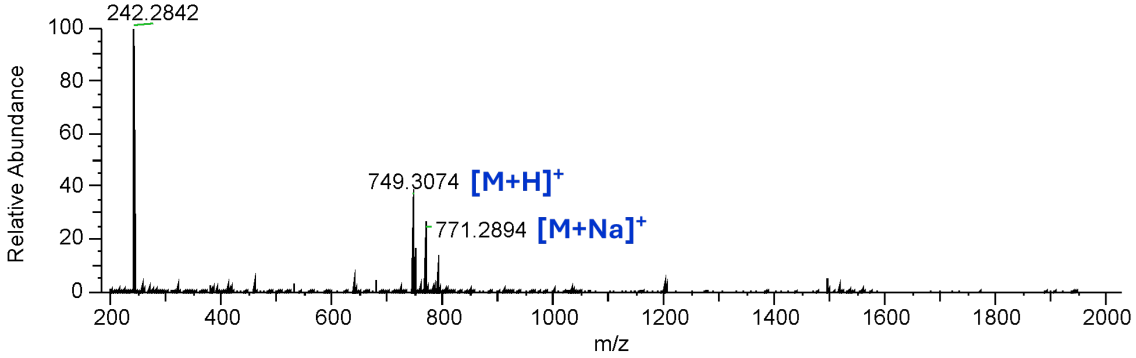

XVIII. Supplementary Figure 8. Study on the conversion of peptide WGNFL-COOH to peptide WGN-Nitrile-FL-COOH and the reformation of WGNFL-COOH.

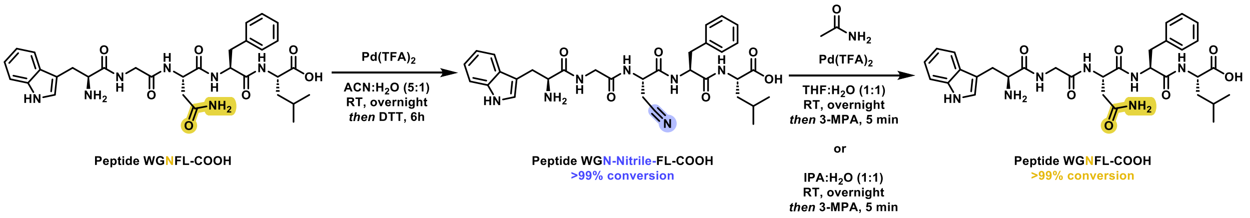

Procedure

The peptide WGNFL-COOH (6.3 mg, 0.01 mmol, 1 eq) was subjected to general procedure A to afford the nitrile peptide WGN-Nitrile-FL-COOH (>99% conversion). Then, in a 1 dram vial equipped with a magnetic stirrer was added the purified WGN-Nitrile-FL-COOH (1 mg, 0.00163 mmol, 1 eq), palladium(II) trifluoroacetate ($Pd(TFA)_2$) (1.62 mg, 0.00489 mmol, 3 eq), acetamide (3.85 mg, 0.0652 mmol, 40 eq), H_2O (500 µL), and THF (500 µL) or IPA (500 µL) as co-solvent. The resulting mixture was allowed to react overnight at room temperature. Subsequently, 3-Mercaptopropionic acid (3-MPA) (4.26 µL, 0.0489 mmol, 30 eq) was introduced to the reaction mixture and stirred for 5 minutes. After filtration to remove the yellow precipitate, a clear, colorless solution containing the amide peptide was obtained (>99% conversion).

Peptide WGNFL-COOH: **HRMS (ESI)** m/z: $[M + H]^+$ Calcd 636.3140, Found 636.3155. **Analytical HPLC Method 1a**: retention time of 12 min.

Peptide WGN-Nitrile-FL-COOH: HRMS (ESI) m/z: $[M + H]^+$ Calcd 618.3035, Found 618.3045. **Analytical HPLC Method 1a**: retention time of 12.8 min.

Analytical HPLC trace of the purified peptide WGNFL-COOH

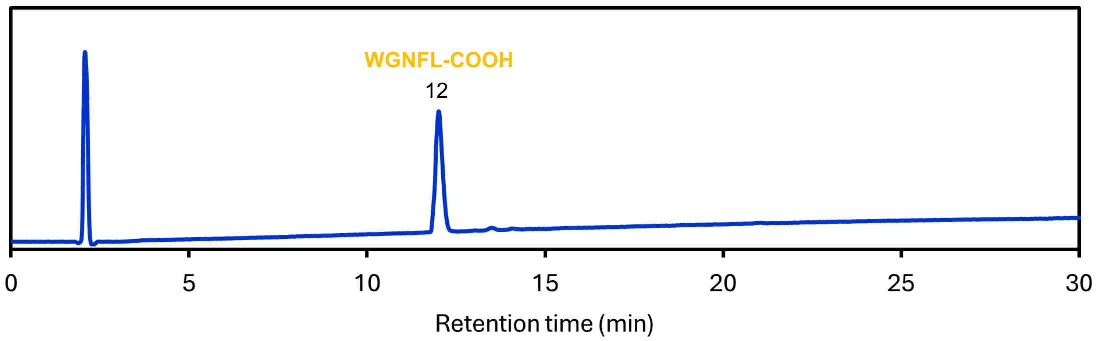

HRMS spectrum of purified peptide WGNFL-COOH

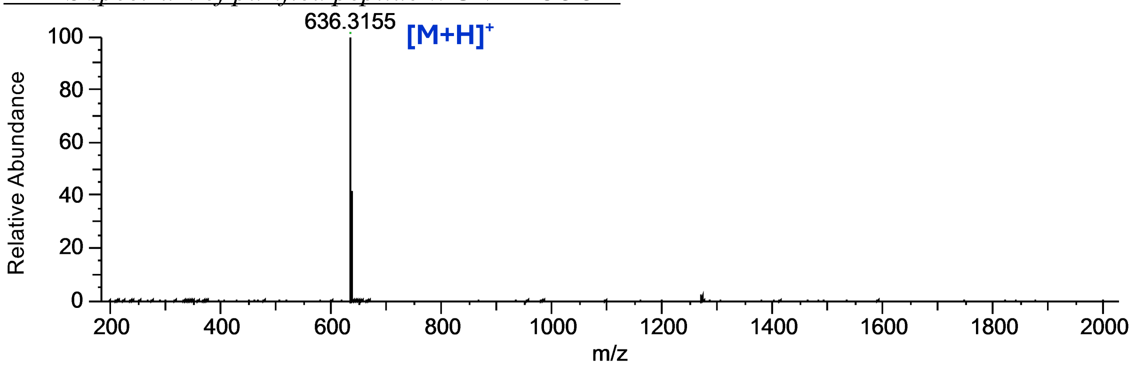

Analytical HPLC trace of crude reaction mixture converting WGNFL-COOH to WGN-Nitrile-FL-COOH

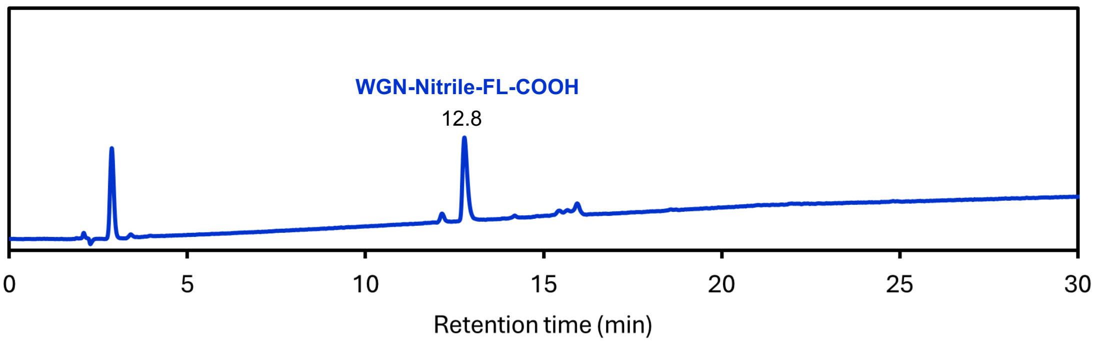

HRMS spectrum of fraction 12.8 min confirming the formation of WGN-Nitrile-FL-COOH

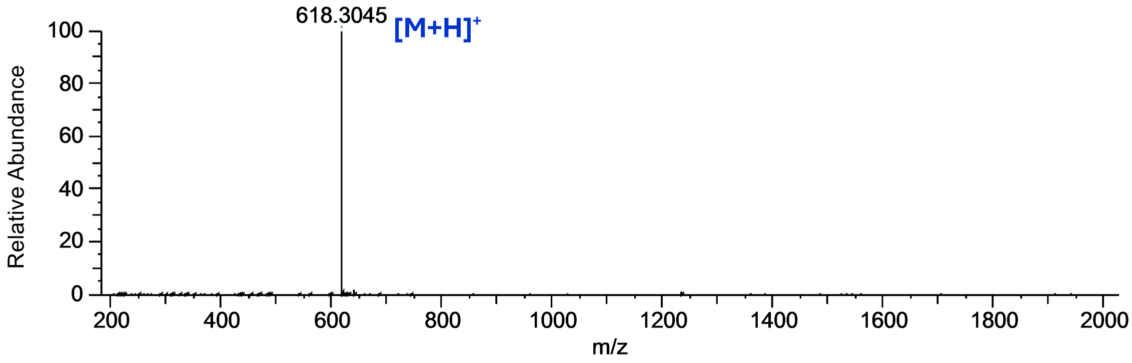

Analytical HPLC trace of crude reaction mixture converting WGN-Nitrile-FL-COOH back to WGNFL-COOH (H_2O:THF as solvent)

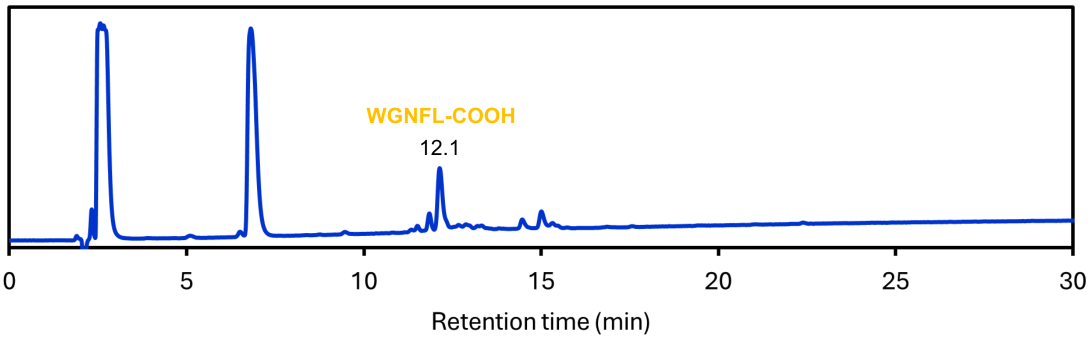

Analytical HPLC trace of crude reaction mixture converting WGN-Nitrile-FL-COOH back to WGNFL-COOH (H_2O:IPA as solvent)

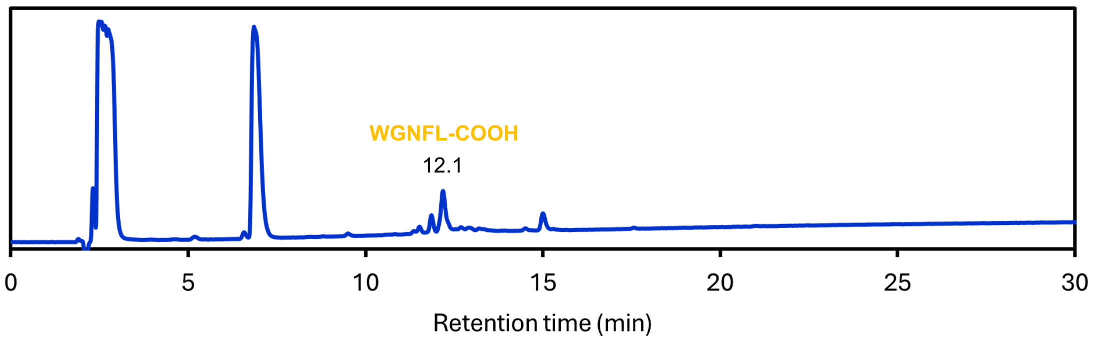

HRMS spectrum of fraction 12.1 min confirming the reformation of WGNFL-COOH

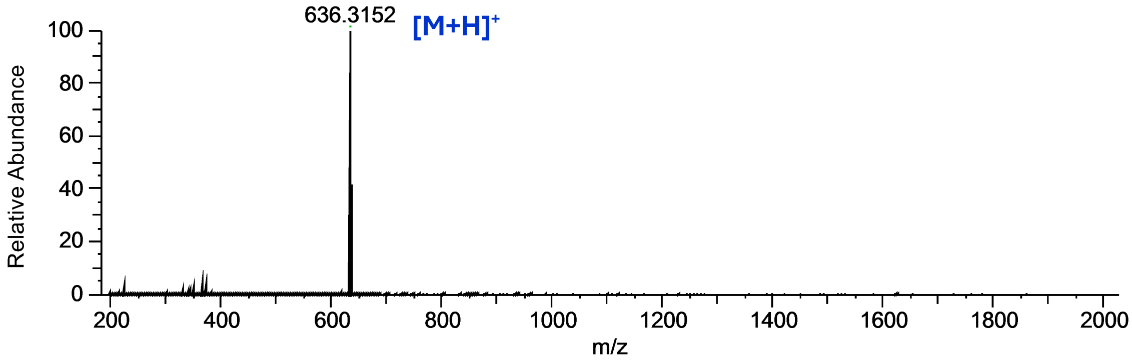

XIX. Supplementary Table 3. Scope of C-terminal nitrile formation across diverse C-terminal amino acids with unprotected reactive side chains.

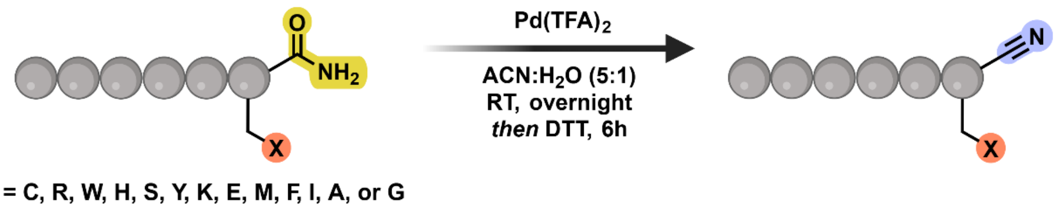

Entry	Peptide	Conversion (%)
1	Ac-RFGKGFC-Amide	47
2	Ac-MWAFR-Amide	86
3	H_2N-FGGGW-Amide	95
4	H_2N-GGFH-Amide	63
5	H_2N-FM(ox)FGS-Amide	67
6	Ac-FKAPAPRY-Amide	61
7	Ac-WAFRK-Amide	82
8	H_2N-CIVPPE-Amide	68
9	H_2N-CAFPIPARM-Amide	>99
10	H_2N-CIVPPF-Amide	>99
11	H_2N-CAFPIPI-Amide	>99
12	H_2N-CAFPIPA-Amide	>99
13	H_2N-CPHVF-Amide	>99
14	H_2N-CMPPMPF-Amide	>99
15	H_2N-CTLWPFG-Amide	>99

Procedure

The following peptides (0.01 mmol, 1 eq) were subjected to general procedure A, and the reactions were terminated after the nitrile conversion step, except for entries 8-13. **Analytical HPLC Method 1a** was used to determine the conversion from the C-terminal amide to the corresponding C-terminal nitrile. For the peptide GGFH-Amide, **Analytical HPLC Method 3a** was used to determine this conversion. Entries 10-15 will be shown in the following sections XX-XXV (Figure 9-14).

Entry 1

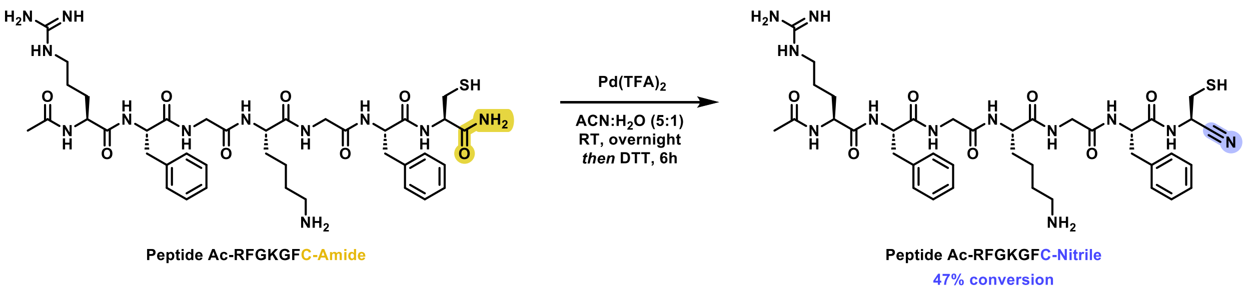

Peptide Ac-RFGKGFC-Amide: **HRMS (ESI)** m/z: $[M + H]^+$ Calcd 855.4294, Found 855.4299; $[M + 2H]^{2+}$ Calcd 428.2184, Found 428.2197. **Analytical HPLC Method 1a**: retention time of 9.3 min.

Peptide Ac-RFGKGFC-Nitrile: HRMS (ESI) m/z: $[M + H]^+$ Calcd 837.4188, Found 837.4192; $[M + 2H]^{2+}$ Calcd 419.2131, Found 419.2142. **Analytical HPLC Method 1a**: retention time of 11 min. Conversion: 47%.

Analytical HPLC trace of the purified peptide Ac-RFGKGFC-Amide

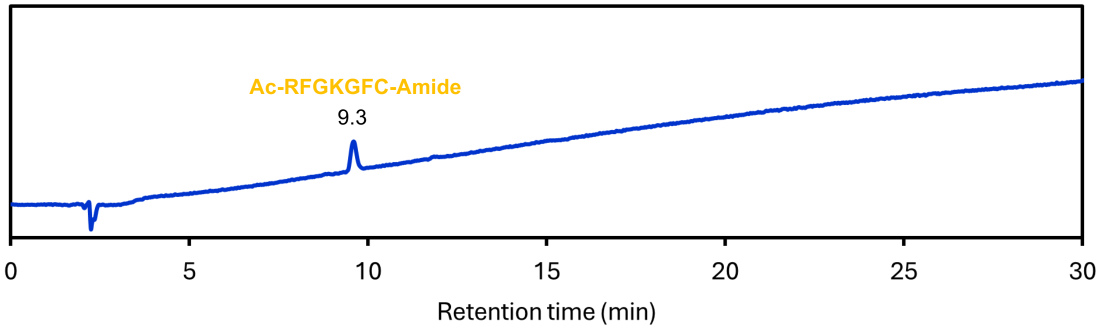

HRMS spectrum of the purified peptide Ac-RFGKGFC-Amide

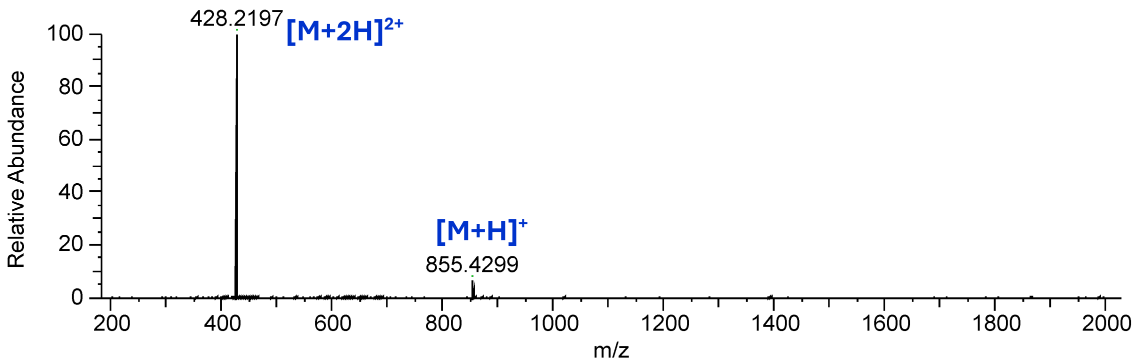

Analytical HPLC trace of the crude reaction converting Ac-RFGKGFC-Amide to Ac-RFGKGFC-Nitrile

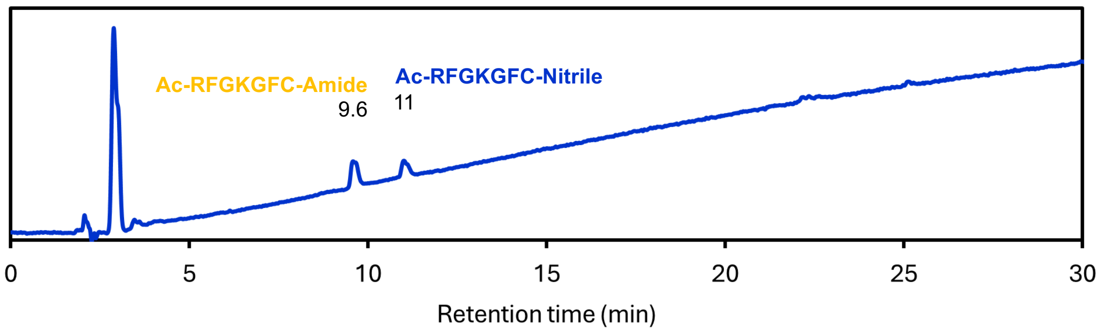

HRMS spectrum of fraction 11 min confirming the formation of Ac-RFGKGFC-Nitrile

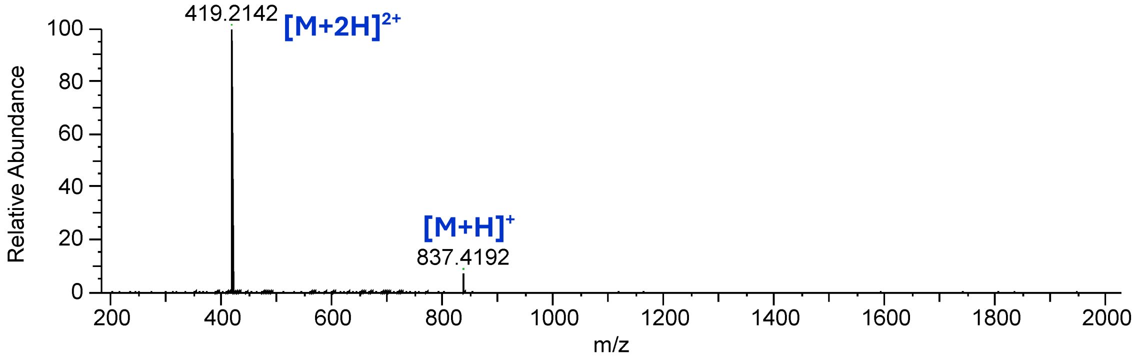

Entry 2

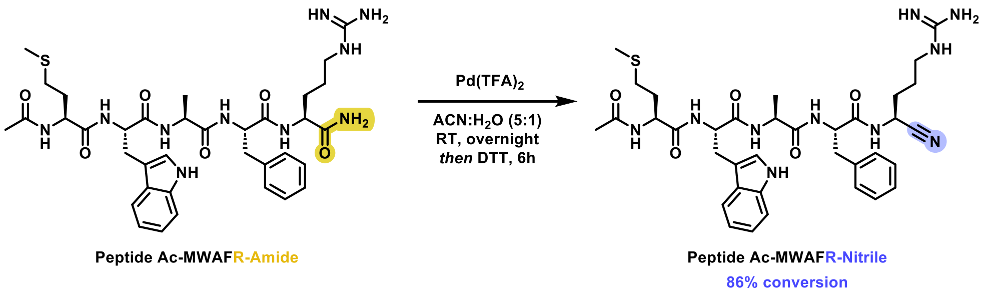

Peptide Ac-MWAFR-Amide: **HRMS (ESI)** m/z: $[M + H]^+$ Calcd 751.3708, Found 751.3713. **Analytical HPLC Method 1a**: retention time of 13.6 min.

Peptide Ac-MWAFR-Nitrile: HRMS (ESI) m/z: $[M + H]^+$ Calcd 733.3603, Found 733.3611. **Analytical HPLC Method 1a**: retention time of 14.5 min. Conversion: 86%.

Analytical HPLC trace of the purified peptide Ac-MWAFR-Amide

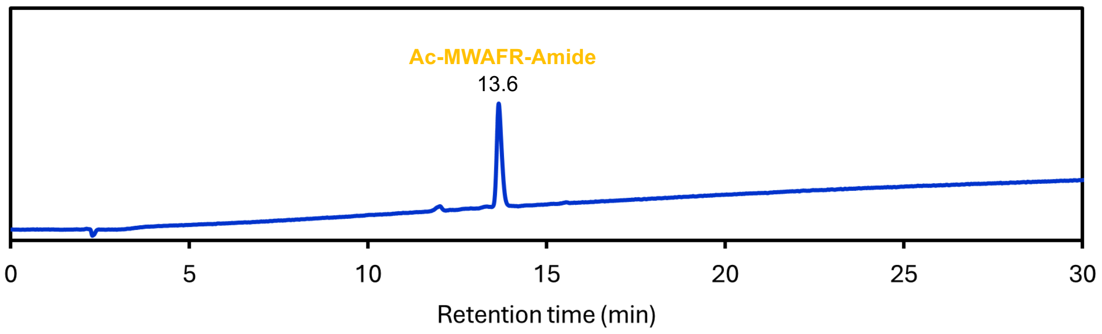

HRMS spectrum of the purified peptide Ac-MWAFR-Amide

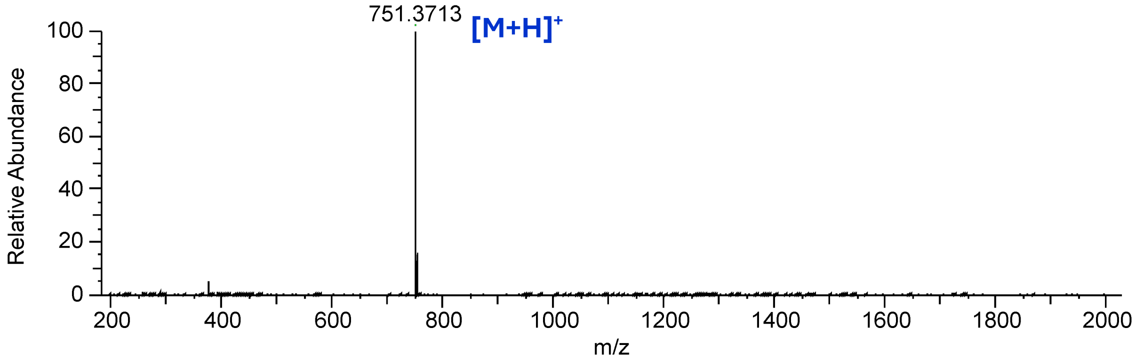

Analytical HPLC trace of the crude reaction converting Ac-MWAFR-Amide to Ac-MWAFR-Nitrile

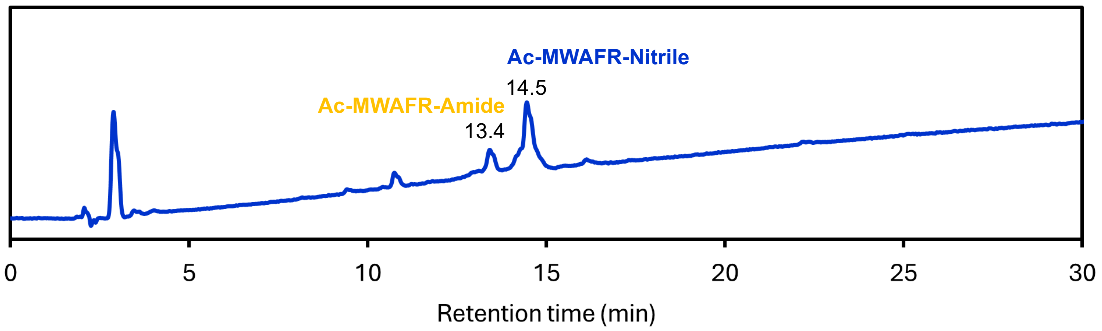

HRMS spectrum of fraction 14.5 min confirming the formation of Ac-MWAFR-Nitrile

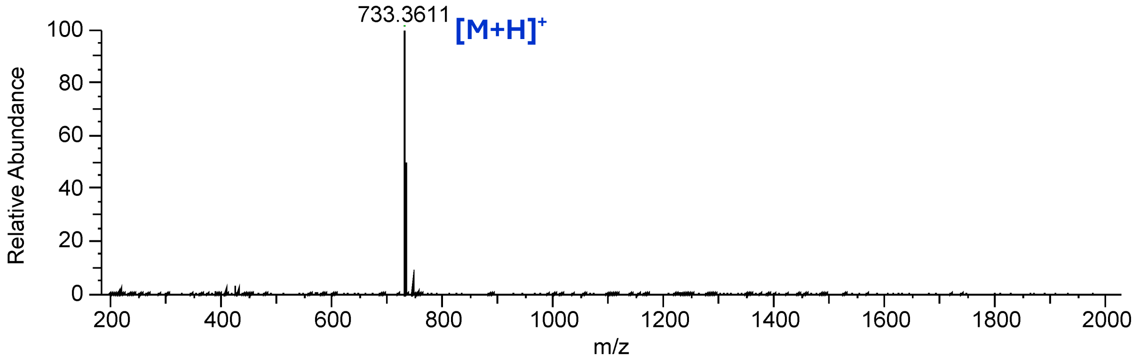

Entry 3

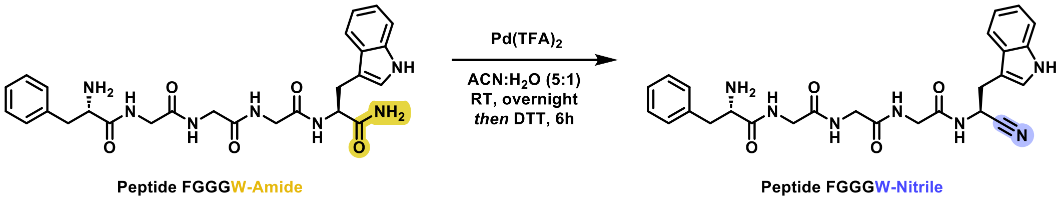

Peptide FGGGW-Amide: **HRMS (ESI)** m/z: $[M + H]^+$ Calcd 522.2459, Found 522.2466. **Analytical HPLC Method 1a**: retention time of 8.8 min.

Peptide FGGGW-Nitrile: HRMS (ESI) m/z: $[M + H]^+$ Calcd 504.2354, Found 504.2361. **Analytical HPLC Method 1a**: retention time of 10.8 min. Conversion: 95%.

Analytical HPLC trace of the purified peptide FGGGW-Amide

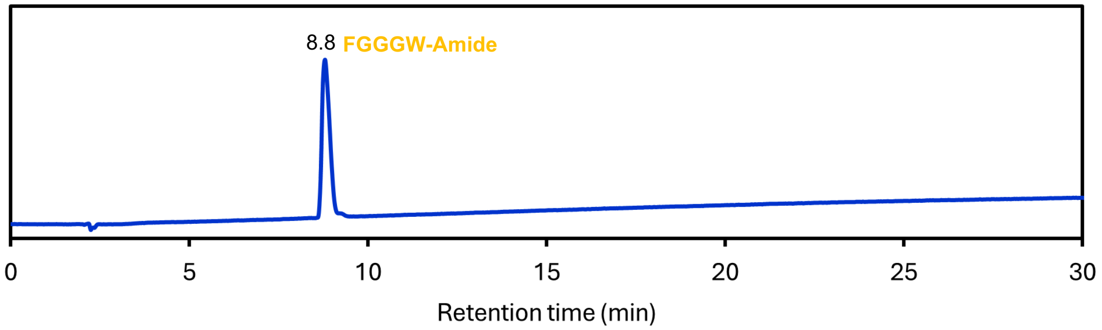

HRMS spectrum of the purified peptide FGGGW-Amide

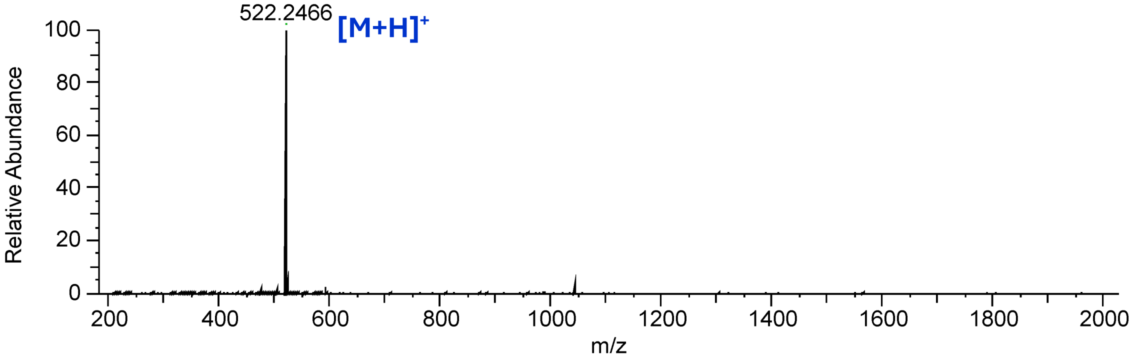

Analytical HPLC trace of the crude reaction converting FGGGW-Amide to FGGGW-Nitrile

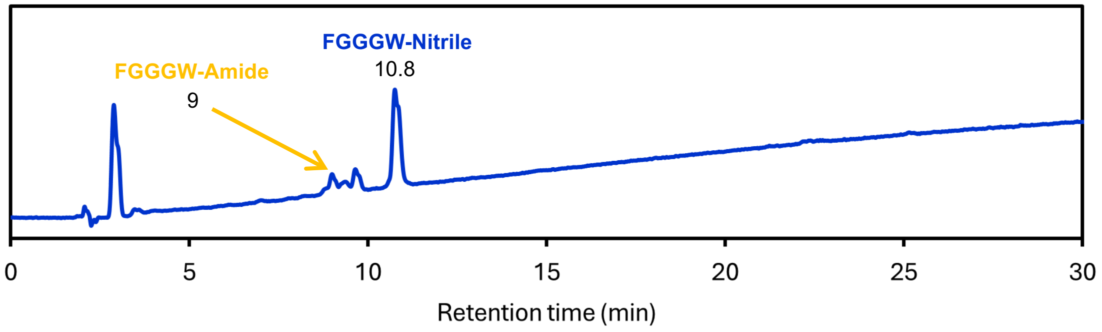

HRMS spectrum of fraction 10.8 min confirming the formation of FGGGW-Nitrile

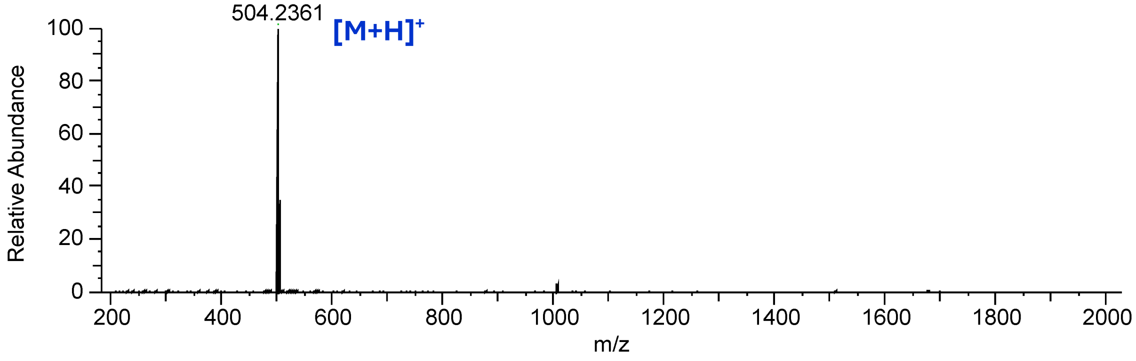

Entry 4

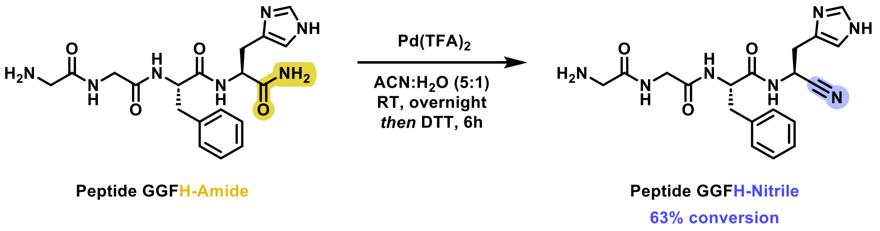

Peptide GGFH-Amide: **HRMS (ESI)** m/z: $[M + H]^+$ Calcd 416.2041, Found 416.2044. **Analytical HPLC Method 3a**: retention time of 3.4 min.

Peptide GGFH-Nitrile: HRMS (ESI) m/z: $[M + H]^+$ Calcd 398.1935, Found 398.1938. **Analytical HPLC Method 3a**: retention time of 4.6 min. Conversion: 63%.

Analytical HPLC trace of the purified peptide GGFH-Amide

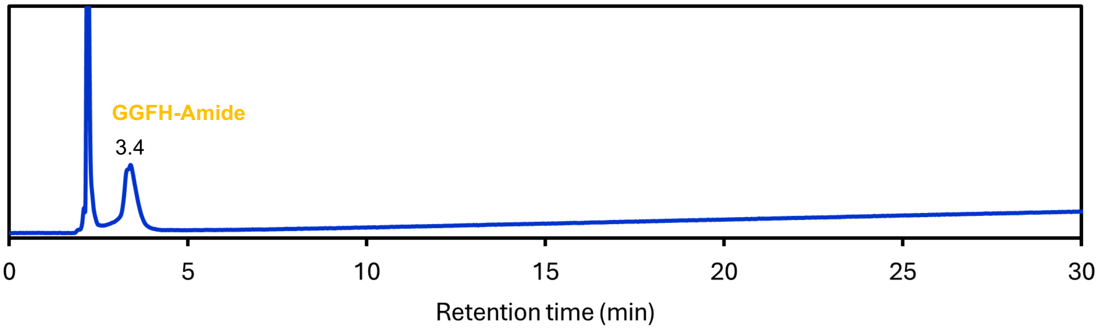

HRMS spectrum of the purified peptide GGFH-Amide

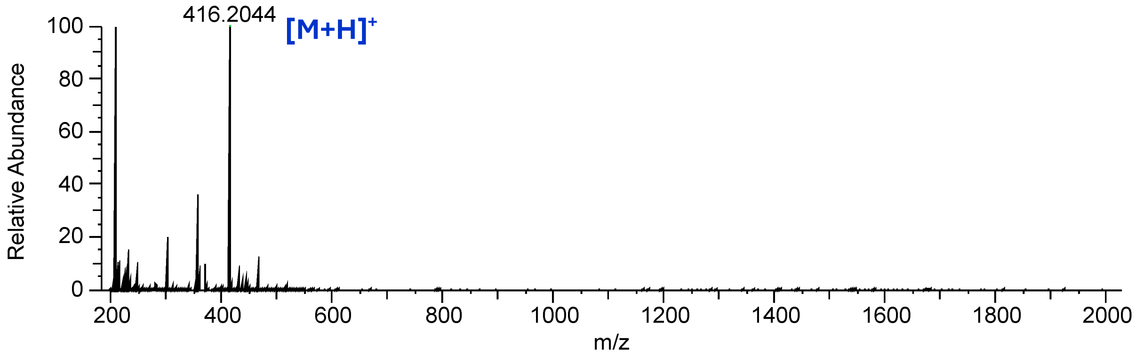

Analytical HPLC trace of the crude reaction converting GGFH-Amide to GGFH-Nitrile

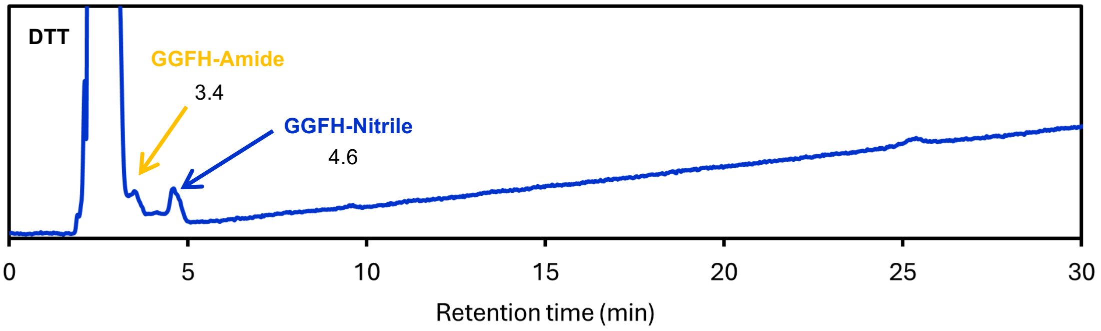

HRMS spectrum of fraction 4.6 min confirming the formation of GGFH-Nitrile

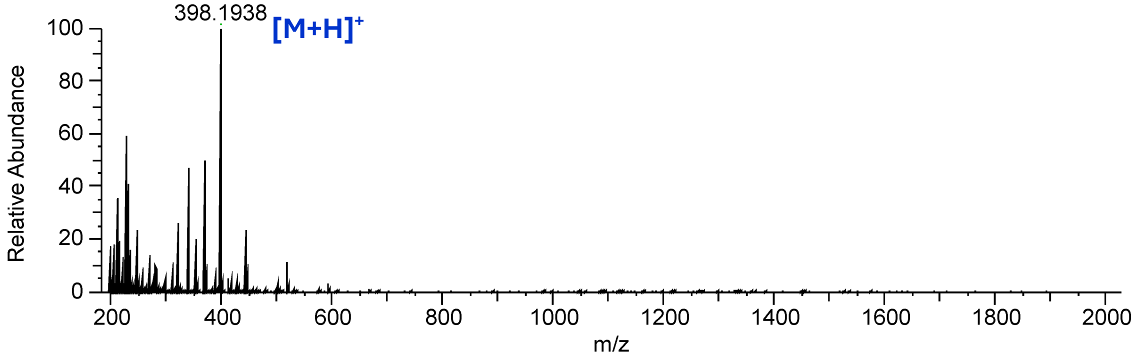

Entry 5

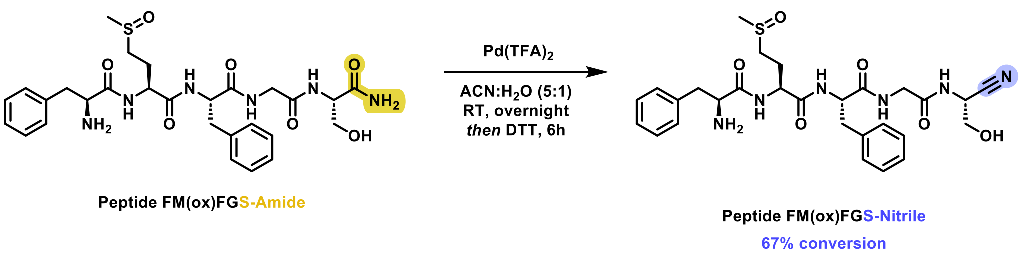

Peptide FM(ox)FGS-Amide: **HRMS (ESI)** m/z: $[M + H]^+$ Calcd 603.2595, Found 603.2603. **Analytical HPLC Method 1a**: retention time of 9.2 min.

Peptide FM(ox)FGS-Nitrile: HRMS (ESI) m/z: $[M + H]^+$ Calcd 585.2490, Found 585.2498. **Analytical HPLC Method 1a**: retention time of 9.8 min. Conversion: 67%.

Analytical HPLC trace of the purified peptide FM(ox)FGS-Amide

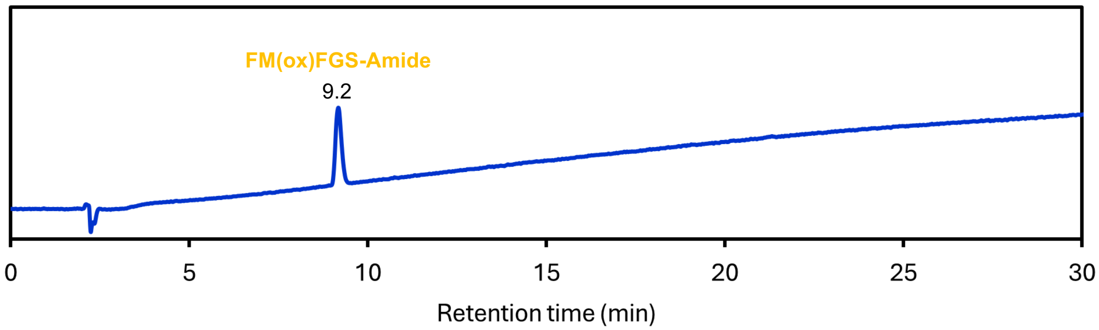

HRMS spectrum of the purified peptide FM(ox)FGS-Amide

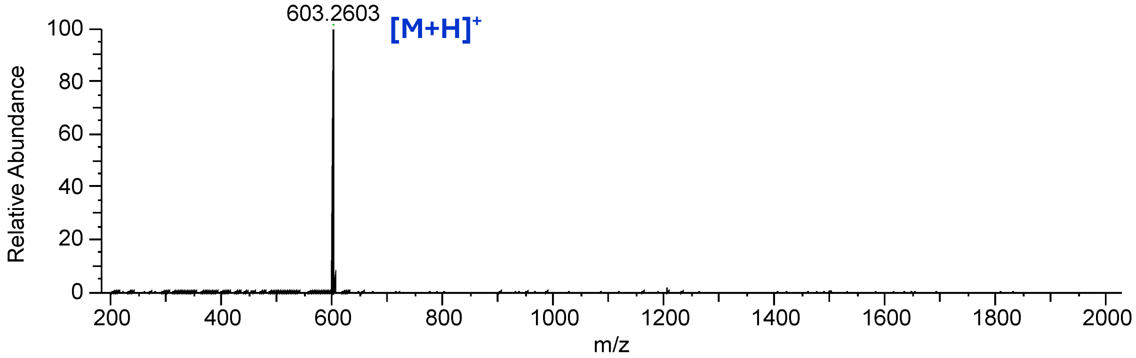

Analytical HPLC trace of the crude reaction converting FM(ox)FGS-Amide to FM(ox)FGS-Nitrile

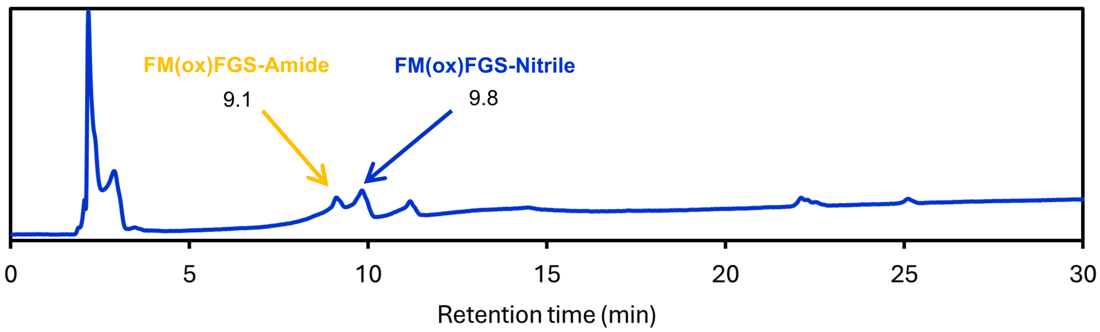

HRMS spectrum of fraction 9.8 min confirming the formation of FM(ox)FGS-Nitrile

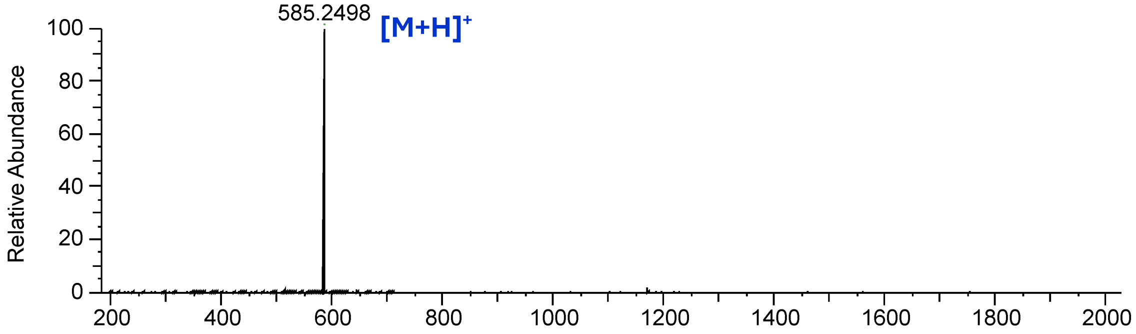

Entry 6

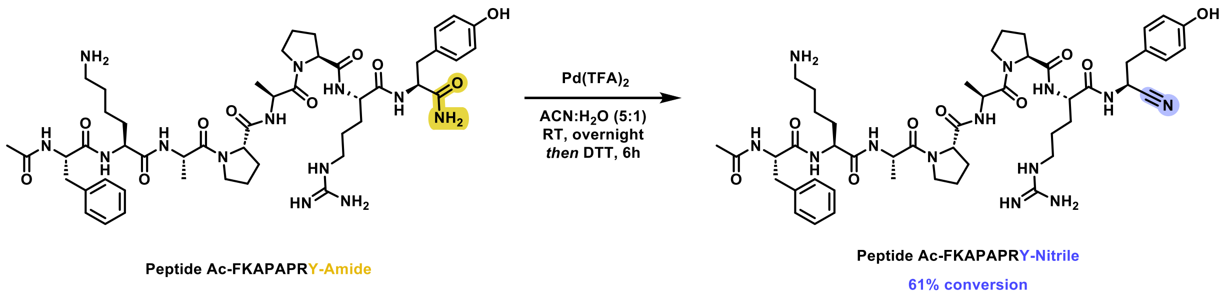

Peptide Ac-FKAPAPRY-Amide: **HRMS (ESI)** m/z: [M + H]+ Calcd 990.5520, Found 990.5527; [M + 2H]2+ Calcd 495.7796, Found 495.7817. **Analytical HPLC Method 1a**: retention time of 9.3 min.
Peptide Ac-FKAPAPRY-Nitrile: HRMS (ESI) m/z: [M + H]+ Calcd 972.5414, Found 972.5425; [M + 2H]2+ Calcd 486.7744, Found 486.7765. **Analytical HPLC Method 1a**: retention time of 10.5 min. Conversion: 61%.

Analytical HPLC trace of the purified peptide Ac-FKAPAPRY-Amide

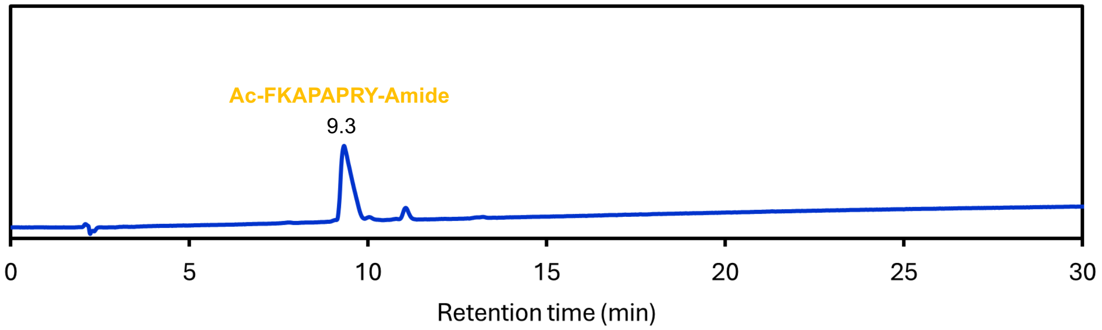

HRMS spectrum of the purified peptide Ac-FKAPAPRY-Amide

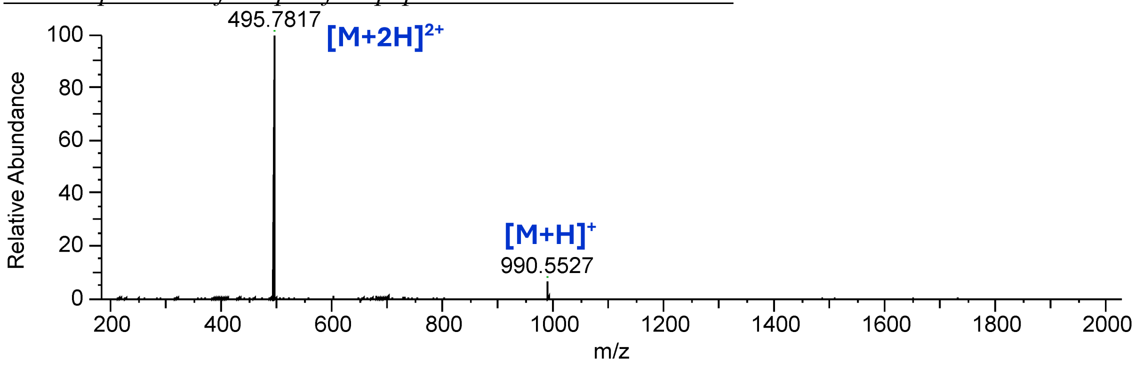

Analytical HPLC trace of the crude reaction converting Ac-FKAPAPRY-Amide to Ac-FKAPAPRY-Nitrile

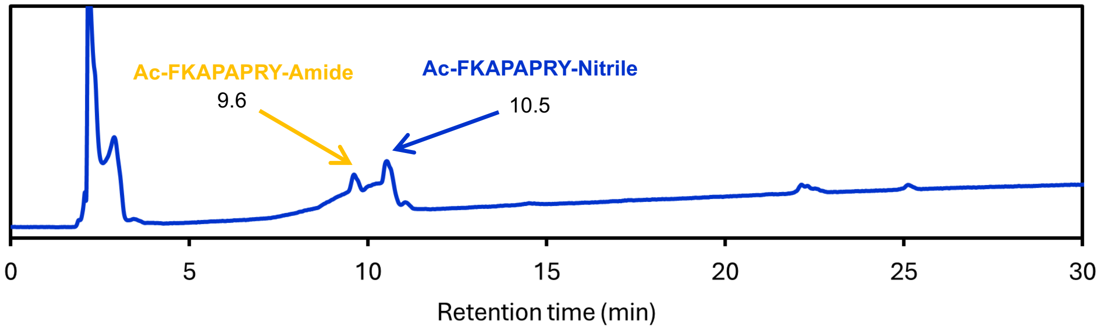

HRMS spectrum of fraction 10.5 min confirming the formation of Ac-FKAPAPRY-Nitrile

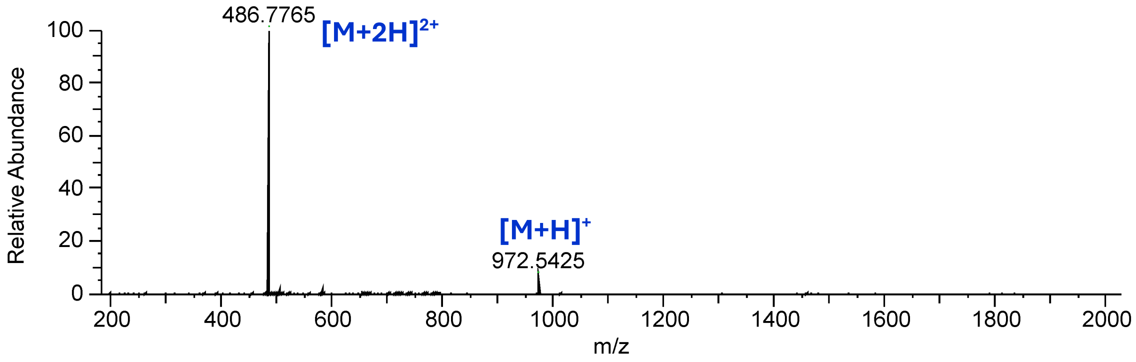

Entry 7

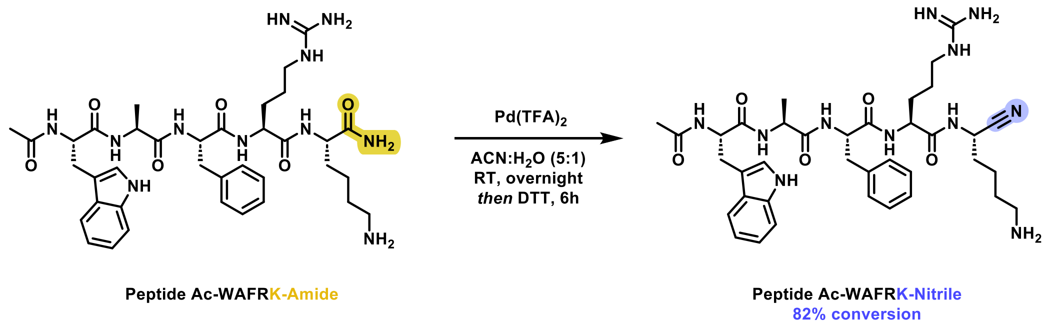

Peptide Ac-WAFRK-Amide: **HRMS (ESI)** m/z: $[M + H]^+$ Calcd 748.4253, Found 748.4254; $[M + 2H]^{2+}$ Calcd 374.7163, Found 374.7174. **Analytical HPLC Method 1a**: retention time of 10.3 min.

Peptide Ac-WAFRK-Nitrile: HRMS (ESI) m/z: $[M + 2H]^{2+}$ Calcd 365.7110, Found 365.7120. **Analytical HPLC Method 1a**: retention time of 11 min. Conversion: 82%.

Analytical HPLC trace of the purified peptide Ac-WAFRK-Amide

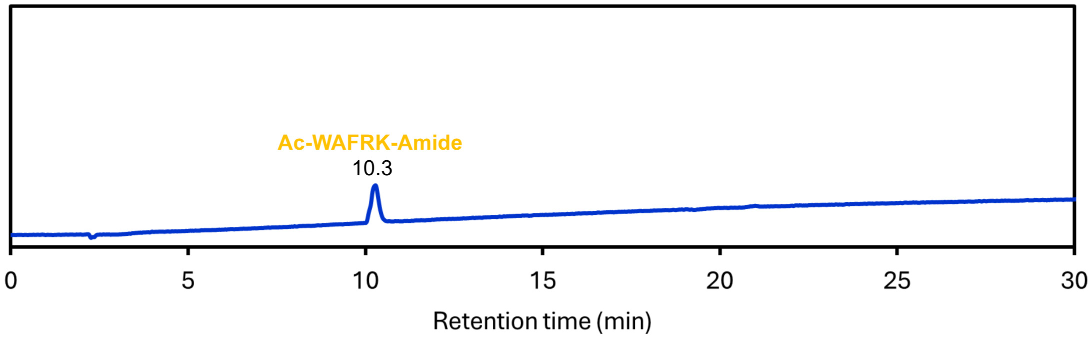

HRMS spectrum of the purified peptide Ac-WAFRK-Amide

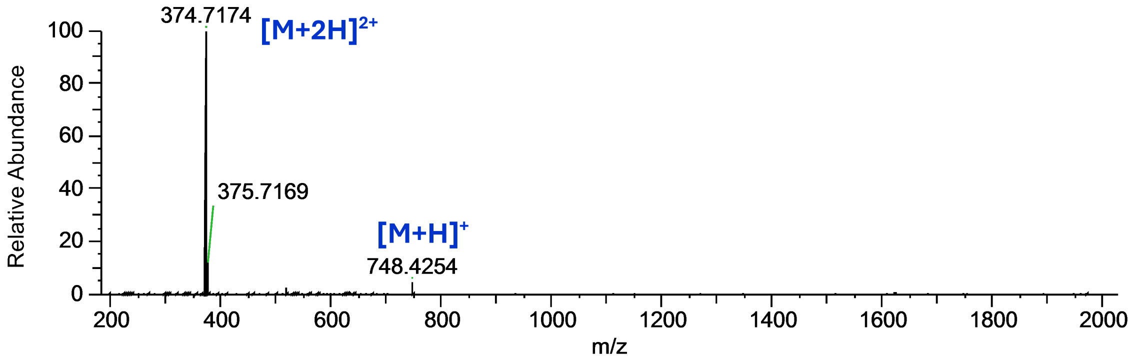

Analytical HPLC trace of the crude reaction converting Ac-WAFRK-Amide to Ac-WAFRK-Nitrile

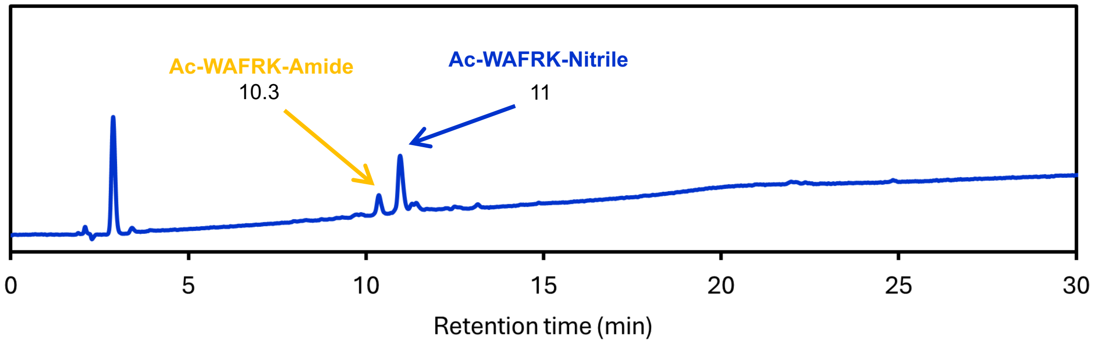

HRMS spectrum of fraction 11 min confirming the formation of Ac-WAFRK-Nitrile

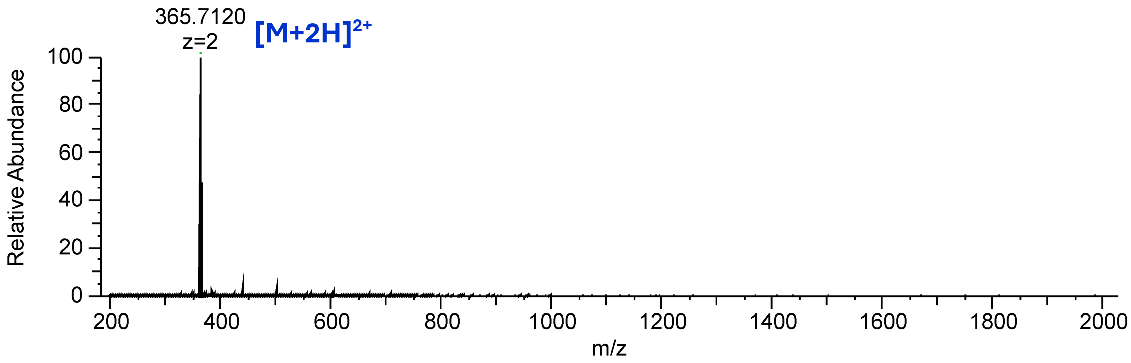

Entry 8

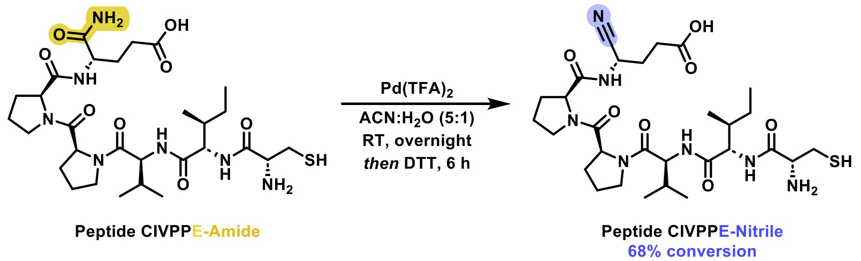

Peptide CIVPPE-Amide: **HRMS (ESI)** m/z: $[M + H]^+$ Calcd 656.3436, Found 656.3446; $[M + 2H]^{2+}$ Calcd 328.6755, Found 328.6758. **Analytical HPLC Method 1a**: retention time of 7.7 min.

Peptide CIVPPE-Nitrile: HRMS (ESI) m/z: $[M + H]^+$ Calcd 638.3330, Found 638.3344. **Analytical HPLC Method 1a**: retention time of 8.7 min. Conversion: 68%.

Analytical HPLC trace of the purified peptide CIVPPE-Amide

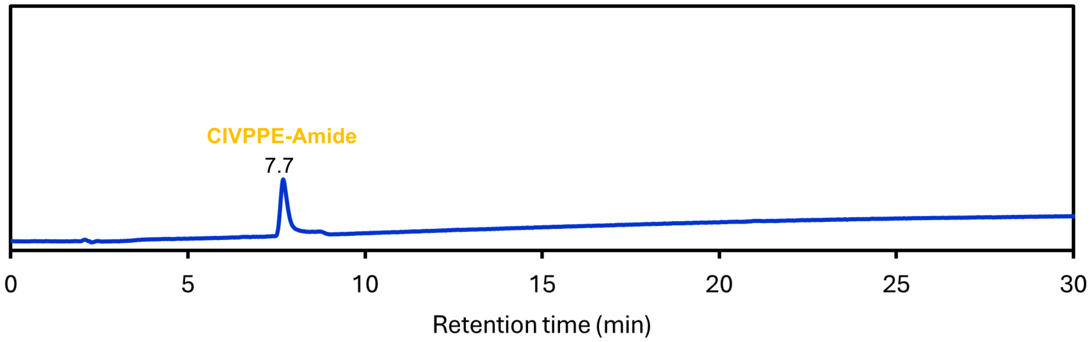

HRMS spectrum of the purified peptide CIVPPE-Amide

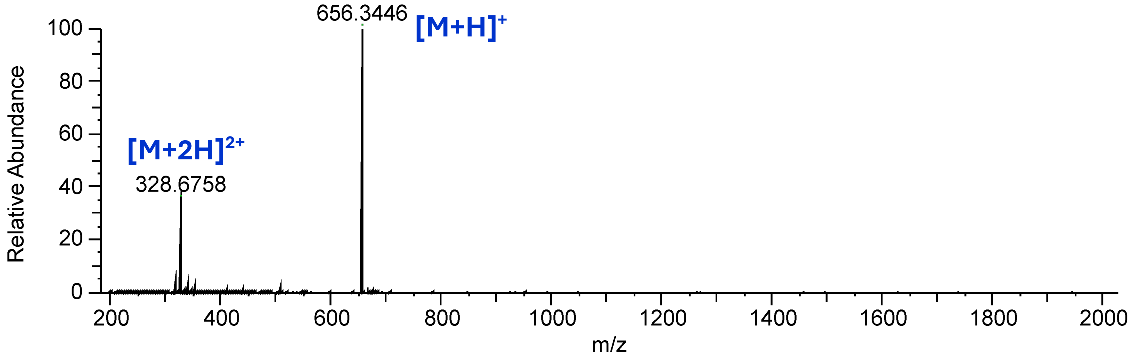

Analytical HPLC trace of the crude reaction converting CIVPPE-Amide to CIVPPE-Nitrile

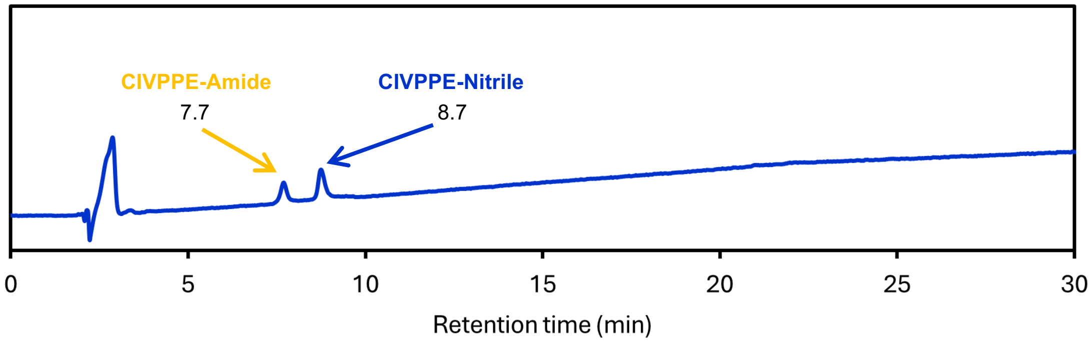

HRMS spectrum of fraction 8.7 min confirming the formation of CIVPPE-Nitrile

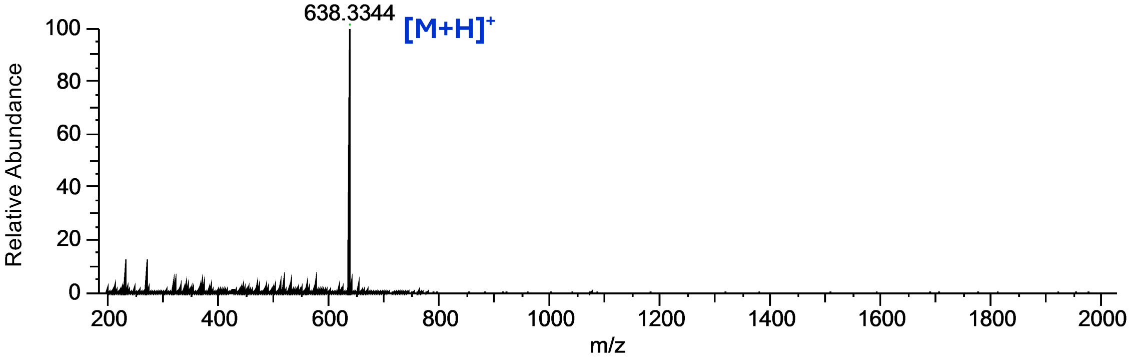

Entry 9

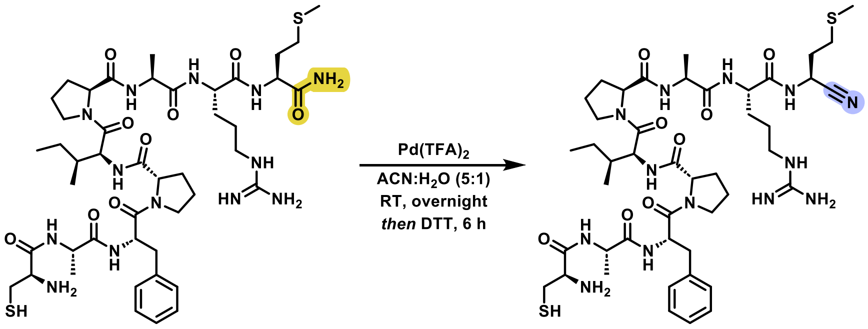

Peptide CAFPIPARM-Amide: **HRMS (ESI)** m/z: $[M + H]^+$ Calcd 1004.5168, Found 1004.5185; $[M + 2H]^{2+}$ Calcd 502.7621, Found 502.7639. **Analytical HPLC Method 1a**: retention time of 11.6 min. **Peptide CAFPIPARM-Nitrile: HRMS (ESI)** m/z: $[M + 2H]^{2+}$ Calcd 493.7568, Found 493.7585. **Analytical HPLC Method 1a**: retention time of 12.9 min. Conversion: >99%.

Analytical HPLC trace of the purified peptide CAFPIPARM-Amide

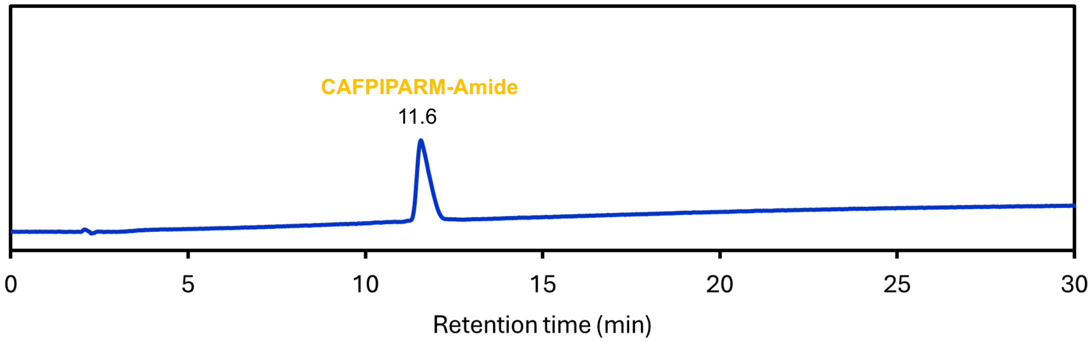

HRMS spectrum of the purified peptide CAFPIPARM-Amide

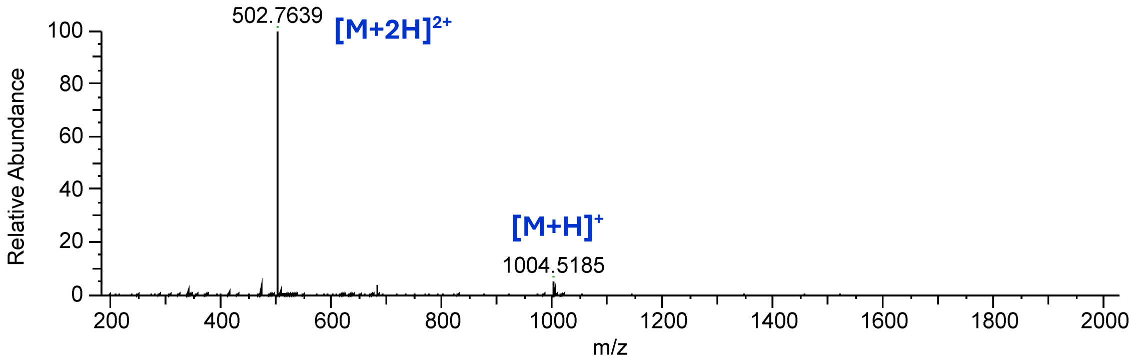

Analytical HPLC trace of the crude reaction converting CAFPIPARM-Amide to CAFPIPARM-Nitrile

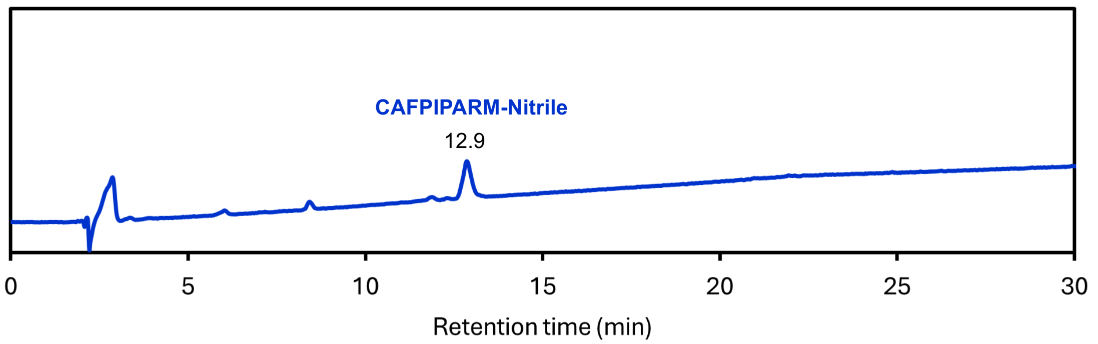

HRMS spectrum of fraction 12.9 min confirming the formation of CAFPIPARM-Nitrile

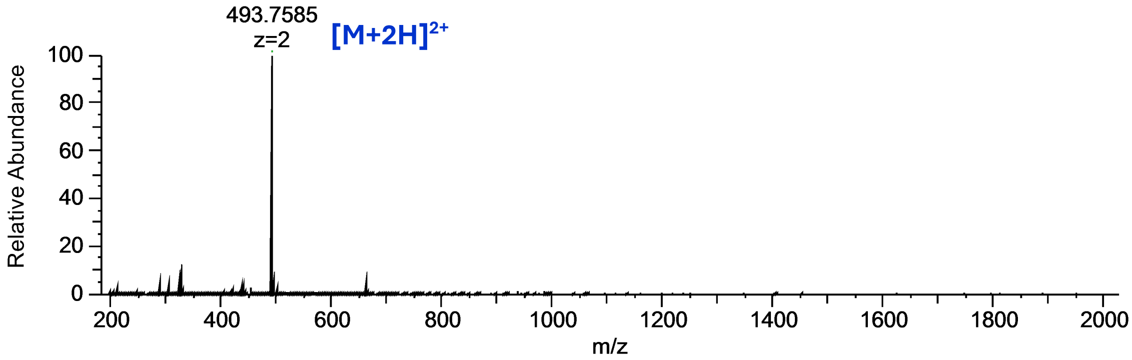

XX. Supplementary Figure 9. Converting peptide 1a to nitrile peptide 1b and thiazoline peptide 1c (Mollamide F).

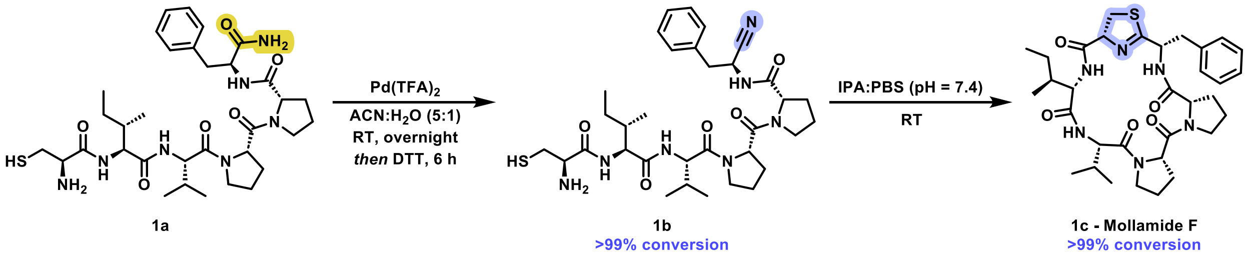

Procedure

The peptide **1a** (6.7 mg, 0.01 mmol, 1 eq) was subjected to general procedure A to afford the nitrile peptide **1b** (>99% conversion) and the thiazoline peptide **1c** (>99% conversion).

Peptide 1a: **HRMS (ESI)** m/z: $[M + H]^+$ Calcd 674.3700, Found 674.3478; $[M + Na]^+$ Calcd 696.3520, Found 696.3289; $[M + K]^+$ Calcd 712.3259, Found 712.3022. **Analytical HPLC Method 1a**: retention time of 12.8 min.

Peptide 1b: **HRMS (ESI)** m/z: $[M + H]^+$ Calcd 656.3594, Found 656.3460; $[M + Na]^+$ Calcd 678.3414, Found 678.3273; $[M + K]^+$ Calcd 694.3153, Found 694.3008. **Analytical HPLC Method 1a**: retention time of 14.8 min.

Peptide 1c (Mollamide F): **HRMS (ESI)** m/z: $[M + H]^+$ Calcd 639.3329, Found 639.3198; $[M + Na]^+$ Calcd 661.3148, Found 661.3010. **Analytical HPLC Method 1b**: retention time of 17.7 min.

Analytical HPLC trace of purified peptide 1a

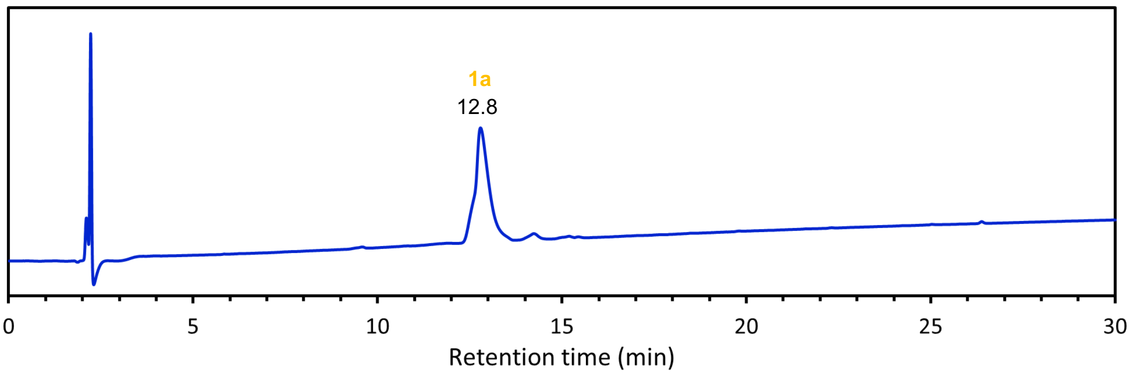

HRMS spectrum of purified peptide 1a

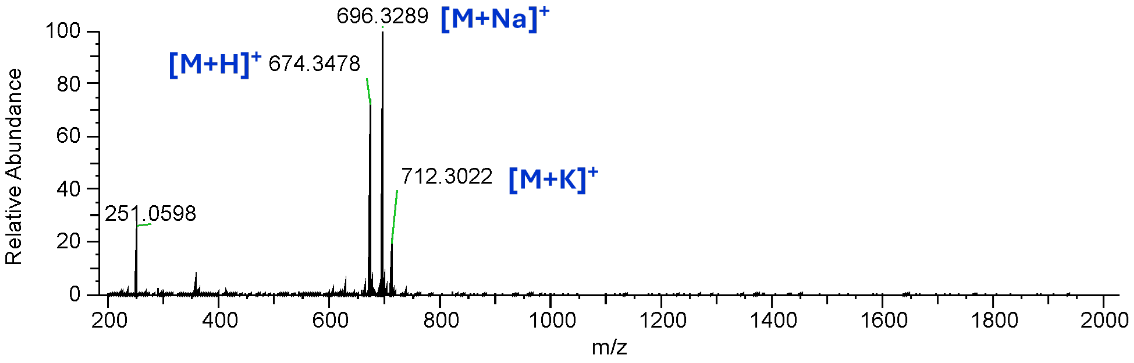

Analytical HPLC trace of the crude reaction converting peptide 1a to nitrile peptide 1b

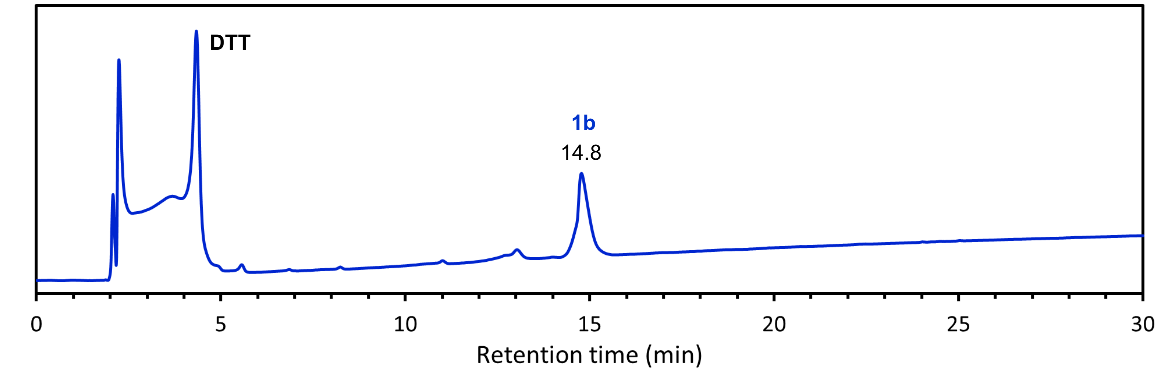

HRMS spectrum of fraction 14.8 min confirming the formation of nitrile peptide 1b

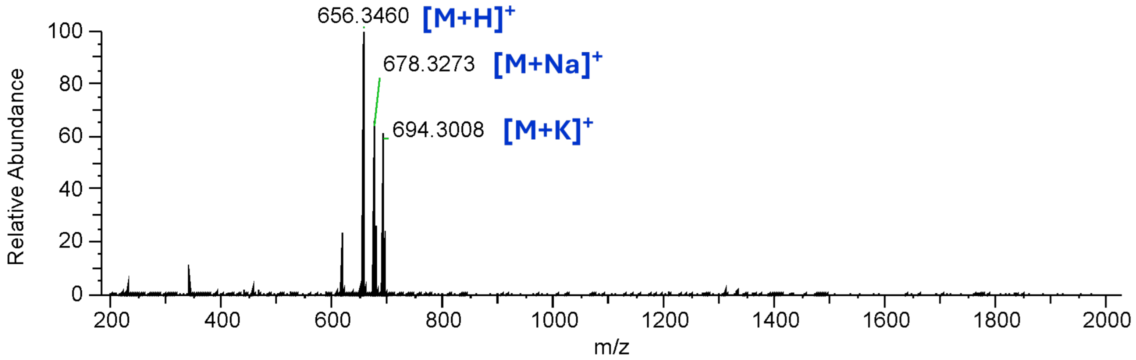

Analytical HPLC trace of the crude reaction converting peptide 1b to thiazoline peptide 1c (Mollamide F)

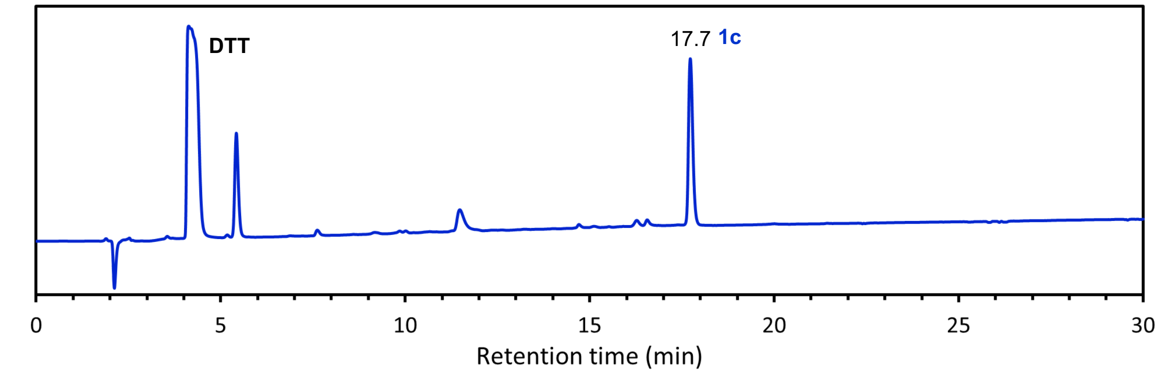

HRMS spectrum of fraction 17.7 min confirming the formation of thiazoline peptide 1c (Mollamide F)

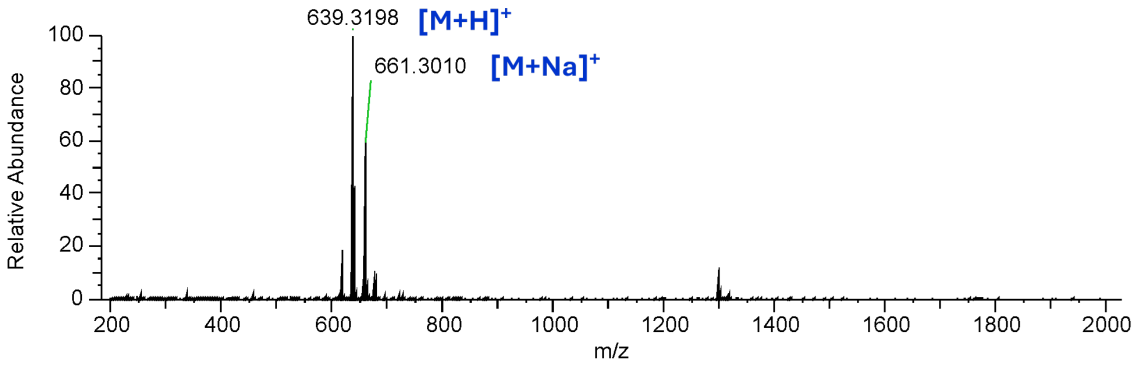

XXI. Supplementary Figure 10. Converting peptide 2a to nitrile peptide 2b and thiazoline peptide 2c.

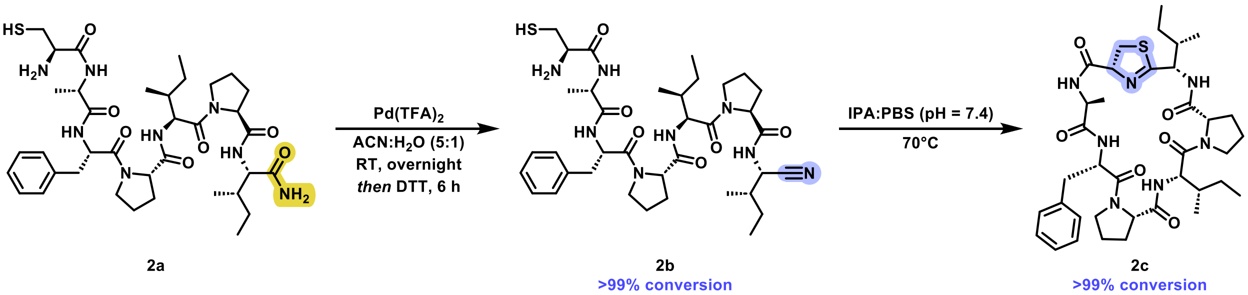

Procedure

The peptide **2a** (7.6 mg, 0.01 mmol, 1 eq) was subjected to general procedure A to afford the nitrile peptide **2b** (>99% conversion). The subsequent conversion of the nitrile peptide **2b** to the thiazoline peptide **2c** (>99% conversion) was conducted at 70 °C.

Peptide 2a: **HRMS (ESI)** m/z: $[M + H]^+$ Calcd 759.4227, Found 759.3975; $[M + Na]^+$ Calcd 781.4047, Found 781.3784. **Analytical HPLC Method 1a**: retention time of 13.6 min.

Peptide 2b: **HRMS (ESI)** m/z: $[M + H]^+$ Calcd 741.4122, Found 741.4091; $[M + Na]^+$ Calcd 763.3942, Found 763.3906. **Analytical HPLC Method 1a**: retention time of 16.1 min.

Peptide 2c: **HRMS (ESI)** m/z: $[M + H]^+$ Calcd 724.3856, Found 724.3851; $[M + Na]^+$ Calcd 746.3676, Found 746.3668; $[M + K]^+$ Calcd 762.3415, Found 762.3409. **Analytical HPLC Method 1b**: retention time of 21.6 min.

Analytical HPLC trace of purified peptide 2a

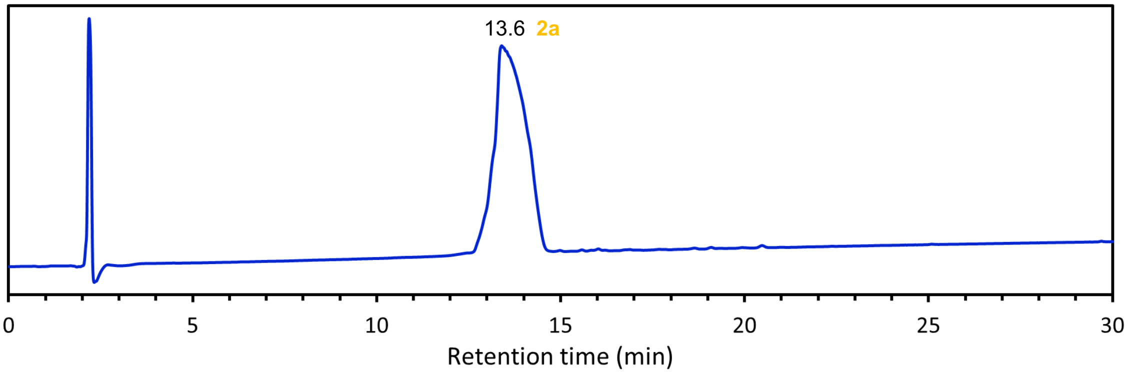

HRMS spectrum of purified peptide 2a

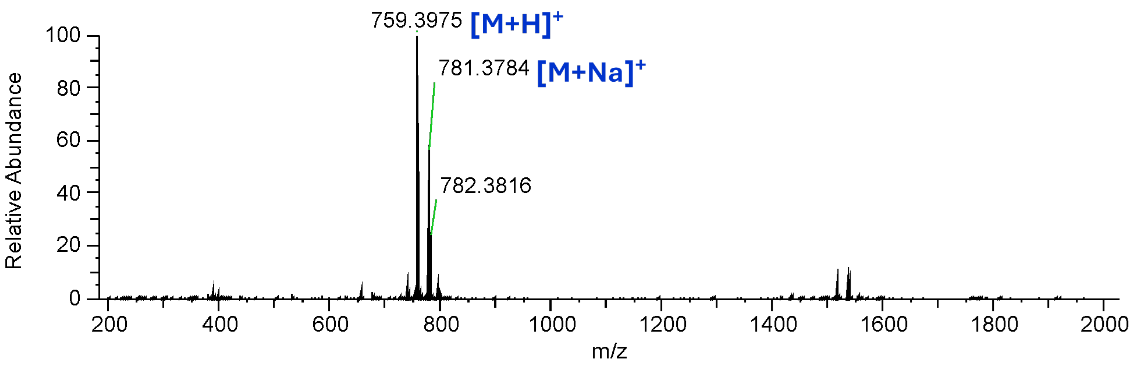

Analytical HPLC trace of the crude reaction converting peptide 2a to nitrile peptide 2b

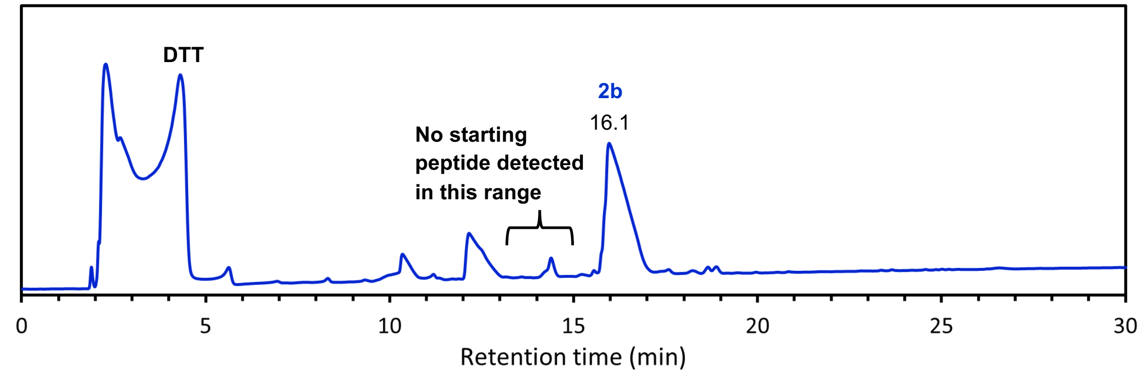

HRMS spectrum of fraction 16.1 min confirming the formation of nitrile peptide 2b

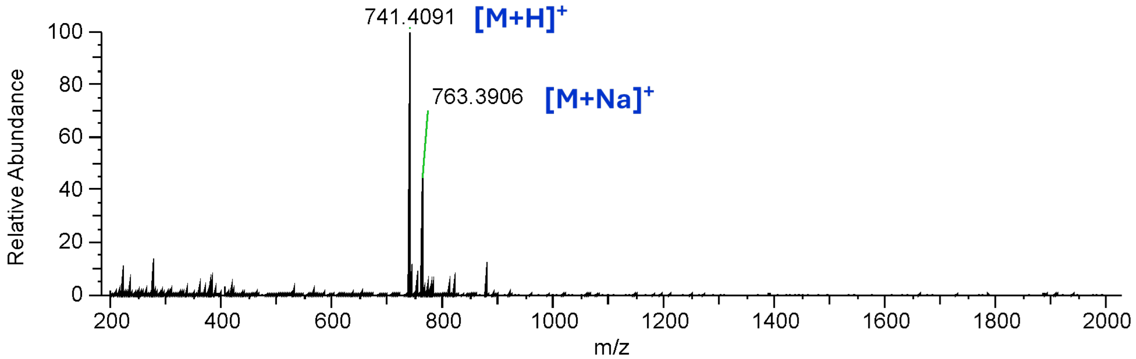

Analytical HPLC trace of the crude reaction converting nitrile peptide 2b to thiazoline peptide 2c

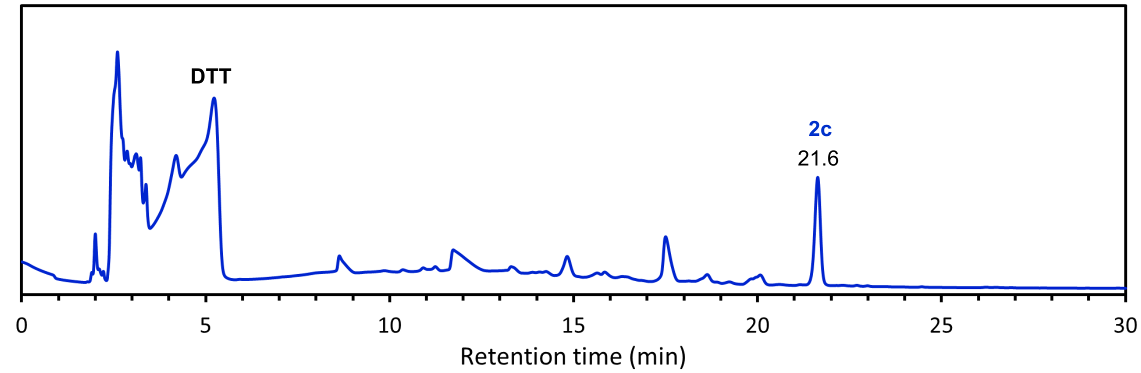

HRMS spectrum of fraction 21.6 min confirming the formation of thiazoline peptide 2c

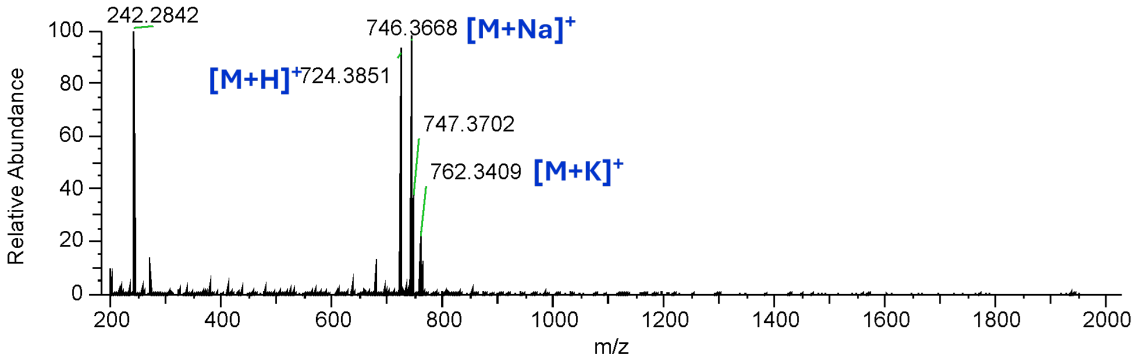

XXII. Supplementary Figure 11. Converting peptide 3a to nitrile peptide 3b and thiazoline peptide 3c.

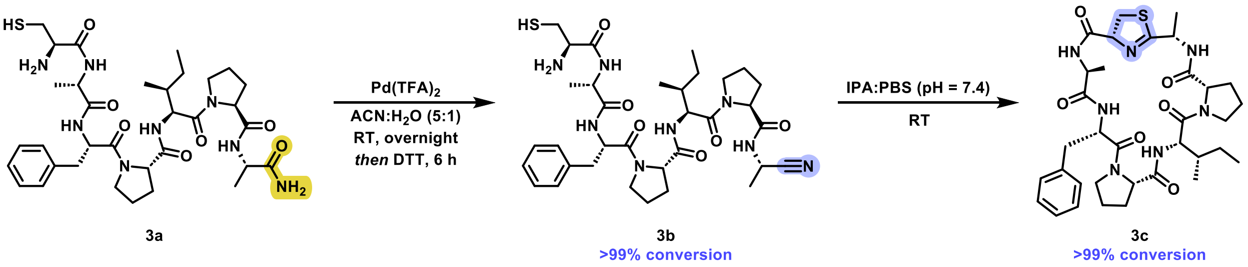

Procedure

The peptide **3a** (7.2 mg, 0.01 mmol, 1 eq) was subjected to general procedure A to afford the nitrile peptide **3b** (>99% conversion) and the thiazoline peptide **3c** (>99% conversion).

Peptide 3a: **HRMS (ESI)** m/z: $[M + H]^+$ Calcd 717.3758, Found 717.3751; $[M + Na]^+$ Calcd 739.3578, Found 739.3565. **Analytical HPLC Method 1a**: retention time of 11.8 min.
Peptide 3b: **HRMS (ESI)** m/z: $[M + H]^+$ Calcd 699.3652, Found 699.3649; $[M + Na]^+$ Calcd 721.3472, Found 721.3467; $[M + K]^+$ Calcd 737.3211, Found 737.3204. **Analytical HPLC Method 1a**: retention time of 12.9 min.
Peptide 3c: **HRMS (ESI)** m/z: $[M + H]^+$ Calcd 682.3387, Found 682.3383; $[M + Na]^+$ Calcd 704.3207, Found 704.3198. **Analytical HPLC Method 1b**: retention time of 19 min.

Analytical HPLC trace of purified peptide 3a

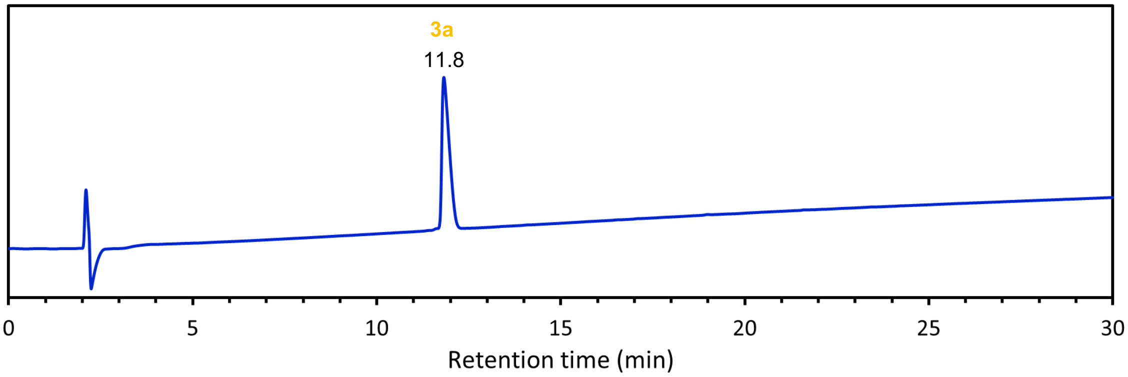

HRMS spectrum of purified peptide 3a

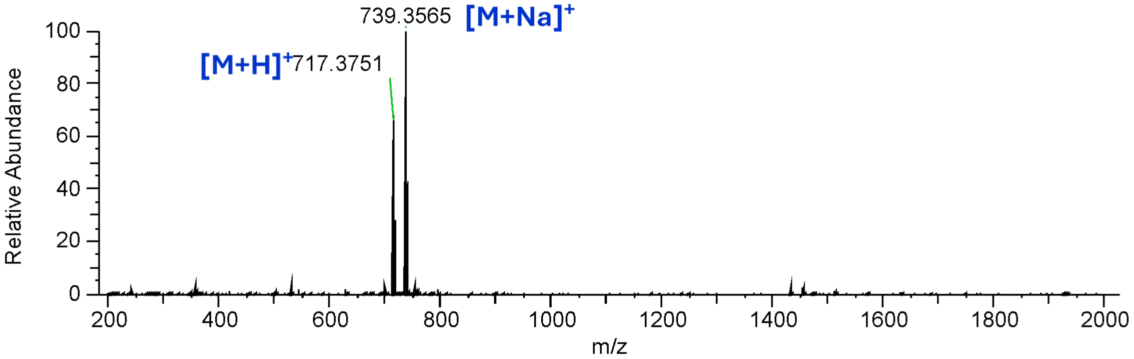

Analytical HPLC trace of the crude reaction converting peptide 3a to nitrile peptide 3b

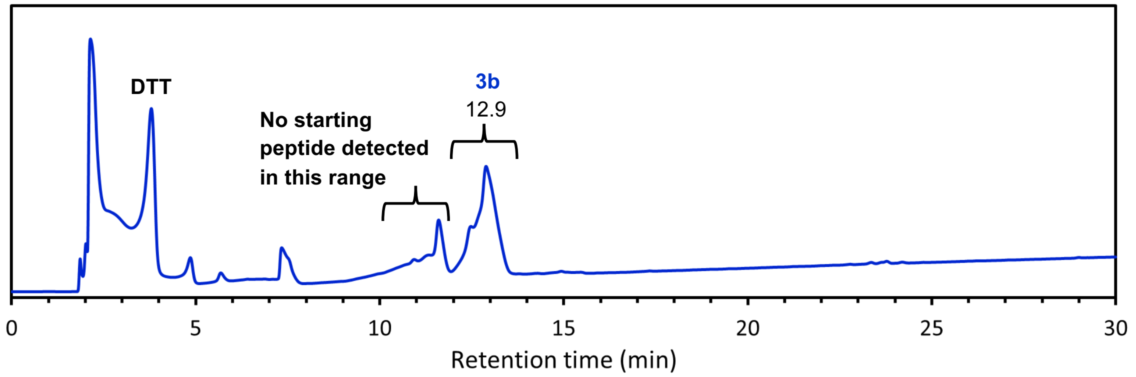

HRMS spectrum of fraction 12.9 min confirming the formation of nitrile peptide 3b

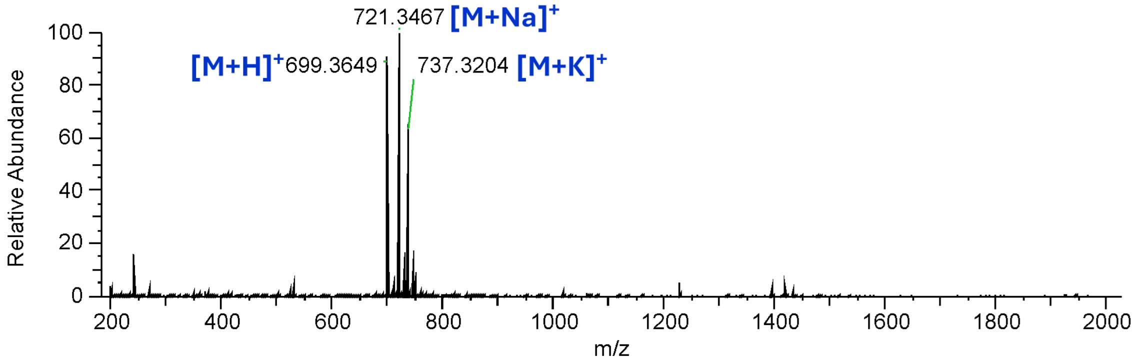

Analytical HPLC trace of the crude reaction converting nitrile peptide 3b to thiazoline peptide 3c

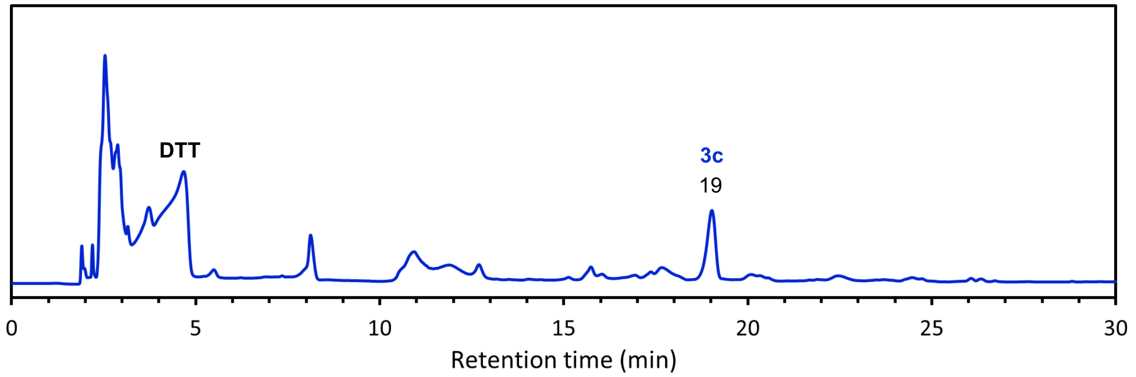

HRMS spectrum of fraction 19 min confirming the formation of thiazoline peptide 3c

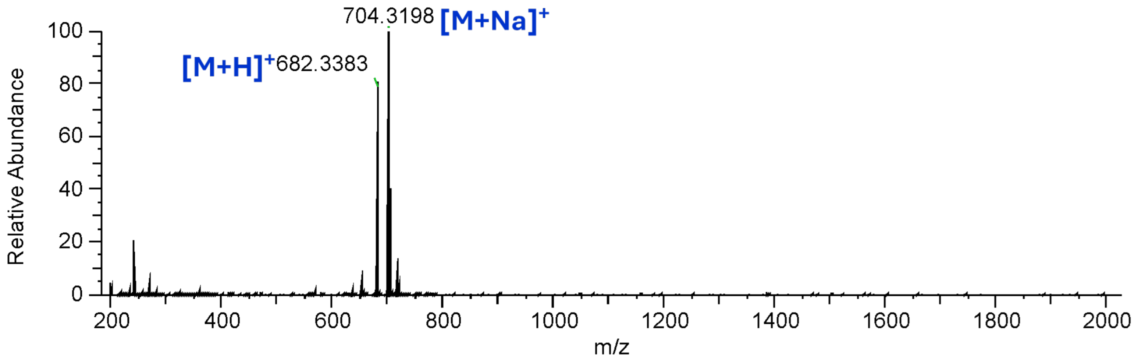

XXIII. Supplementary Figure 12. Converting peptide 4a to nitrile peptide 4b and thiazoline peptide 4c.

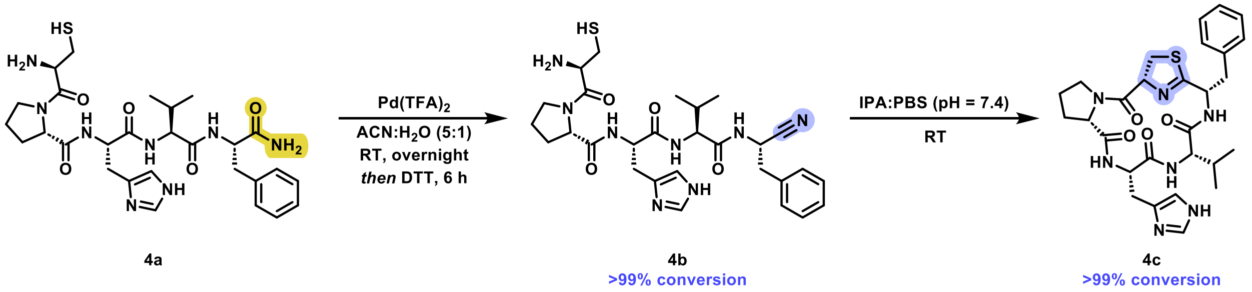

Procedure

The peptide **4a** (6 mg, 0.01 mmol, 1 eq) was subjected to general procedure A to afford the nitrile peptide **4b** (>99% conversion) and the thiazoline peptide **4c** (>99% conversion).

Peptide 4a: **HRMS (ESI)** m/z: $[M + H]^+$ Calcd 601.2921, Found 601.2688; $[M + Na]^+$ Calcd 623.2740, Found 623.2496; $[M + K]^+$ Calcd 639.2479, Found 639.2230. **Analytical HPLC Method 3a**: retention time of 11.1 min.

Peptide 4b: **HRMS (ESI)** m/z: $[M + H]^+$ Calcd 583.2815, Found 583.2809; $[M + Na]^+$ Calcd 605.2635, Found 605.2526. **Analytical HPLC Method 3a**: retention time of 14.7 min.

Peptide 4c: **HRMS (ESI)** m/z: $[M + H]^+$ Calcd 566.2549, Found 566.2544; $[M + Na]^+$ Calcd 588.2369, Found 588.2361; $[M + K]^+$ Calcd 604.2108, Found 604.2100. **Analytical HPLC Method 3b**: retention time of 20.6 min.

Analytical HPLC trace of purified peptide 4a

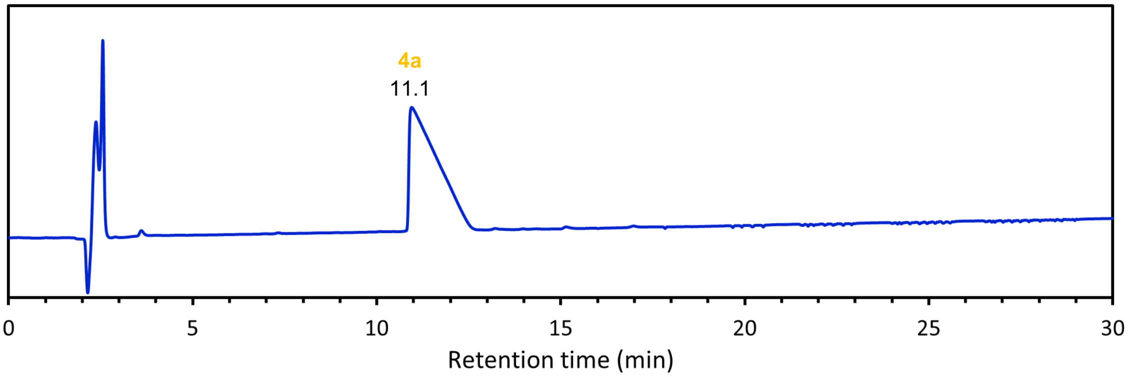

HRMS spectrum of purified peptide 4a

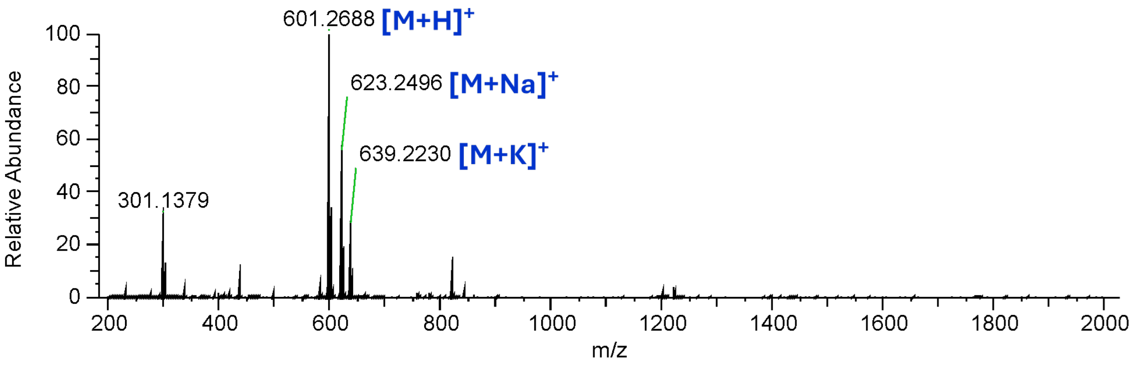

Analytical HPLC trace of the crude reaction converting peptide 4a to nitrile peptide 4b

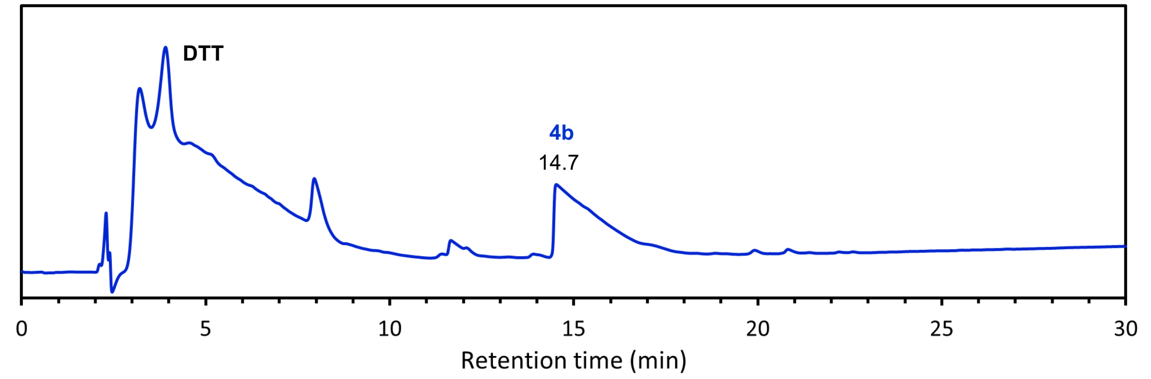

HRMS spectrum of fraction 14.7 min confirming the formation of nitrile peptide 4b

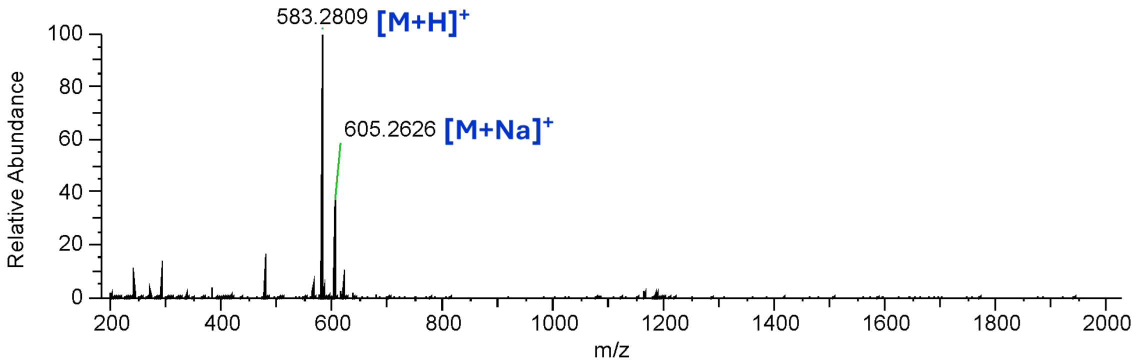

Analytical HPLC trace of the crude reaction converting nitrile peptide 4b to thiazoline peptide 4c

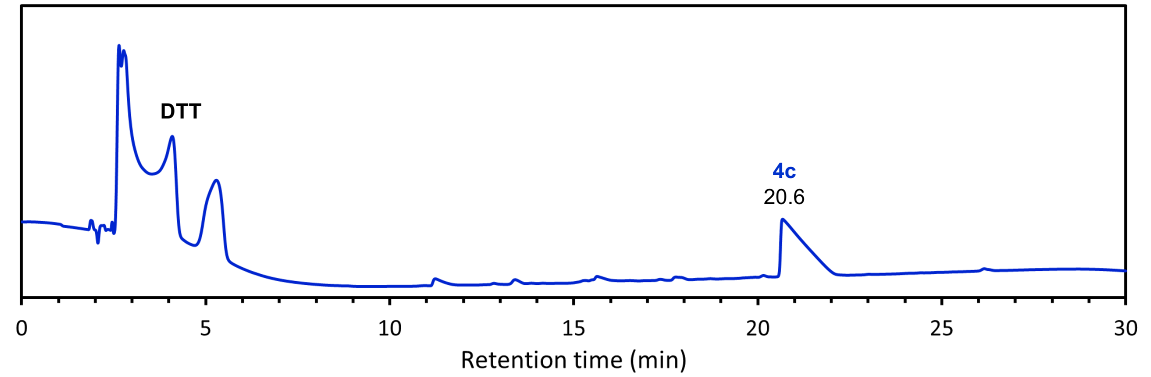

HRMS spectrum of fraction 20.6 min confirming the formation of thiazoline peptide 4c

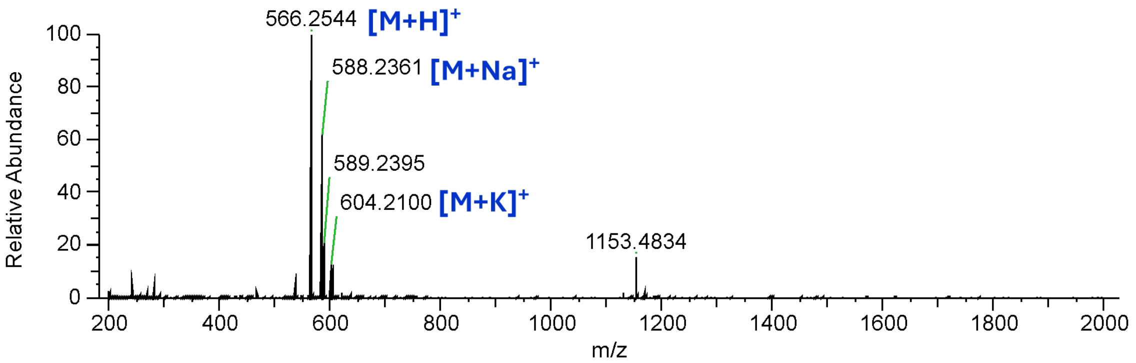

XXIV. Supplementary Figure 13. Converting peptide 5a to nitrile peptide 5b and thiazoline peptide 5c.

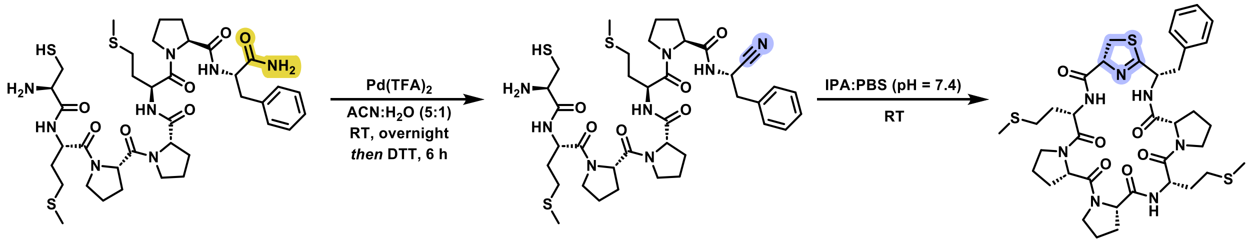

5a **5b** >99% conversion **5c** >99% conversion

Procedure

The peptide **5a** (8.2 mg, 0.01 mmol, 1 eq) was subjected to general procedure A to afford the nitrile peptide **5b** (>99% conversion) and the thiazoline peptide **5c** (>99% conversion).

Peptide 5a: **HRMS (ESI)** m/z: $[M + H]^+$ Calcd 821.3512, Found 821.3508; $[M + Na]^+$ Calcd 843.3332, Found 843.3323. **Analytical HPLC Method 1a**: retention time of 13.1 min.
Peptide 5b: **HRMS (ESI)** m/z: $[M + H]^+$ Calcd 803.3407, Found 803.3404. **Analytical HPLC Method 1a**: retention time of 15.2 min.
Peptide 5c: **HRMS (ESI)** m/z: $[M + H]^+$ Calcd 786.3141, Found 786.3141; $[M + Na]^+$ Calcd 808.2961, Found 808.2954. **Analytical HPLC Method 1b**: retention time of 21.0 min.

Analytical HPLC trace of the purified peptide 5a

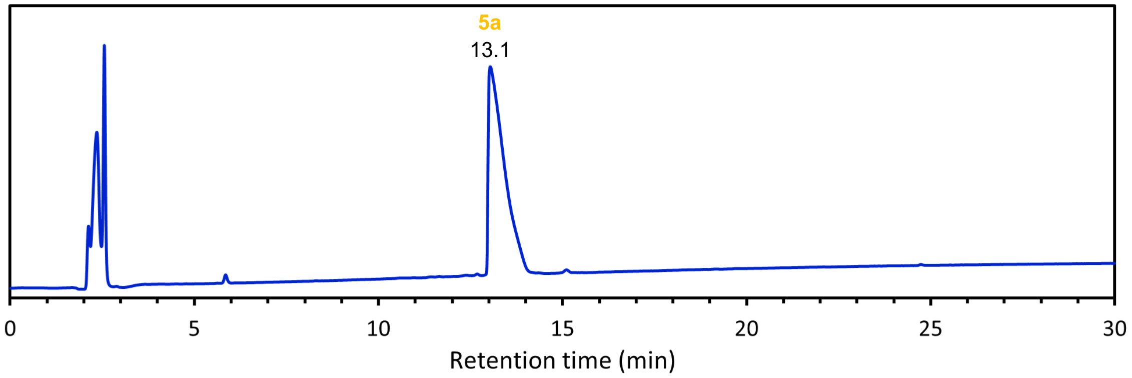

HRMS spectrum of the purified peptide 5a

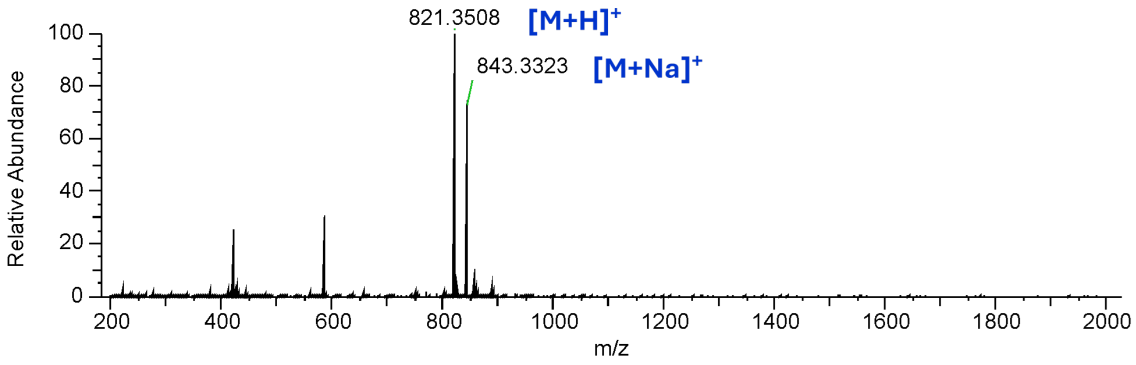

Analytical HPLC trace of the crude reaction converting peptide 5a to nitrile peptide 5b

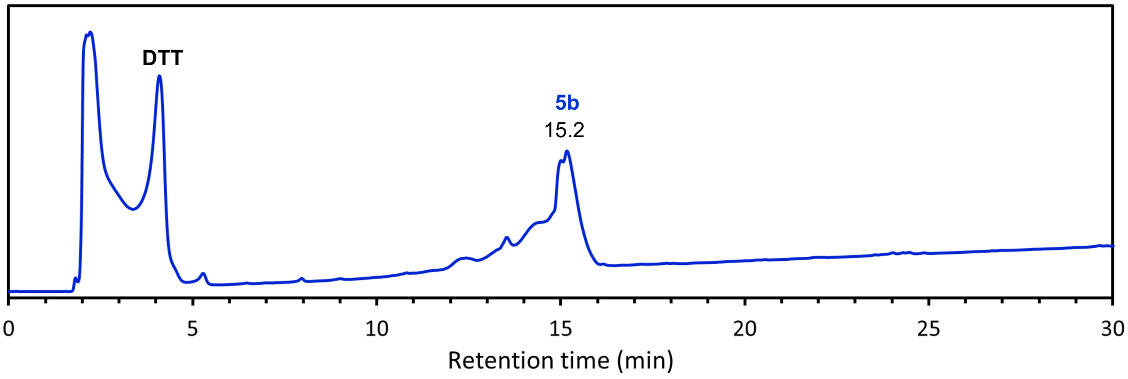

HRMS spectrum of fraction 15.2 min confirming the formation of the nitrile peptide 5b

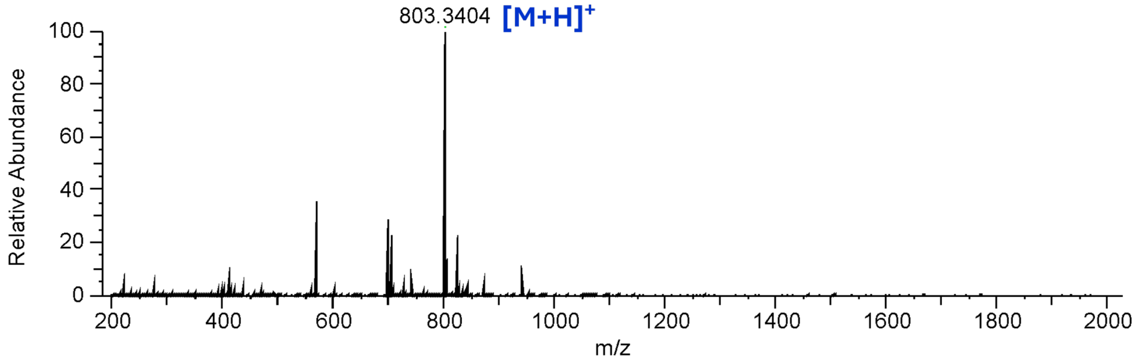

Analytical HPLC trace of the crude reaction converting nitrile peptide 5b to thiazoline peptide 5c

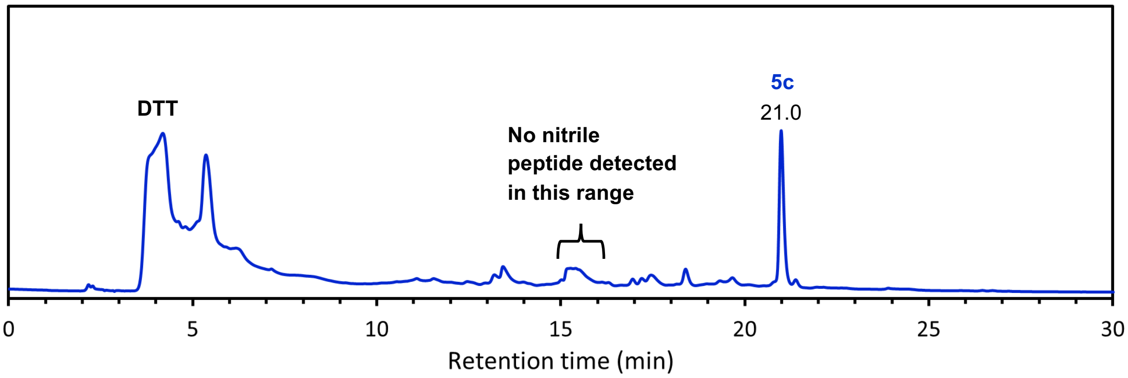

HRMS spectrum of fraction 21 min confirming the formation of the thiazoline peptide 5c

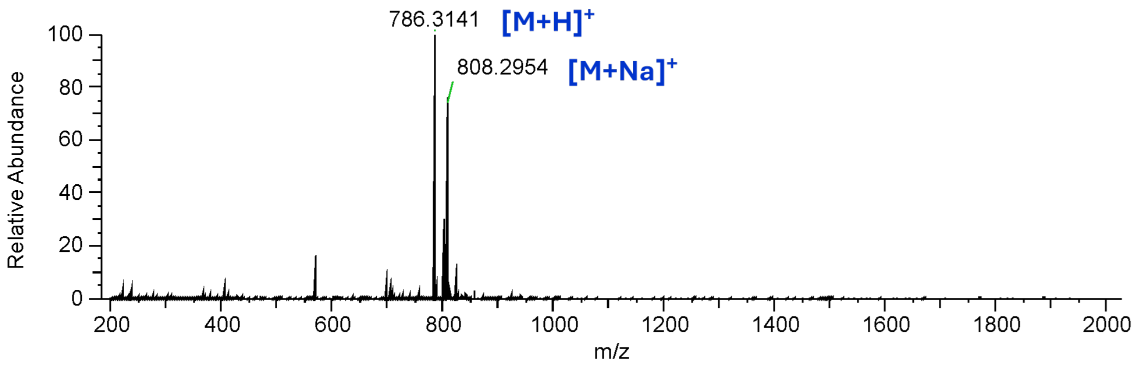

XXV. Supplementary Figure 14. Converting peptide 6a to nitrile peptide 6b and thiazoline peptide 6c (Phakellistatin 13 analog).

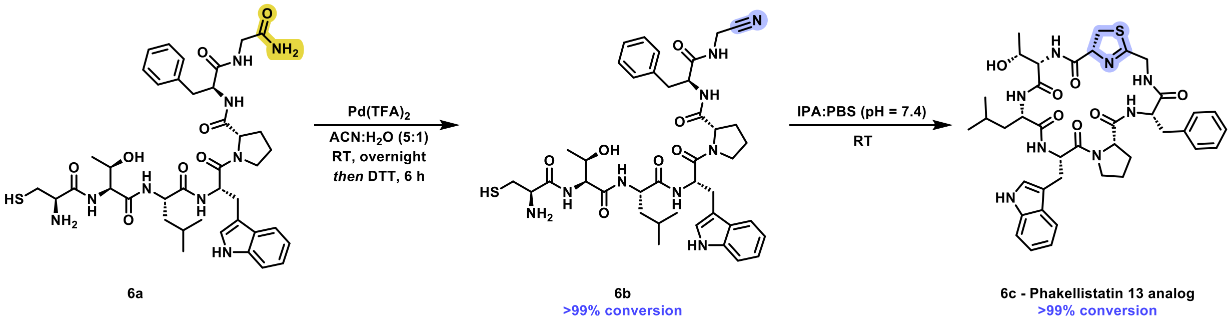

Procedure

The peptide **6a** (8.2 mg, 0.01 mmol, 1 eq) was subjected to general procedure A to afford the nitrile peptide **6b** (>99% conversion) and the thiazoline peptide **6c** (>99% conversion).

Peptide 6a: **HRMS (ESI)** m/z: $[M + H]^+$ Calcd 822.3973, Found 822.3712. **Analytical HPLC Method 1a**: retention time of 13.1 min.
Peptide 6b: **HRMS (ESI)** m/z: $[M + H]^+$ Calcd 804.3867, Found 804.3862; $[M + Na]^+$ Calcd 826.3687, Found 826.3679; $[M + K]^+$ Calcd 842.3426, Found 842.3419. **Analytical HPLC Method 1a**: retention time of 15.2 min.
Peptide 6c: **HRMS (ESI)** m/z: $[M + H]^+$ Calcd 787.3601, Found 787.3597; $[M + Na]^+$ Calcd 809.3421, Found 809.3414. **Analytical HPLC Method 1b**: retention time of 21.0 min.

Analytical HPLC trace of the purified peptide 6a

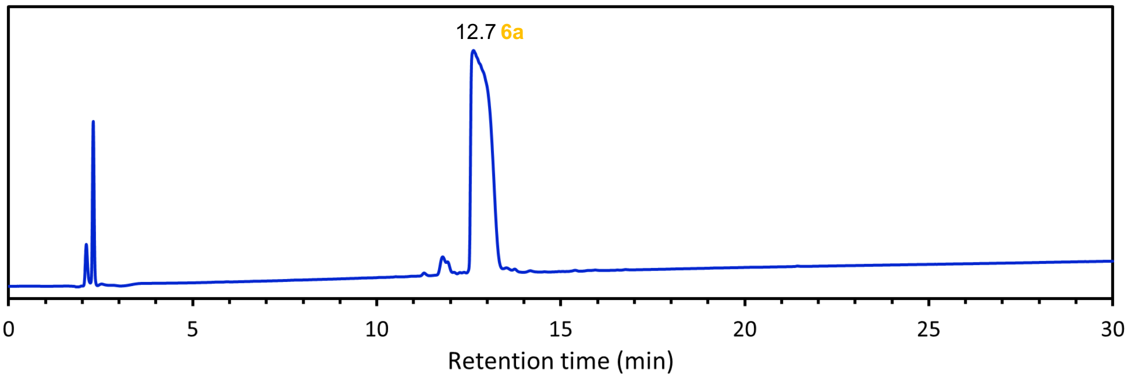

HRMS spectrum of the purified peptide 6a

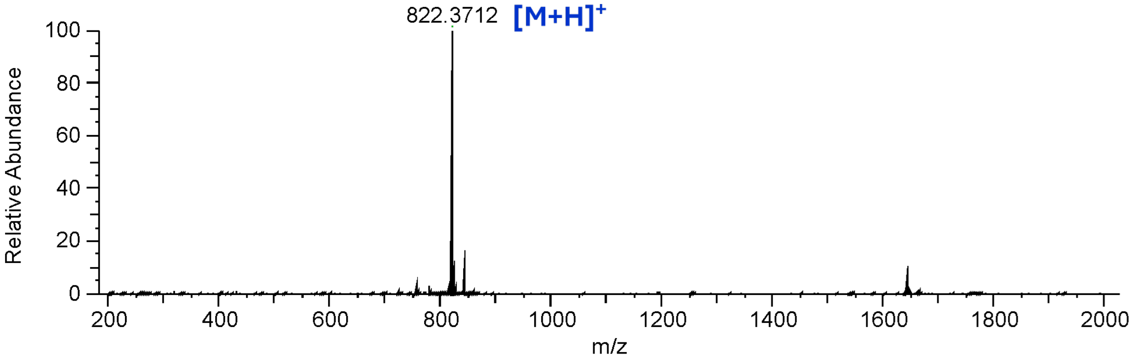

Analytical HPLC trace of the crude reaction converting peptide 6a to nitrile peptide 6b

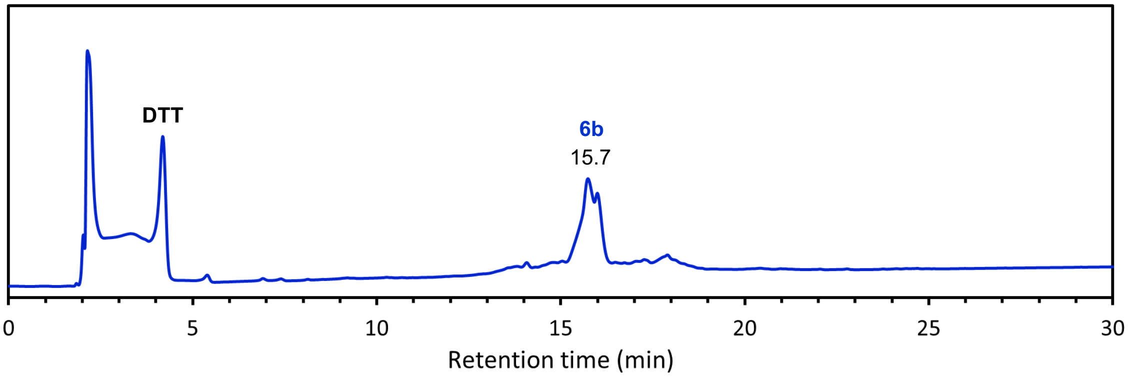

HRMS spectrum of fraction 15.7 min confirming the formation of the nitrile peptide 6b

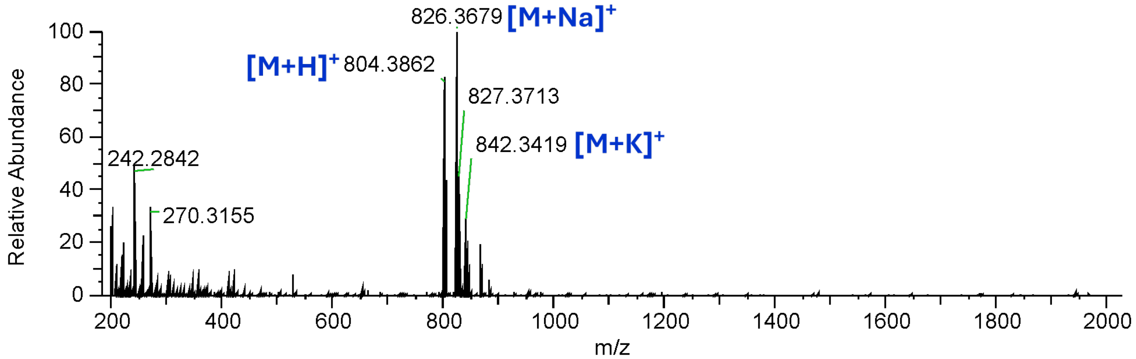

Analytical HPLC trace of the crude reaction converting nitrile 6b to thiazoline peptide 6c (Phakellistatin 13 analog)

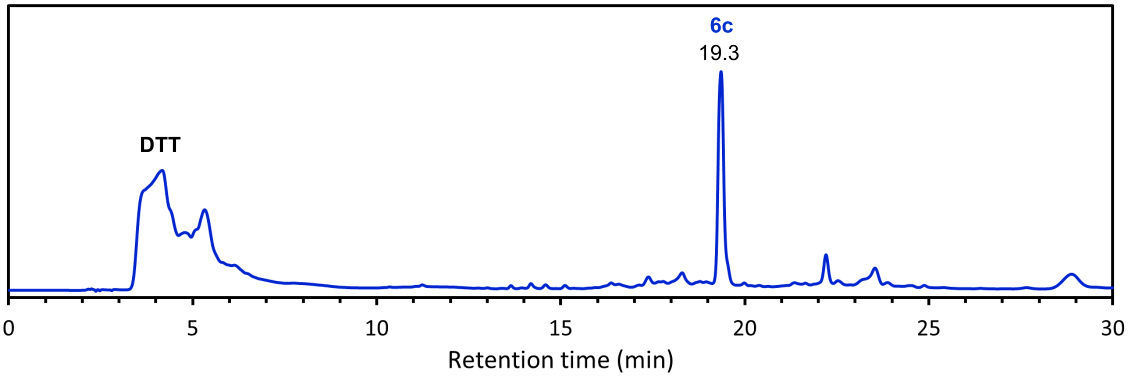

HRMS spectrum of fraction 19.3 min confirming the formation of Phakellistatin 13 analog

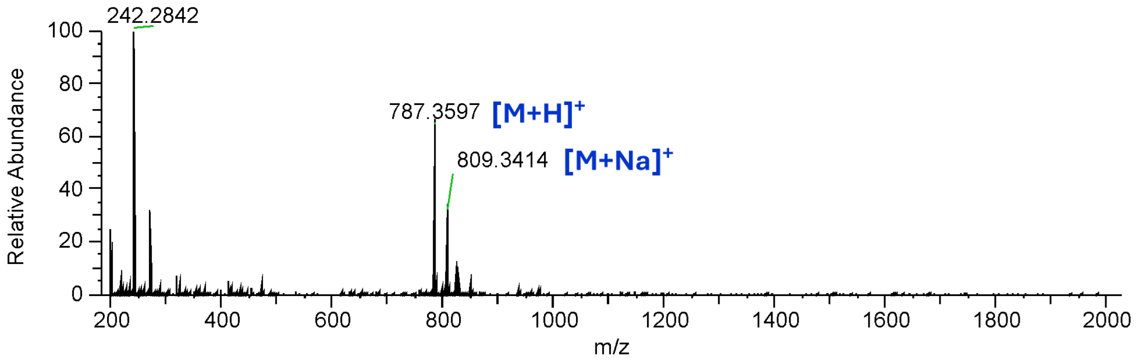

XXVI. Supplementary Table 4. Scope of thiazoline formation across diverse C-terminal amino acids with unprotected reactive side chains.

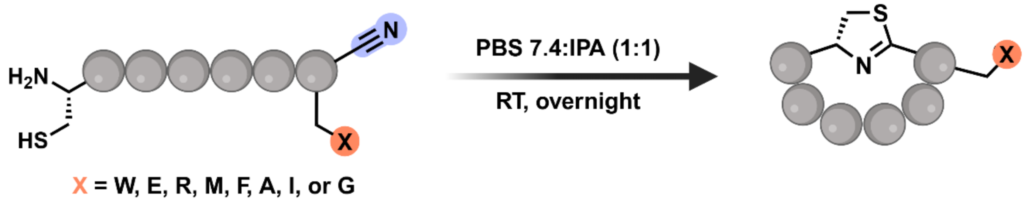

Entry	Peptide	Conversion (%)
1	H_2N-CIVPPW-Nitrile	>99
2	H_2N-CIVPPE-Nitrile	>95
3	H_2N-CAFPIPAR-Nitrile	>99
4	H_2N-CAFPIPARM-Nitrile	>99
5	H_2N-CIVPPF-Nitrile	>99
6	H_2N-CAFPIPA-Nitrile	>99
7	H_2N-CAFPIPI-Nitrile	>99
8	H_2N-CPHVF-Nitrile	>99
9	H_2N-CMPPMPF-Nitrile	>99
10	H_2N-CTLWPFG-Nitrile	>99

Procedure

The following peptides (0.01 mmol, 1 eq) were subjected to general procedure A. **Analytical HPLC Method 1a** was used to determine the conversion from the C-terminal amide to the corresponding C-terminal nitrile. **Analytical HPLC Method 1b** was used to determine the conversion from the C-terminal nitrile to the corresponding thiazoline. For entries 3 and 4, **Analytical HPLC Method 4** was used to determine the conversion from the C-terminal nitrile to the corresponding thiazoline. Entries 5-10 were shown above in sections XX-XXV (Figure 9-14).

Entry 1

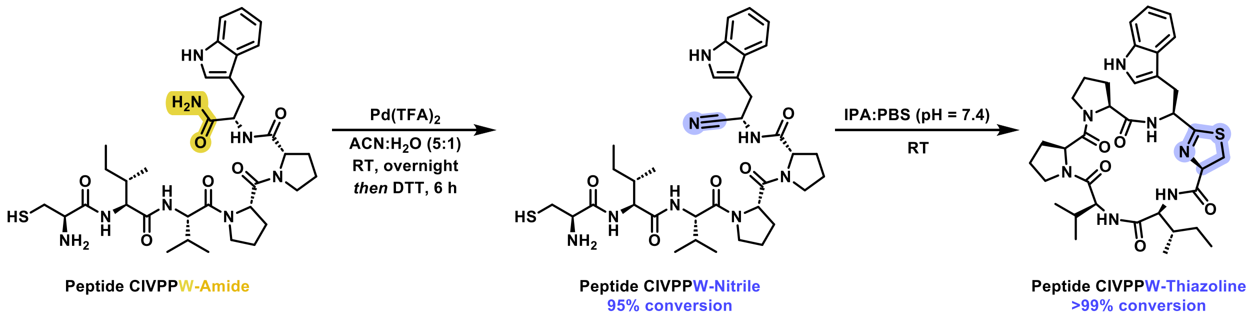

Peptide CIVPPW-Amide: **HRMS (ESI)** m/z: $[M + H]^+$ Calcd 713.3803, Found 713.3815; $[M + 2H]^{2+}$ Calcd 357.1938, Found 357.1942. **Analytical HPLC Method 1a**: retention time of 12.4 min.
Peptide CIVPPW-Nitrile: **HRMS (ESI)** m/z: $[M + H]^+$ Calcd 695.3698, Found 695.3712. **Analytical HPLC Method 1a**: retention time of 13.8 min. Conversion: 95%.
Peptide CIVPPW-Thiazoline: **HRMS (ESI)** m/z: $[M + H]^+$ Calcd 678.3432, Found 678.3448. **Analytical HPLC Method 1b**: retention time of 18.2 min. Conversion: >99%.

Analytical HPLC trace of the purified peptide CIVPPW-Amide

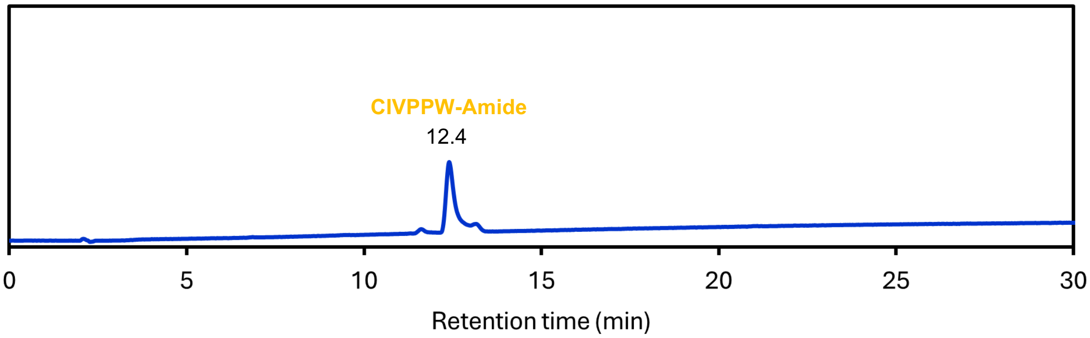

HRMS spectrum of the purified peptide CIVPPW-Amide

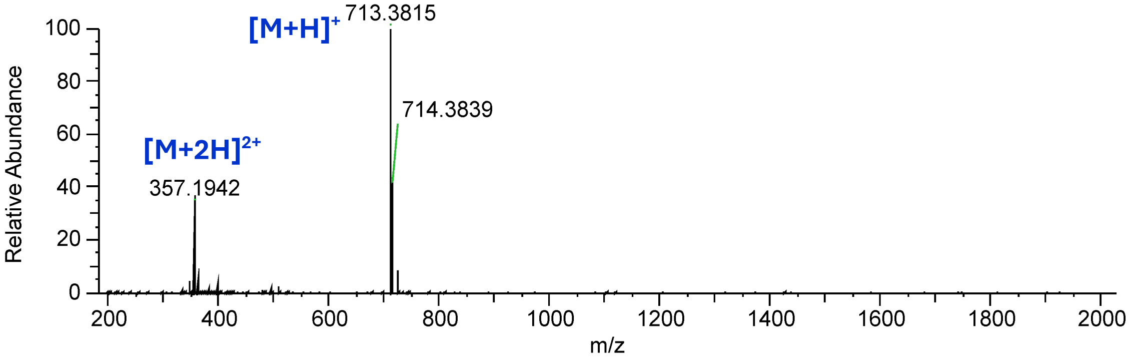

Analytical HPLC trace of the crude reaction converting CIVPPW-Amide to CIVPPW-Nitrile

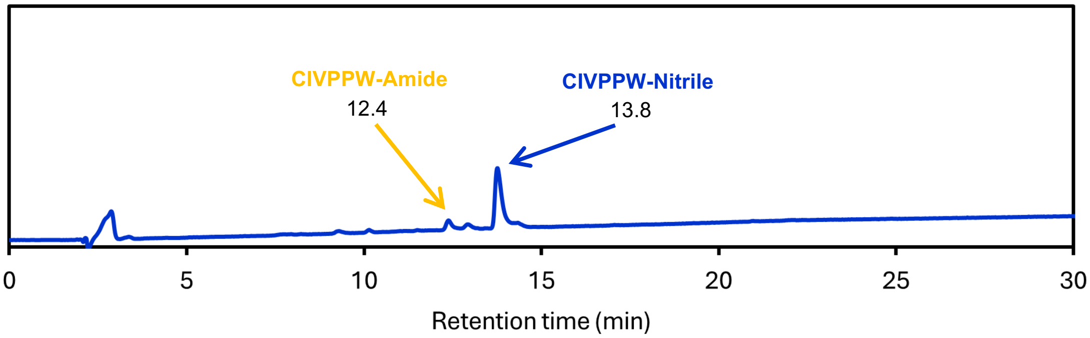

HRMS spectrum of fraction 13.8 min confirming the formation of CIVPPW-Nitrile

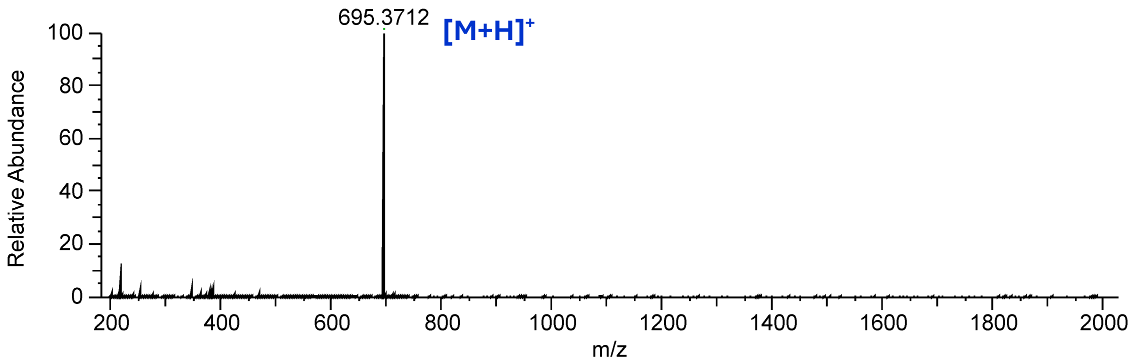

Analytical HPLC trace of the crude reaction converting CIVPPW-Nitrile to CIVPPW-Thiazoline

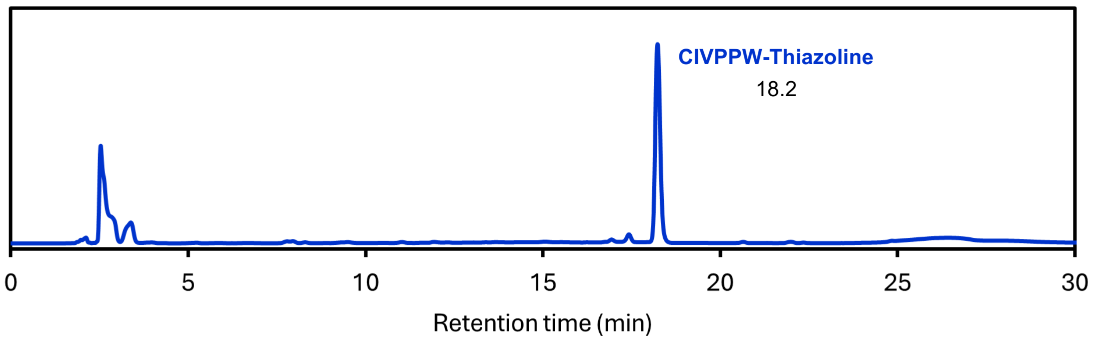

HRMS spectrum of fraction 18.2 min confirming the formation of CIVPPW-Thiazoline

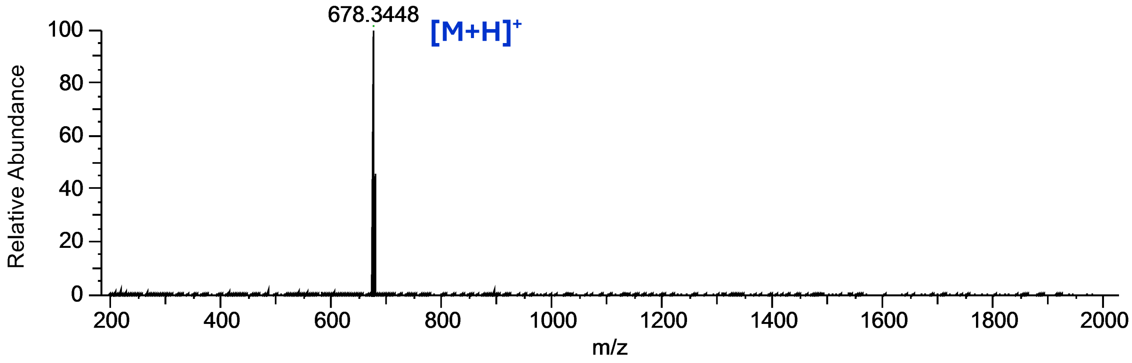

Entry 2

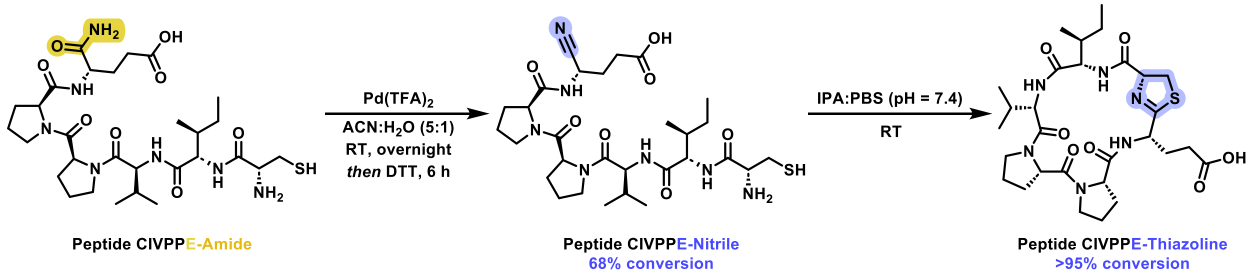

Peptide CIVPPE-Amide and Peptide CIVPPE-Nitrile: data was shown in section XIX, table 3, entry 8. **Peptide CIVPPE-Thiazoline**: **HRMS (ESI)** m/z: $[M + H]^+$ Calcd 621.3065, Found 621.3079. **Analytical HPLC Method 1b**: retention time of 13.1 min. Conversion: >95%.

Analytical HPLC trace of the crude reaction converting CIVPPE-Nitrile to CIVPPE-Thiazoline

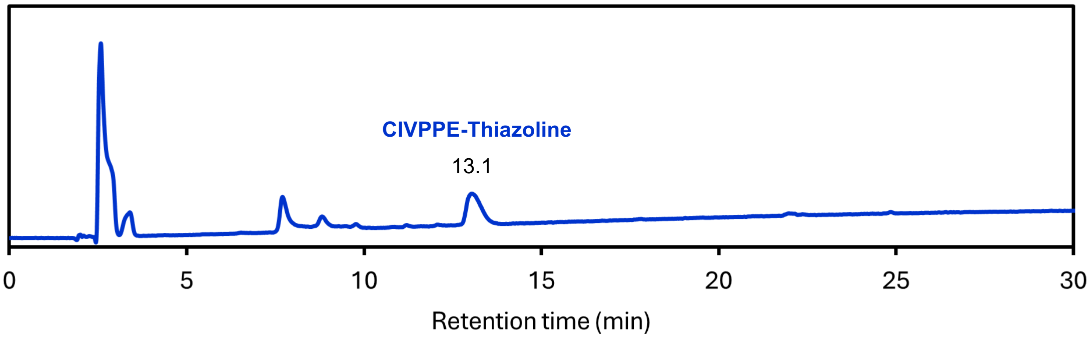

HRMS spectrum of fraction 13.1 min confirming the formation of CIVPPW-Thiazoline

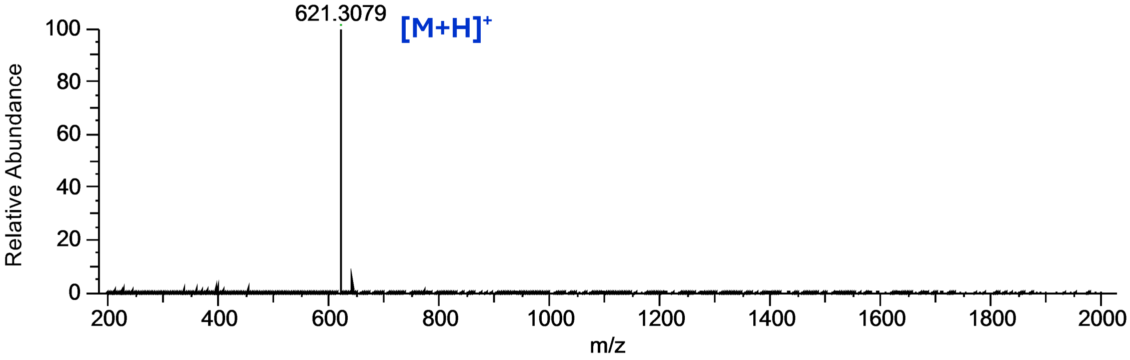

Entry 3

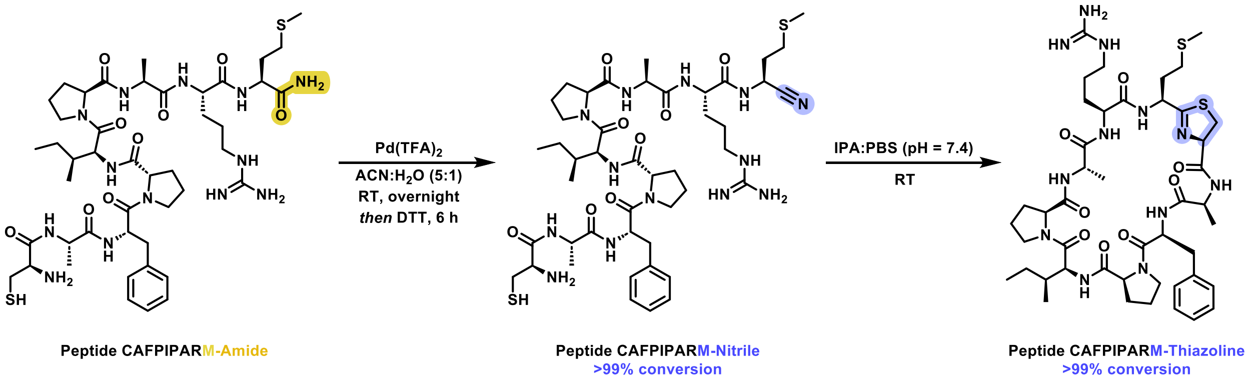

Peptide CAFPIPARM-Amide and Peptide CAFPIPARM-Nitrile: data was shown in section XIX, table 3, entry 9.

Because the formation of **CAFPIPARM-Thiazoline** was analyzed using **Analytical HPLC Method 4**, the crude reaction converting CAFPIPARM-Amide to CAFPIPARM-Nitrile was analyzed again using **Analytical HPLC Method 4** for new retention time of CAFPIPARM-Nitrile: 7.2 min.

Peptide CAFPIPARM-Thiazoline: **HRMS (ESI)** m/z: $[M + H]^+$ Calcd 969.4797, Found 969.4830; $[M + 2H]^{2+}$ Calcd 485.2435, Found 485.2455. **Analytical HPLC Method 4**: retention time of 8.4 min. Conversion: >99%.

Analytical HPLC Method 4 analysis of the crude reaction converting CAFPIPARM-Amide to CAFPIPARM-Nitrile

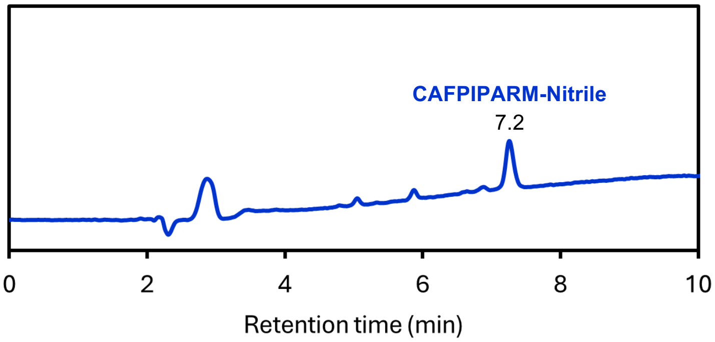

Analytical HPLC trace of the crude reaction converting CAFPIPARM-Nitrile to CAFPIPARM-Thiazoline

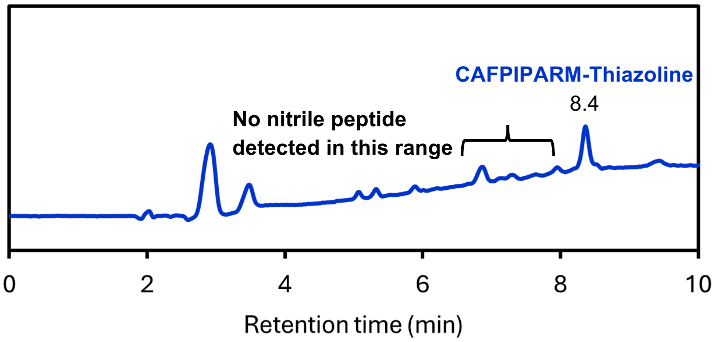

HRMS spectrum of fraction 8.4 min confirming the formation of CAFPIPARM -Thiazoline

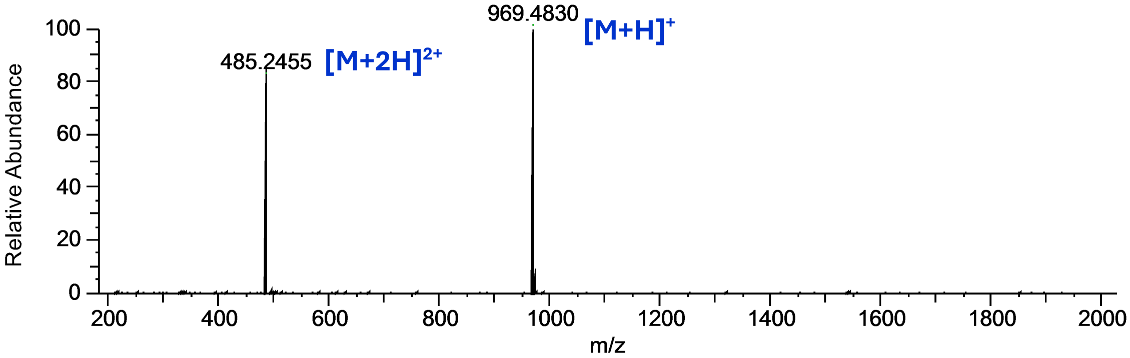

Entry 4

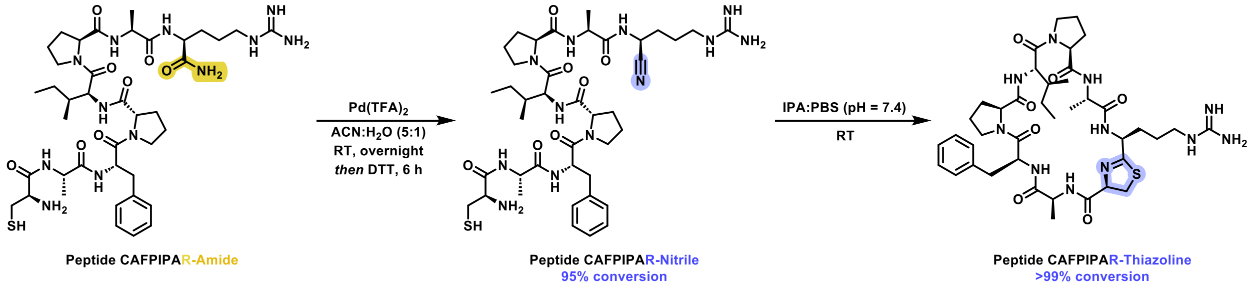

Peptide CAFPIPAR-Amide: **HRMS (ESI)** m/z: $[M + 2H]^{2+}$ Calcd 437.2418, Found 437.2433. **Analytical HPLC Method 1a**: retention time of 10.1 min.

Peptide CAFPIPAR-Nitrile: **HRMS (ESI)** m/z: $[M + 2H]^{2+}$ Calcd 428.2366, Found 428.2381. **Analytical HPLC Method 1a**: retention time of 11 min. Conversion: 95%.

Because the formation of **CAFPIPAR-Thiazoline** was analyzed using **Analytical HPLC Method 4**, the crude reaction converting CAFPIPAR-Amide to CAFPIPAR-Nitrile was analyzed again using **Analytical HPLC Method 4** for new retention time of CAFPIPAR-Nitrile: 6.6 min.

Peptide CAFPIPAR-Thiazoline: **HRMS (ESI)** m/z: $[M + H]^{+}$ Calcd 838.4392, Found 838.4416; $[M + 2H]^{2+}$ Calcd 419.7232, Found 419.7247. **Analytical HPLC Method 4**: retention time of 8.1 min. Conversion: >99%.

Analytical HPLC trace of the purified peptide CAFPIPAR-Amide

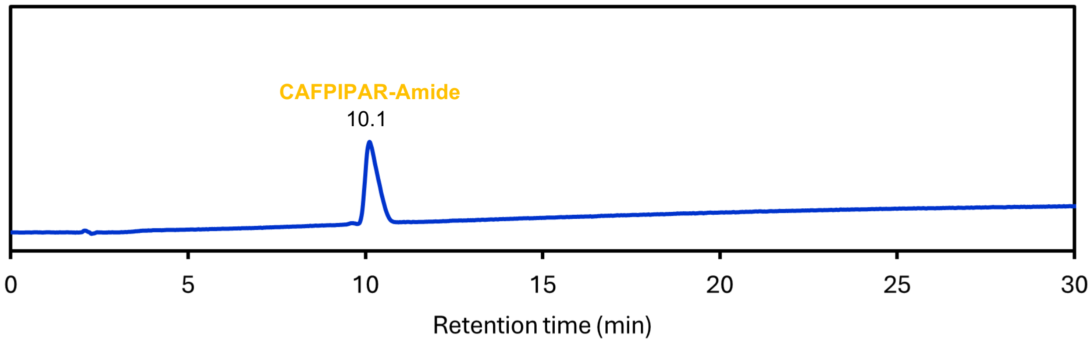

HRMS spectrum of the purified peptide CAFPIPAR-Amide

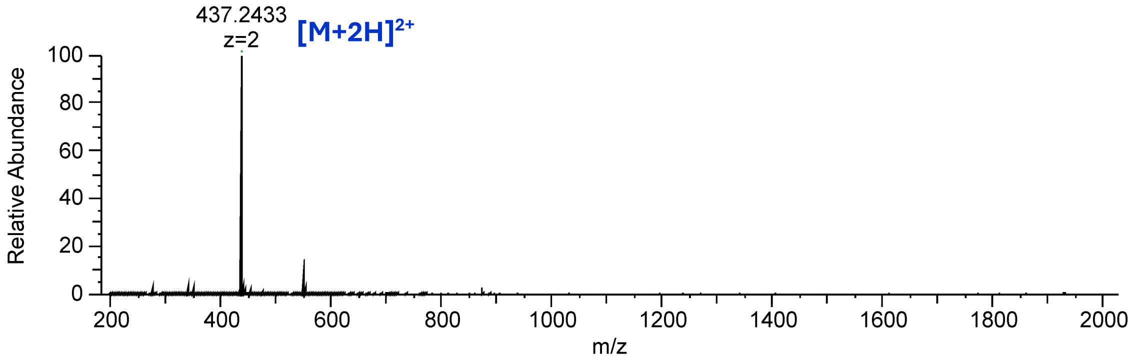

Analytical HPLC trace of the crude reaction converting CAFPIPAR-Amide to CAFPIPAR-Nitrile

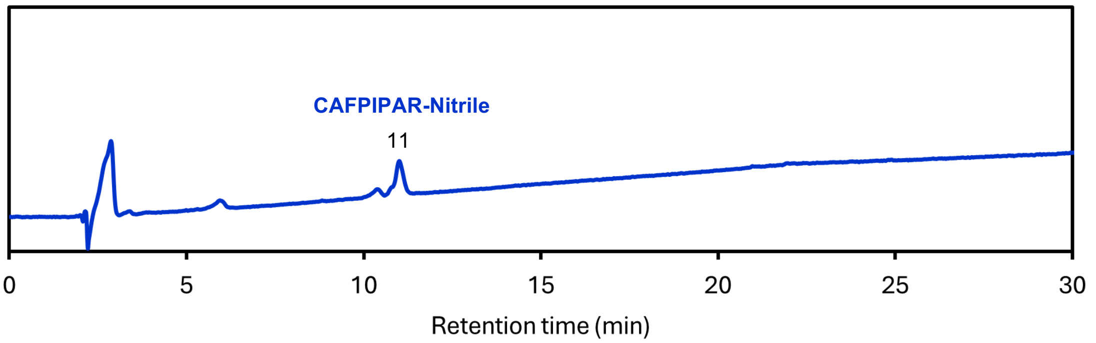

HRMS spectrum of fraction 11 min confirming the formation of CAFPIPAR-Nitrile

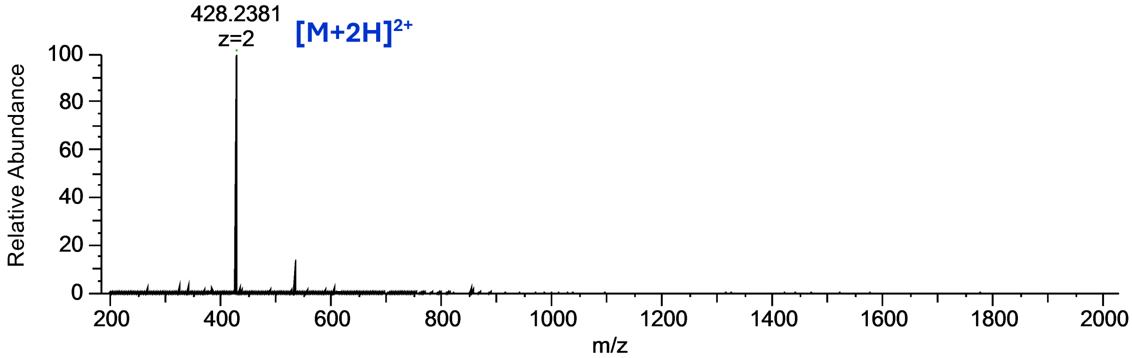

Analytical HPLC Method 4 analysis of the crude reaction converting CAFPIPAR-Amide to CAFPIPAR-Nitrile

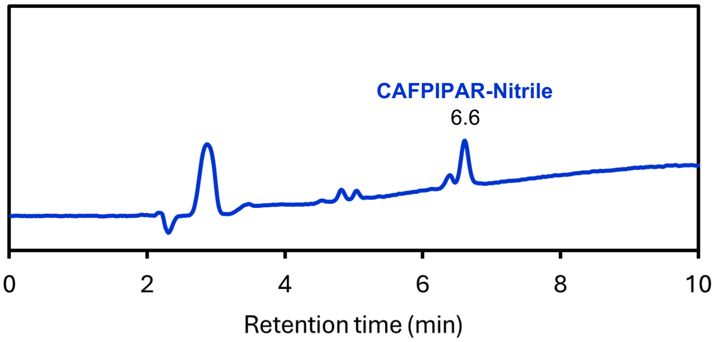

Analytical HPLC trace of the crude reaction converting CAFPIPAR-Nitrile to CAFPIPAR-Thiazoline

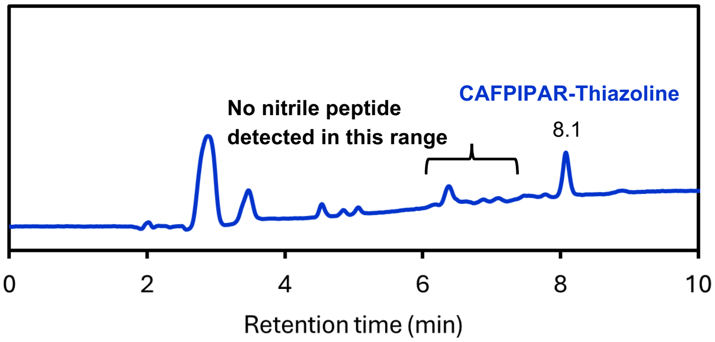

HRMS spectrum of fraction 8.1 min confirming the formation of CAFPIPAR-Thiazoline

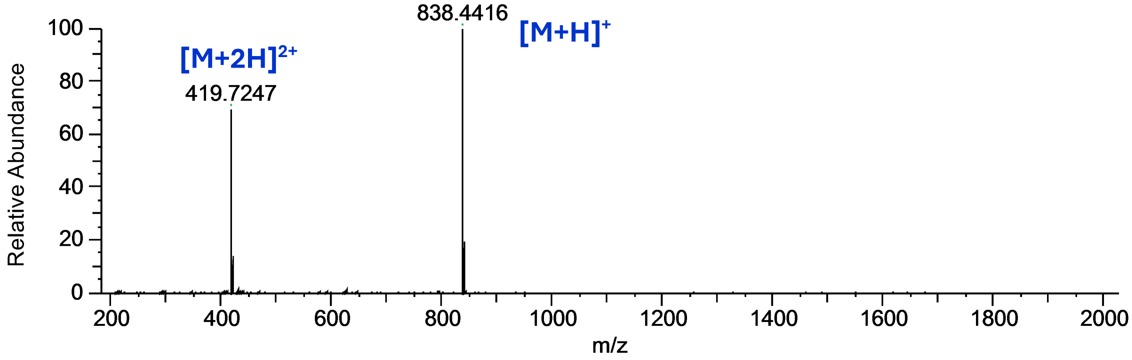

XXVII. Supplementary Figure 15. Converting thiazoline peptide 1c to thiazole peptide 1d.

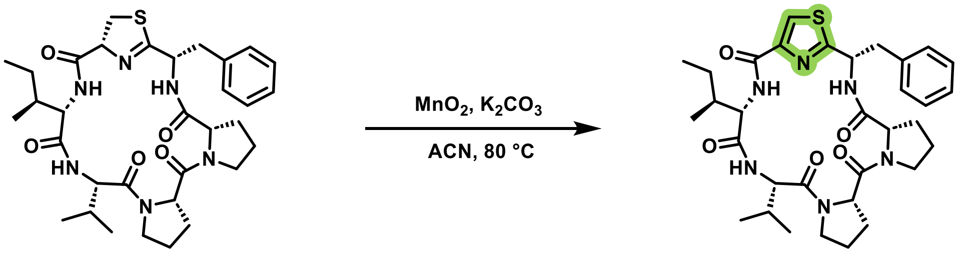

Procedure

The thiazoline peptide **1c** (1 mg, 0.0016 mmol) was subjected to general procedure B to afford the thiazole peptide **1d** (>99% conversion).

Peptide 1d: **HRMS (ESI)** m/z: $[M + H]^+$ Calcd 637.3172, Found 637.3166; $[M + Na]^+$ Calcd 659.2992, Found 659.2985; $[M + K]^+$ Calcd 675.2731, Found 675.2722. **Analytical HPLC Method 1b**: retention time of 20.5 min.

Analytical HPLC trace of crude reaction converting thiazoline peptide 1c to thiazole peptide 1d

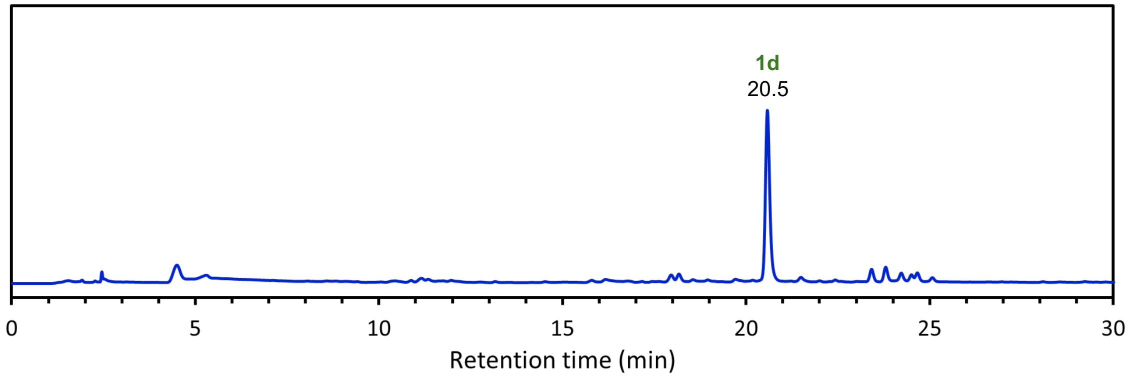

HRMS spectrum of fraction 20.5 min confirming the formation of thiazole peptide 1d

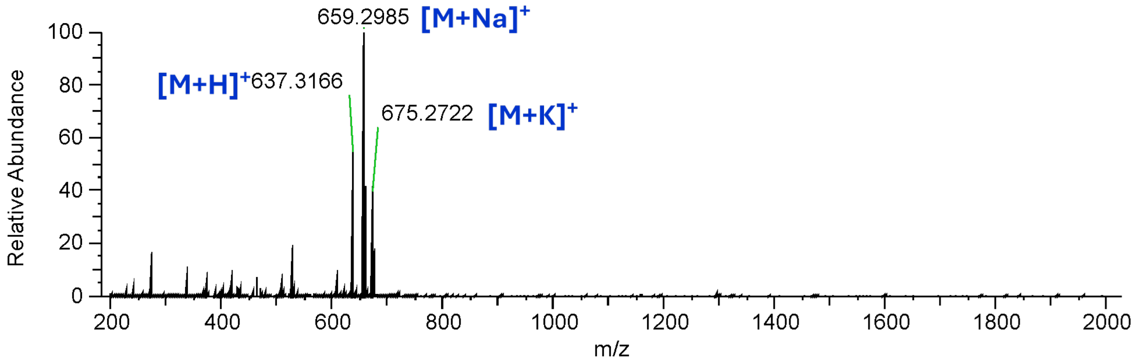

XXVIII. Supplementary Figure 16. Converting thiazoline peptide 2c to thiazole peptide 2d (Sanguinamide A).

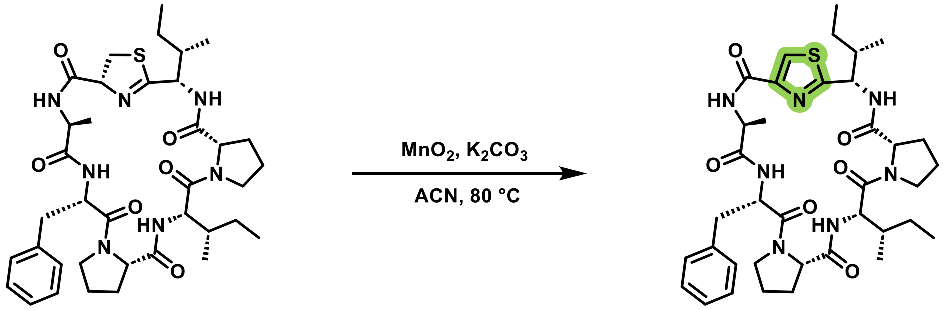

2c

2d - Sanguinamide A
>99% conversion

Procedure

The thiazoline peptide **2c** (1 mg, 0.0014 mmol) was subjected to general procedure B to afford the thiazole peptide **2d** (>99% conversion).

Peptide 2d (Sanguinamide A): **HRMS (ESI)** m/z: $[M + H]^+$ Calcd 722.3700, Found 722.3695; $[M + Na]^+$ Calcd 744.3520, Found 744.3512; $[M + K]^+$ Calcd 760.3259, Found 760.3253. **Analytical HPLC Method 1b**: retention time of 22.1 min.

Analytical HPLC trace of crude reaction converting thiazoline peptide 2c to thiazole peptide 2d (Sanguinamide A)

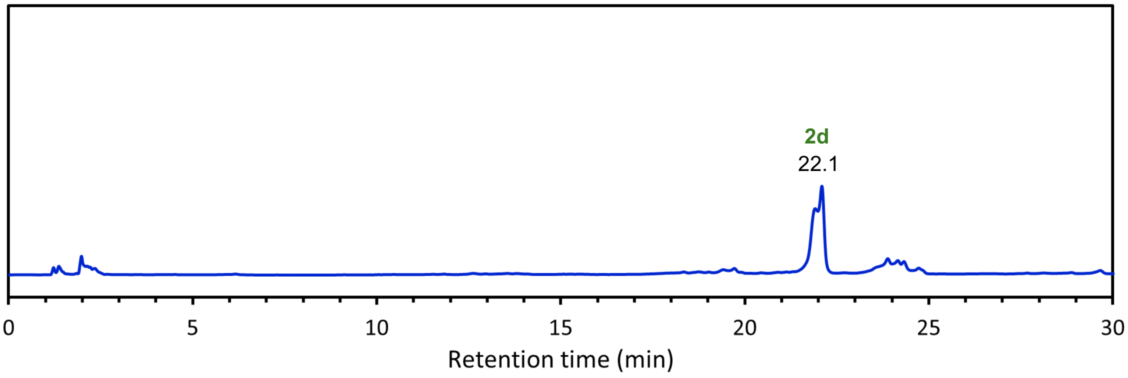

HRMS spectrum of fraction 22.1 min confirming the formation of thiazole peptide 2d (Sanguinamide A)

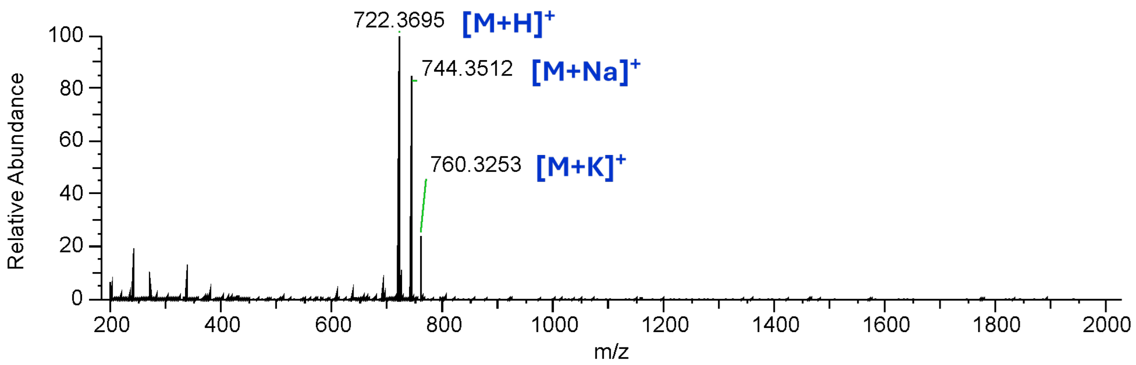

XXIX. Supplementary Figure 17. Converting thiazoline peptide 3c to thiazole peptide 3d (Sanguinamide A analog).

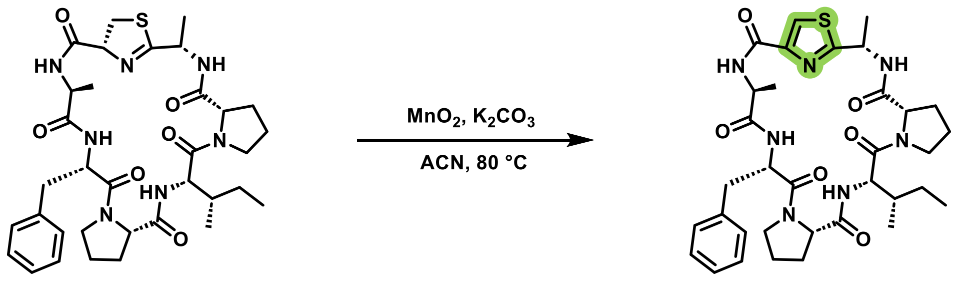

3c

3d - Sanguinamide A analog
>99% conversion

Procedure

The thiazoline peptide **3c** (1 mg, 0.0015 mmol) was subjected to general procedure B to afford the thiazole peptide **3d** (>99% conversion).

Peptide 3d (Sanguinamide A analog): **HRMS (ESI)** m/z: $[M + H]^+$ Calcd 680.3230, Found 680.3225; $[M + Na]^+$ Calcd 702.3050, Found 702.3041. **Analytical HPLC Method 1b**: retention time of 19 min.

Analytical HPLC trace of crude reaction converting thiazoline peptide 3c to thiazole peptide 3d (Sanguinamide A analog)

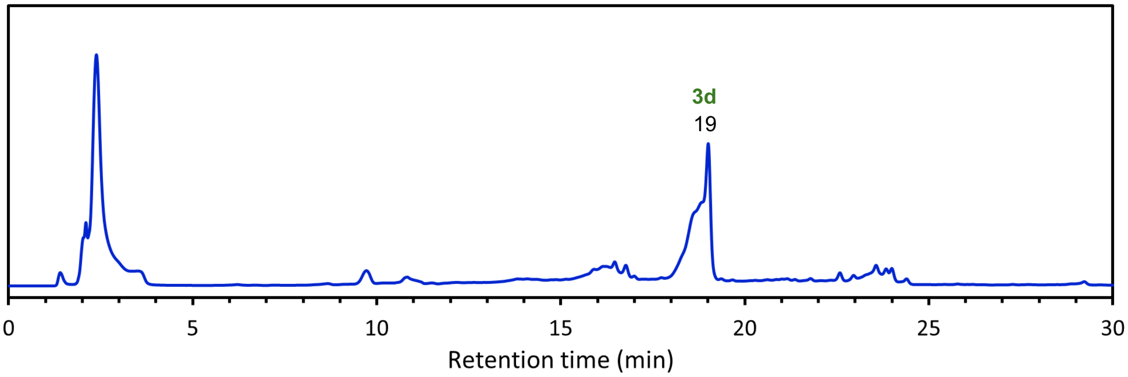

HRMS spectrum of fraction 19 min confirming the formation of thiazole peptide 3d

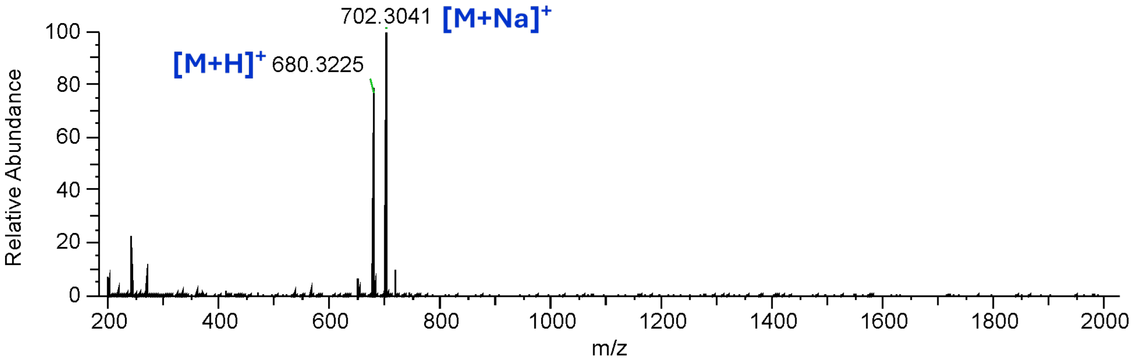

XXX. Supplementary Figure 18. Converting thiazoline peptide 4c to thiazole peptide 4d.

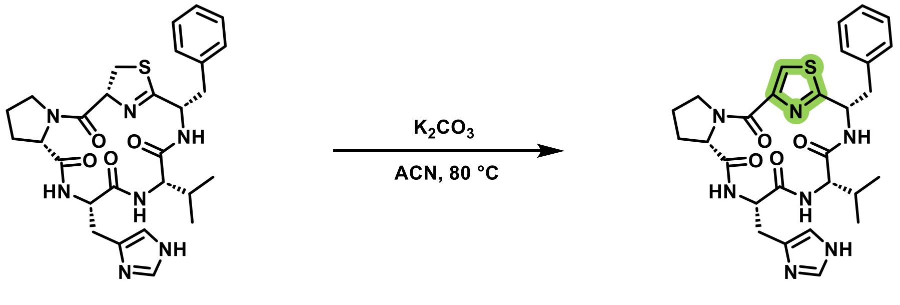

Procedure

The thiazoline peptide **4c** (1 mg, 0.0015 mmol) was subjected to general procedure B without MnO_2 to afford the thiazole peptide **4d** (>99% conversion).

Peptide 4d: **HRMS (ESI)** m/z: $[M + H]^+$ Calcd 564.2393, Found 564.2387; $[M + Na]^+$ Calcd 586.2213, Found 586.2206. **Analytical HPLC Method 3b**: retention time of 20.5 min.

Analytical HPLC trace of the crude reaction converting thiazoline peptide 4c to thiazole peptide 4d

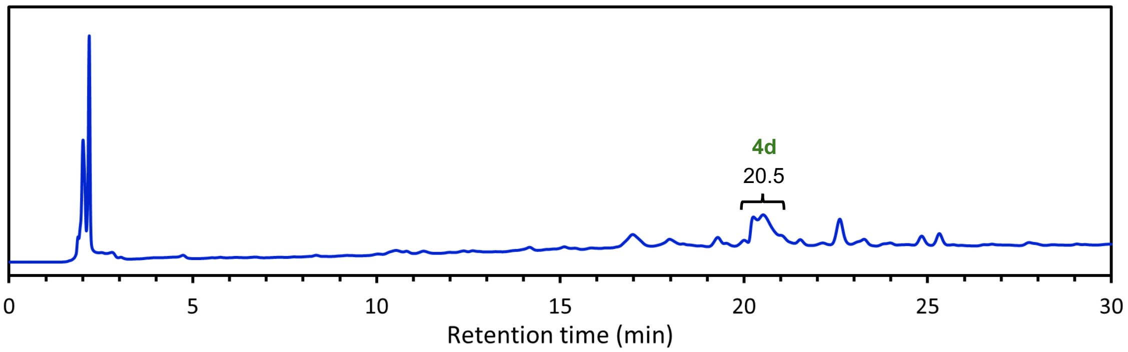

HRMS spectrum of fraction 20.5 min confirming the formation of thiazole peptide 4d

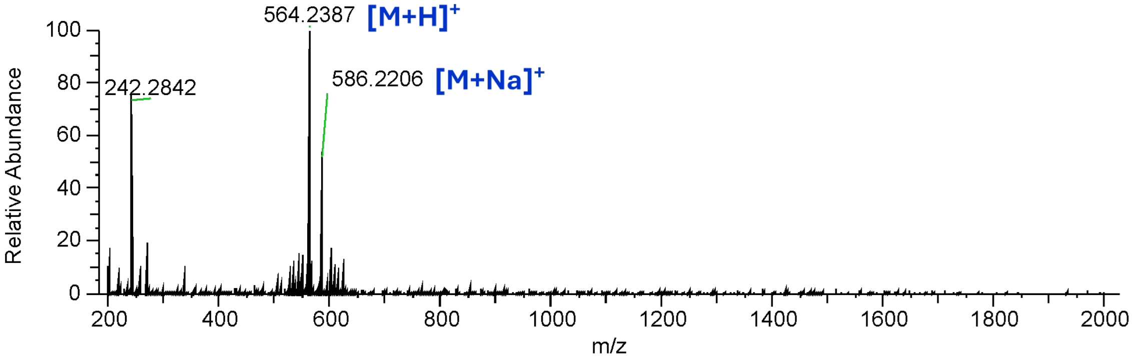

XXXI. Supplementary Figure 19. Converting thiazoline peptide 5c to thiazole peptide 5d (Haligramide A).

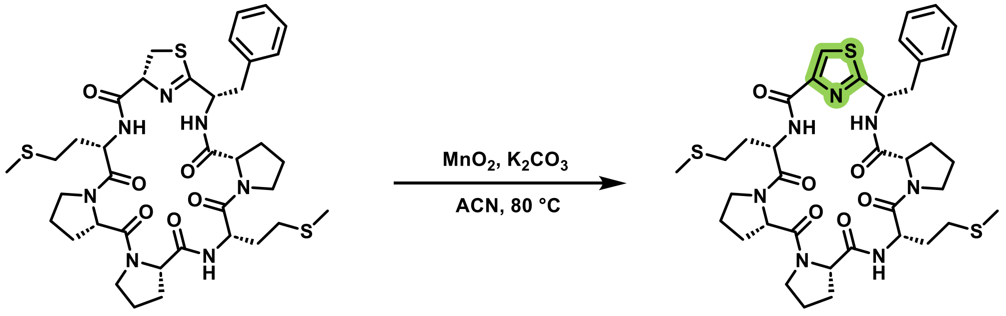

5c

5d - Haligramide A
>99% conversion

Procedure

The thiazoline peptide **5c** (1 mg, 0.0013 mmol) was subjected to general procedure B to afford the thiazole peptide **5d** (>99% conversion).

Peptide 5d (Haligramide A): **HRMS (ESI)** m/z: $[M + H]^+$ Calcd 784.2985, Found 784.2981; $[M + Na]^+$ Calcd 806.2804, Found 806.2796. **Analytical HPLC Method 1b**: retention time of 21 min.

Analytical HPLC trace of the crude reaction converting thiazoline peptide 5c to thiazole peptide 5d (Haligramide A)

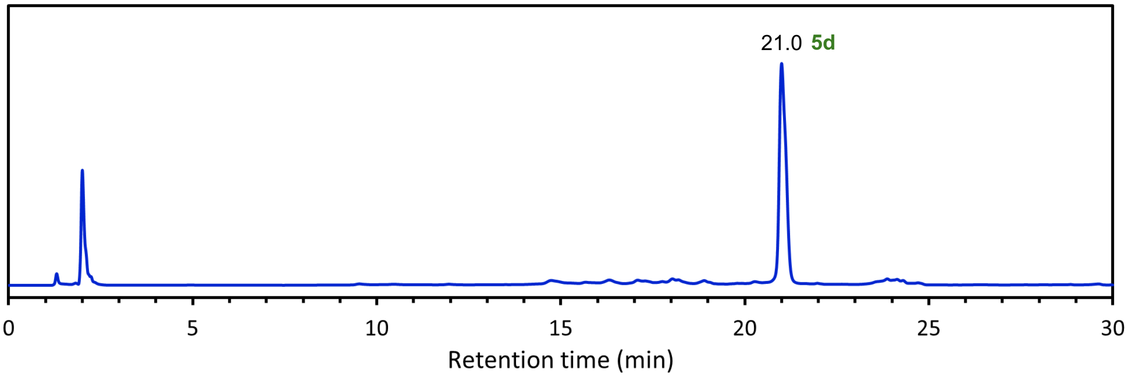

HRMS spectrum of fraction 21 min confirming the formation of the thiazole peptide 5d (Haligramide A)

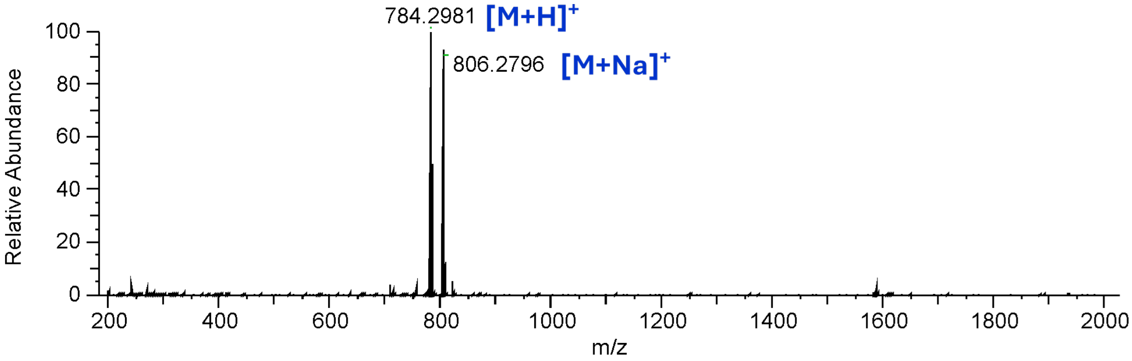

XXXII. Supplementary Figure 20. Converting thiazoline peptide 6c to thiazole peptide 6d.

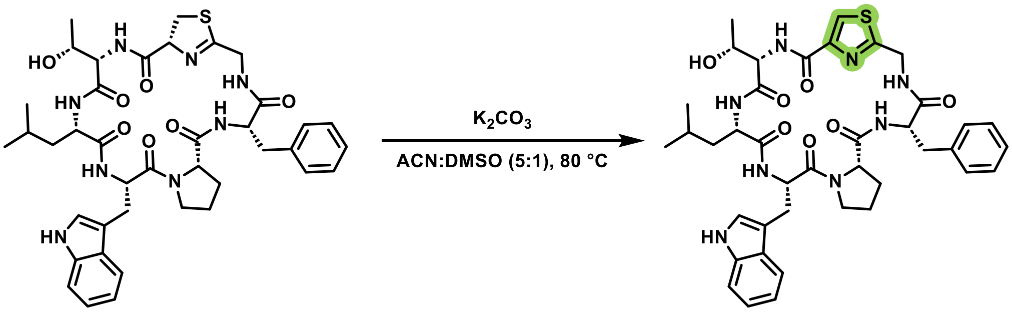

6c - Phakellistatin 13 analog

6d
>99% conversion

Procedure

The thiazoline peptide **6c** (1 mg, 0.0013 mmol) was subjected to general procedure B without MnO_2 to afford the thiazole peptide **6d** (>99% conversion). DMSO was utilized as a co-solvent (5:1 ACN:DMSO) to solubilize peptide **6c** for this reaction.

Peptide 6d: **HRMS (ESI)** m/z: $[M + H]^+$ Calcd 785.3445, Found 785.3440; $[M + Na]^+$ Calcd 807.3265, Found 807.3258. **Analytical HPLC Method 1b**: retention time of 19.1 min.

Analytical HPLC trace of the crude reaction converting thiazoline peptide 6c to thiazole peptide 6d

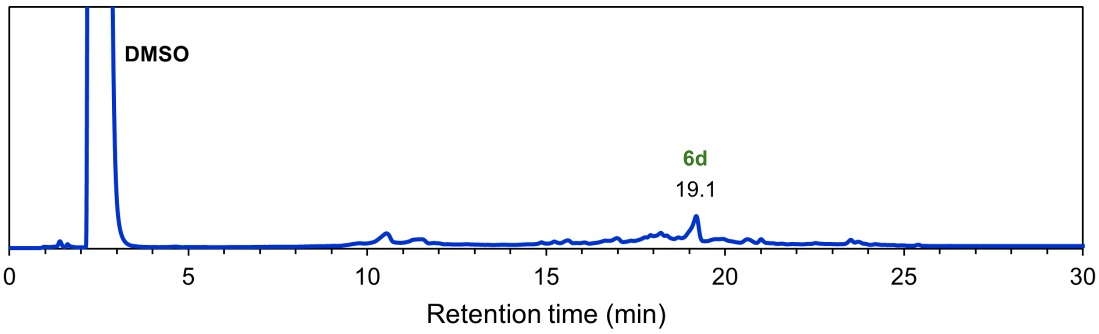

HRMS spectrum of fraction 19.1 min confirming the formation of the thiazole peptide 6d

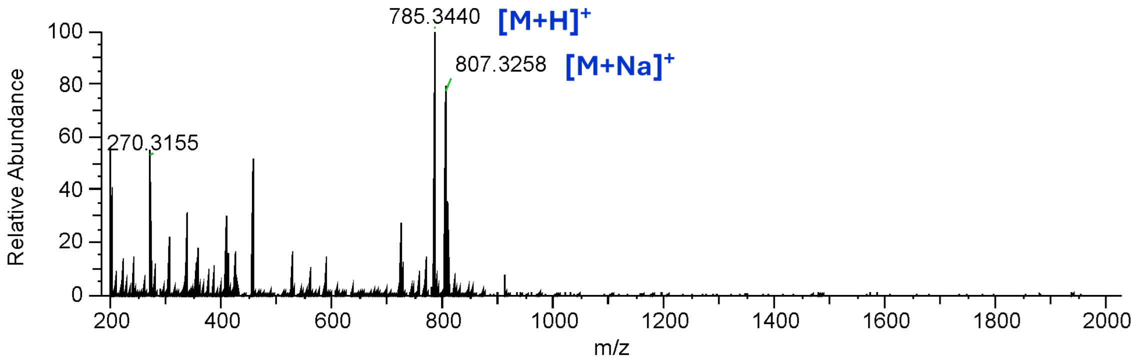

XXXIII. Supplementary Figure 21. Converting thiazoline peptide 1c to hydrolyzed peptide 1e.

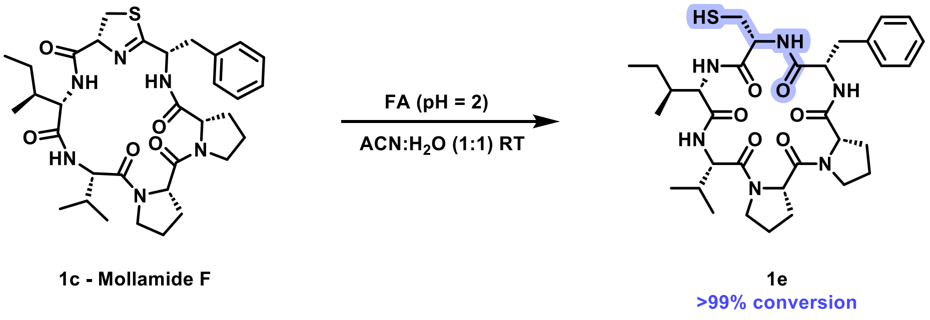

Procedure

The thiazoline peptide **1c** (1 mg, 0.0016 mmol) was subjected to general procedure C to afford the hydrolyzed peptide **1e** (>99% conversion).

Peptide 1e: **HRMS (ESI)** m/z: $[M + H]^+$ Calcd 657.3434, Found 657.3416; $[M + Na]^+$ Calcd 679.3254, Found 679.3231. **Analytical HPLC Method 1b**: retention time of 16.6 min.

Analytical HPLC trace of crude reaction converting thiazoline peptide 1c to hydrolyzed peptide 1e

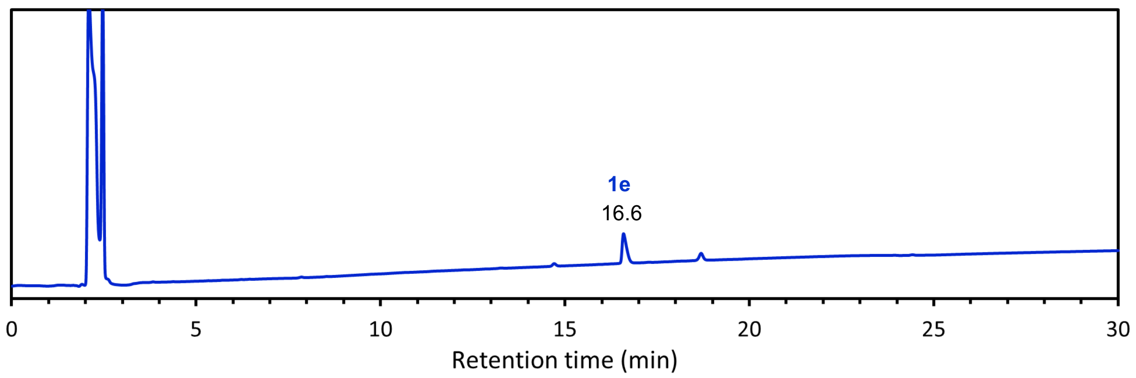

HRMS spectrum of fraction 16.6 min confirming the formation of hydrolyzed peptide 1e

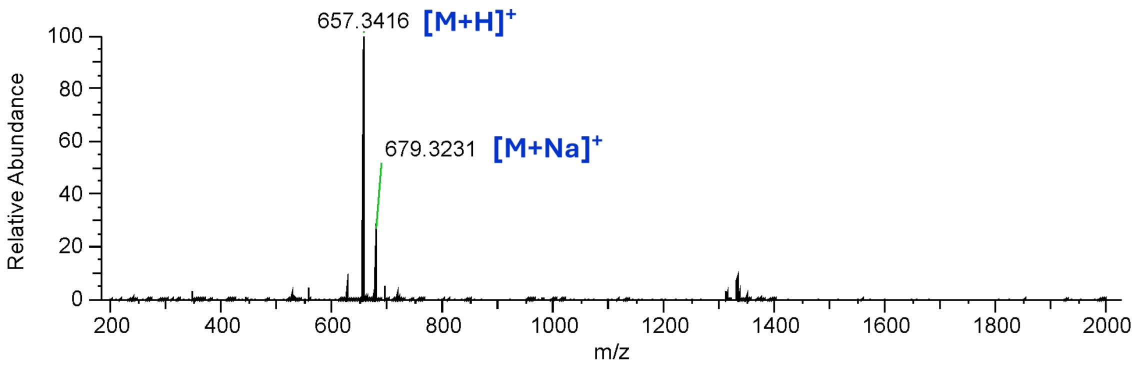

XXXIV. Supplementary Figure 22. Converting thiazoline peptide 2c to hydrolyzed peptide 2e.

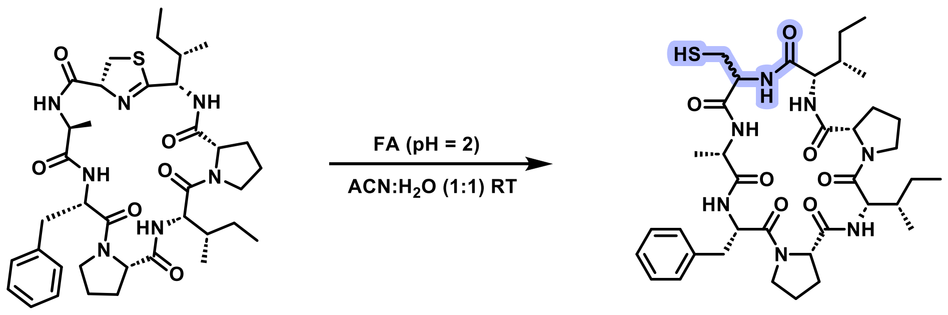

2c

2e
>99% conversion

Procedure

The thiazoline peptide **2c** (1 mg, 0.0014 mmol) was subjected to general procedure C to afford the hydrolyzed peptide **2e** (>99% conversion).

Peptide 2e: **HRMS (ESI)** m/z: $[M + H]^+$ Calcd 742.3962, Found 742.3958 and 742.3956; $[M + Na]^+$ Calcd 764.3782, Found 764.3773. **Analytical HPLC Method 1b**: two diastereomers at 16.2 and 18.1 min retention time.

Analytical HPLC trace of crude reaction converting thiazoline peptide 2c to hydrolyzed peptide 2e

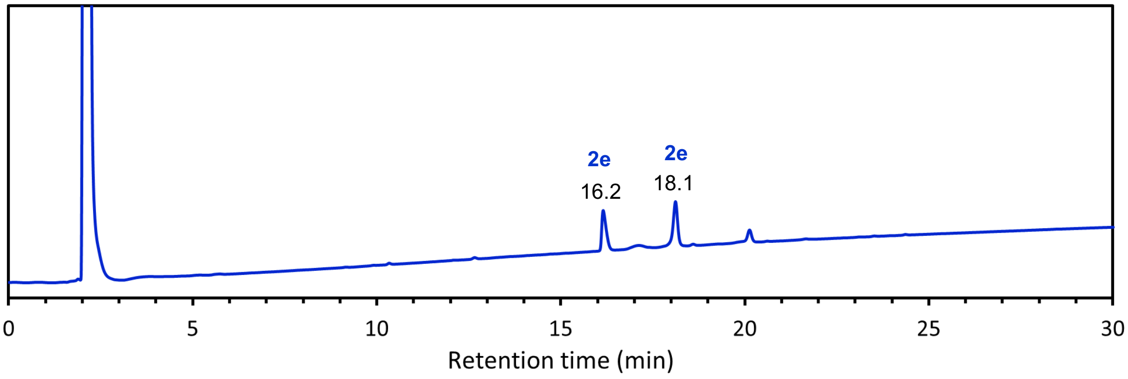

HRMS spectrum of fraction 16.2 min confirming the formation of hydrolyzed peptide 2e

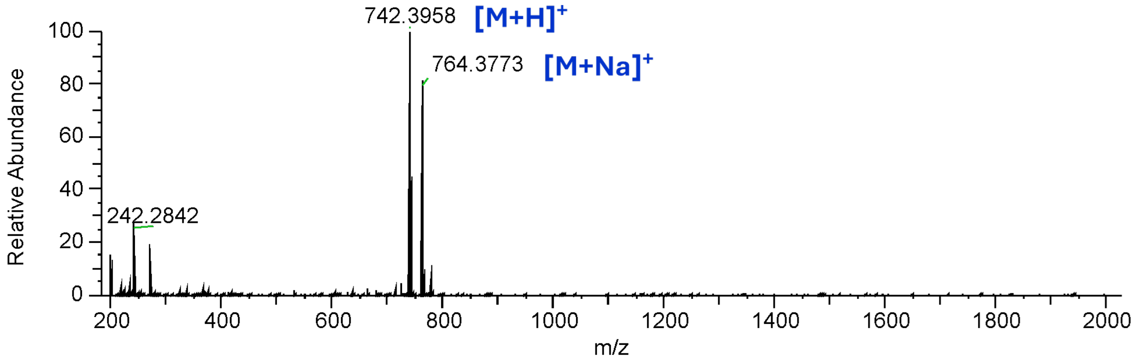

HRMS spectrum of fraction 18.1 min confirming the formation of hydrolyzed peptide 2e

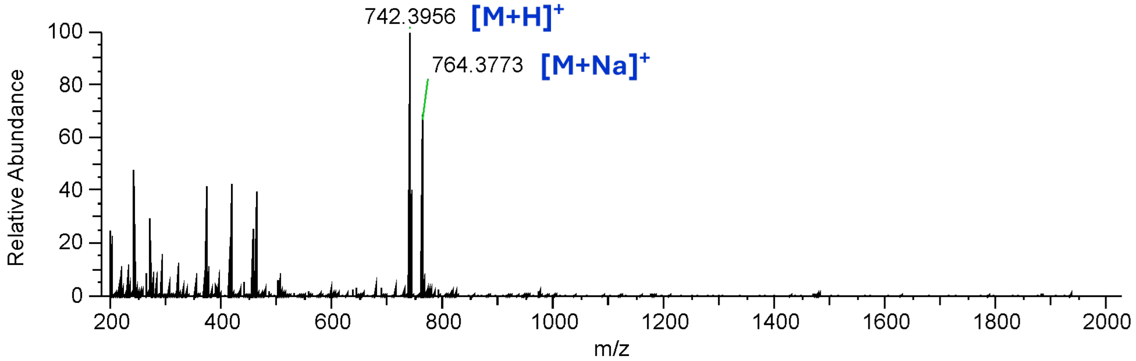

XXXV. Supplementary Figure 23. Converting thiazoline peptide 3c to hydrolyzed peptide 3e.

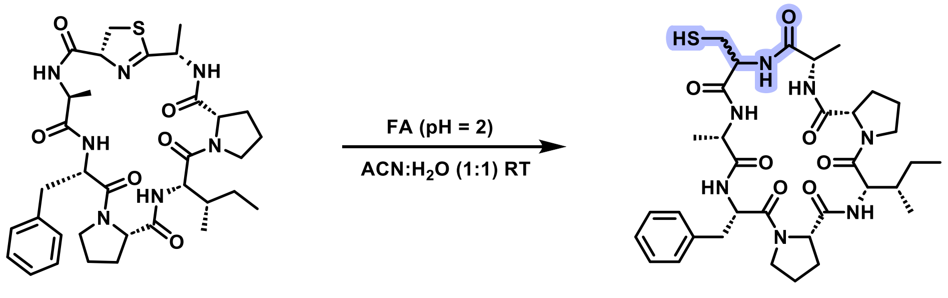

Procedure

The thiazoline peptide **3c** (1 mg, 0.0015 mmol) was subjected to general procedure C to afford the hydrolyzed peptide **3e** (>99% conversion).

Peptide 3e: **HRMS (ESI)** m/z: $[M + H]^+$ Calcd 700.3492, Found 700.3488; $[M + Na]^+$ Calcd 722.3312, Found 722.3305 and 722.3306. **Analytical HPLC Method 1b**: two diastereomers at 16 and 16.7 min retention time.

Analytical HPLC trace of crude reaction converting thiazoline peptide 3c to hydrolyzed peptide 3e

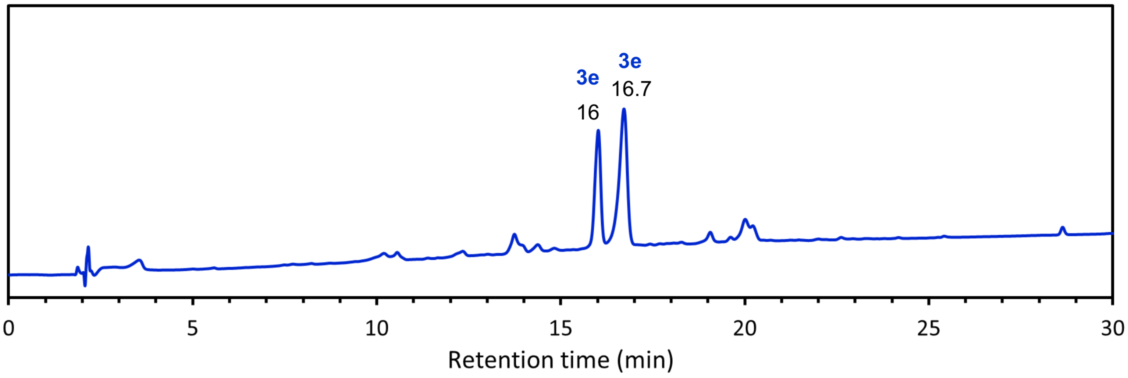

HRMS spectrum of fraction 16 min confirming the formation of hydrolyzed peptide 3e

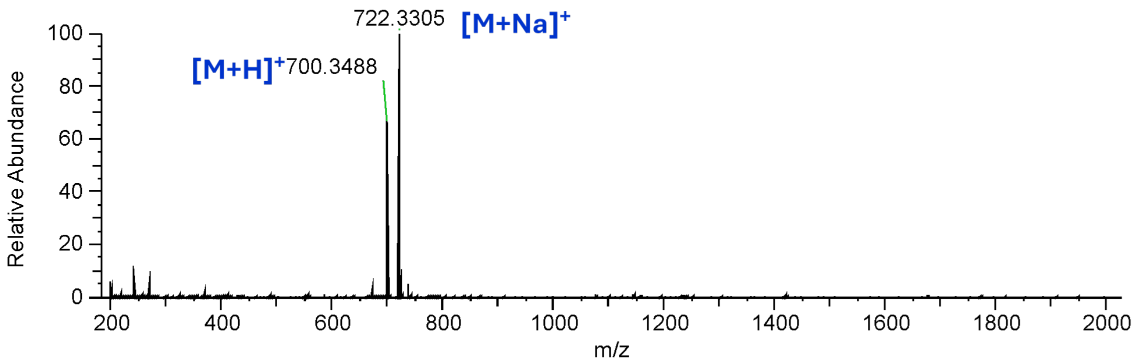

HRMS spectrum of fraction 16.7 min confirming the formation of hydrolyzed peptide 3e

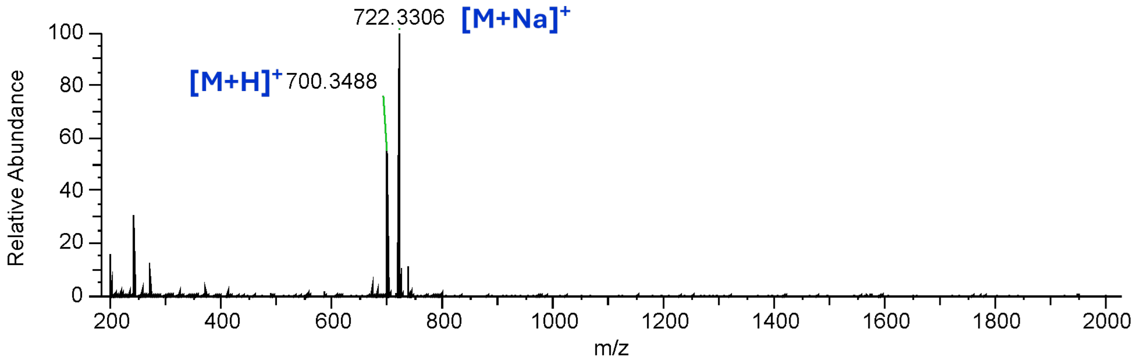

XXXVI. Supplementary Figure 24. Converting thiazoline peptide 4c to hydrolyzed peptide 4e.

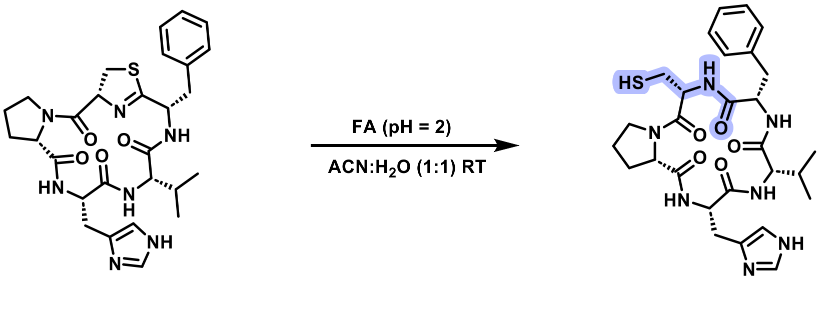

Procedure

The thiazoline peptide **4c** (1 mg, 0.0018 mmol) was subjected to general procedure C to afford the hydrolyzed peptide **4e** (>99% conversion).

Peptide 4e: **HRMS (ESI)** m/z: $[M + H]^+$ Calcd 584.2655, Found 584.2650; $[M + Na]^+$ Calcd 606.2475, Found 606.2469; $[M + K]^+$ Calcd 622.2214, Found 622.2209. **Analytical HPLC Method 3b**: retention time of 16.1 min.

Analytical HPLC trace of the crude reaction converting thiazoline peptide 4c to hydrolyzed peptide 4e

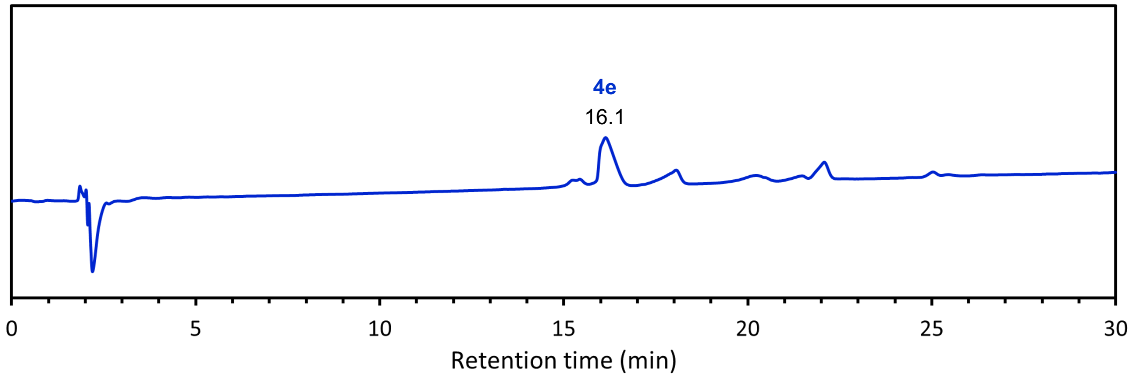

HRMS spectrum of fraction 16.1 min confirming the formation of hydrolyzed peptide 4e

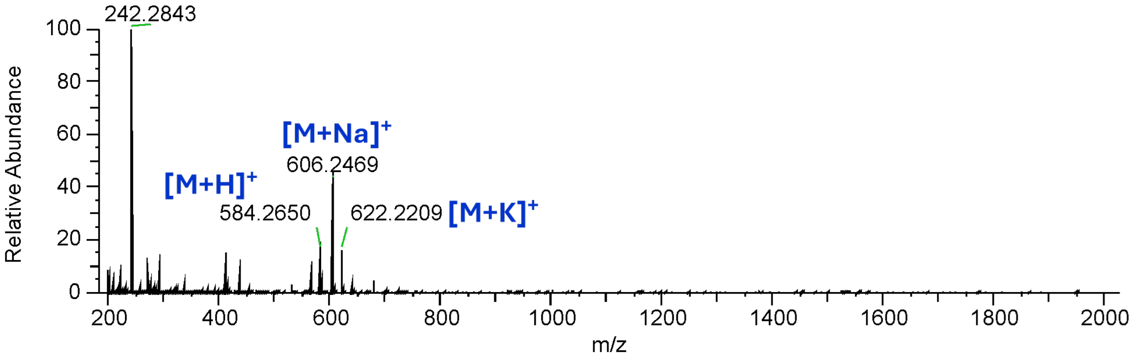

XXXVII. Supplementary Figure 25. Converting thiazoline peptide 5c to hydrolyzed peptide 5e.

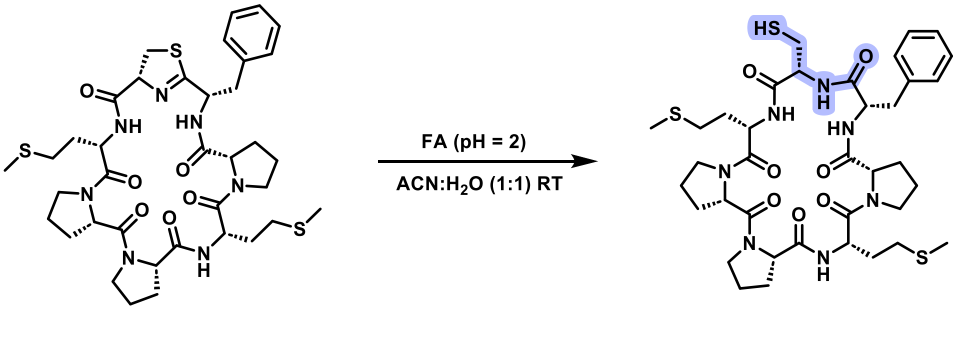

Procedure

Instead of purifying the thiazoline peptide **5c**, FA (20 µL) was added directly to the crude reaction mixture following the formation of **5c** via general procedure A. The resulting mixture was allowed to react overnight at room temperature to afford the hydrolyzed peptide **5e** (>99% conversion).

Peptide 5e: **HRMS (ESI)** m/z: $[M + H]^+$ Calcd 804.3247, Found 804.3244; $[M + Na]^+$ Calcd 826.3057, Found 826.3058. **Analytical HPLC Method 1b**: retention time of 18.2 min.

Analytical HPLC trace of the crude reaction converting thiazoline peptide 9c to hydrolyzed peptide 9e

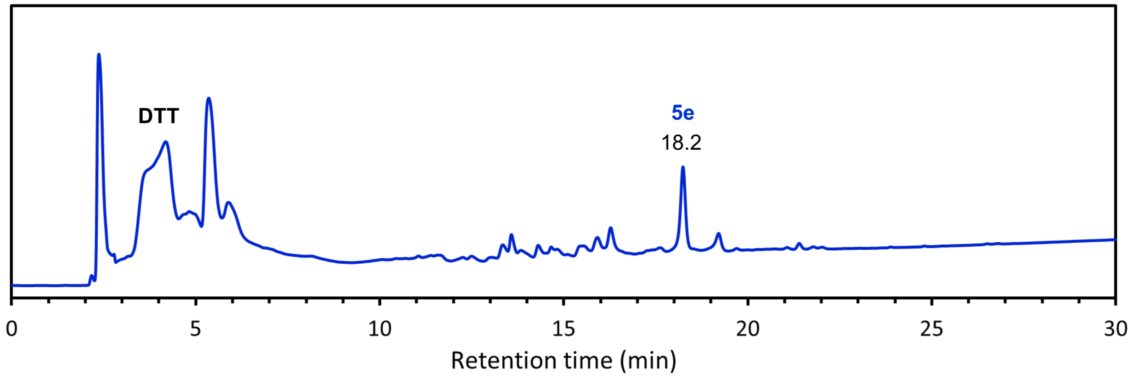

HRMS spectrum of fraction 18.2 min confirming the formation of the hydrolyzed peptide 5e

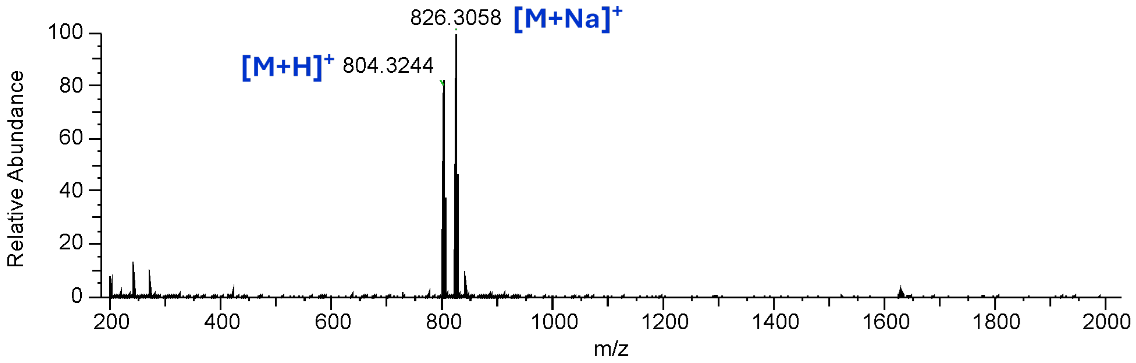

XXXVIII. Supplementary Figure 26. Converting thiazoline peptide 6c to hydrolyzed peptide 6e.

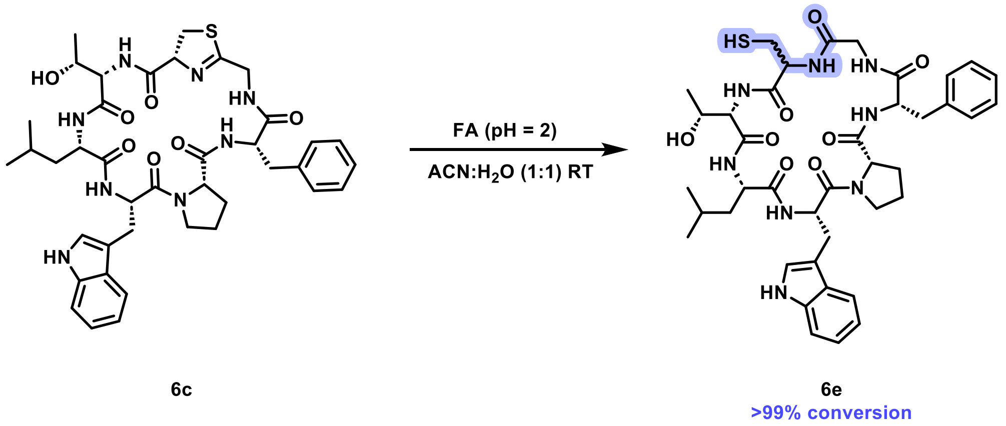

Procedure

The thiazoline peptide **6c** (1 mg, 0.0013 mmol) was subjected to general procedure C to afford the hydrolyzed peptide **6e** (>99% conversion).

Peptide 6e: **HRMS (ESI)** m/z: $[M + H]^+$ Calcd 805.3707, Found 805.3702 and 805.3706; $[M + Na]^+$ Calcd 827.3527, Found 827.3519 and 827.3521. **Analytical HPLC Method 1b**: two diastereomers at 17 and 17.8 min retention time.

Analytical HPLC trace of the crude reaction converting thiazoline peptide 6c to hydrolyzed peptide 6e

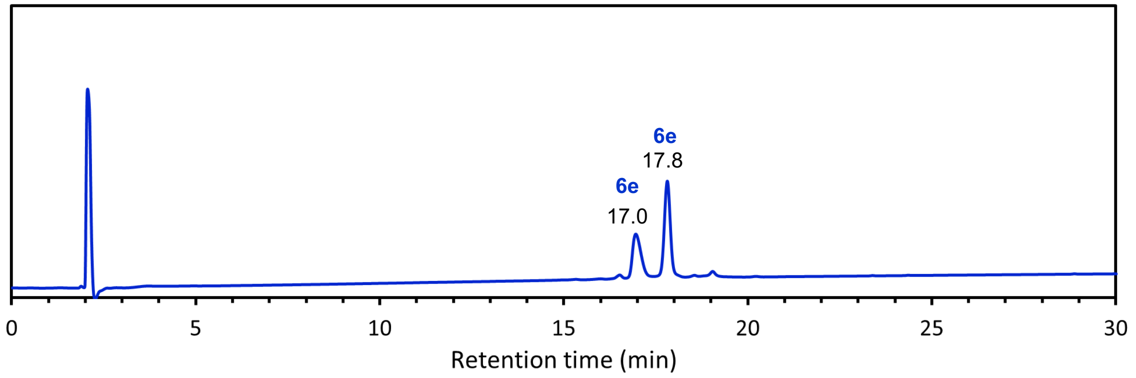

HRMS spectrum of fraction 17 min confirming the formation of the hydrolyzed peptide 6e

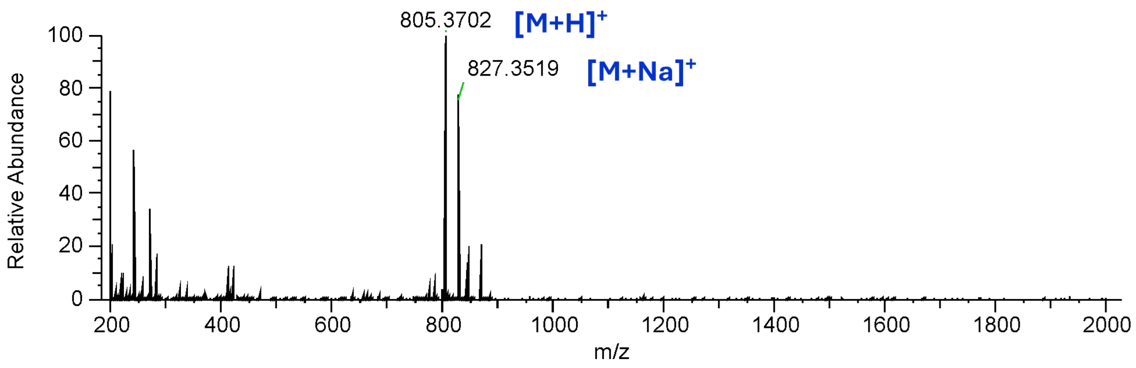

HRMS spectrum of fraction 17.8 min confirming the formation of the hydrolyzed peptide 6e

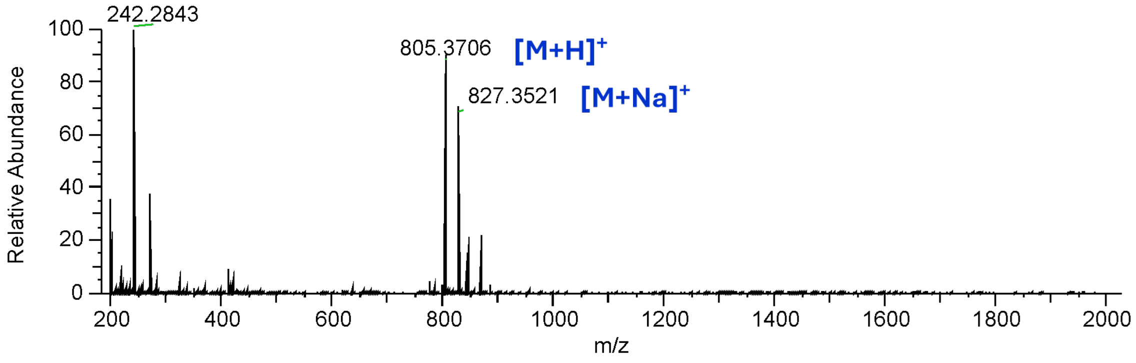

References

(1) Chan, W. C.; White, P. D. Fmoc solid phase peptide synthesis: A practical approach (Oxford Univ. Press, New York, 2000).
